# H1FOO-DD promotes efficiency and uniformity in reprogramming to naive pluripotency

**DOI:** 10.1016/j.stemcr.2024.04.005

**Published:** 2024-05-02

**Authors:** Akira Kunitomi, Ryoko Hirohata, Mitsujiro Osawa, Kaho Washizu, Vanessa Arreola, Norikazu Saiki, Tomoaki M. Kato, Masaki Nomura, Haruko Kunitomi, Tokiko Ohkame, Yusuke Ohkame, Jitsutaro Kawaguchi, Hiroto Hara, Kohji Kusano, Takuya Yamamoto, Yasuhiro Takashima, Shugo Tohyama, Shinsuke Yuasa, Keiichi Fukuda, Naoko Takasu, Shinya Yamanaka

**Affiliations:** 1 Center for iPS Cell Research and Application (CiRA), Kyoto University, Kyoto 606-8507, Japan; 2 Gladstone Institute of Cardiovascular Disease, San Francisco, CA 94158, USA; 3 CiRA Foundation, Kyoto 606-8397, Japan; 4 ID Pharma Co., Ltd, Ibaraki 300-2611, Japan; 5 Institute for the Advanced Study of Human Biology (WPI-ASHBi), Kyoto University, Kyoto 606-8501, Japan; 6 Medical-risk Avoidance Based on iPS Cells Team, RIKEN Center for Advanced Intelligence Project (AIP), Kyoto 606-8507, Japan; 7 Department of Cardiology, Keio University School of Medicine, Tokyo 160-8582, Japan; 8 Department of Anatomy, University of California, San Francisco, San Francisco, CA 94143, USA

**Keywords:** H1FOO, destabilized domain, Sendai virus vector, reprogramming, primed pluripotency, naive pluripotency, induced pluripotent stem cell, heterogeneity, innate immune response, FKBP1A

## Abstract

Heterogeneity among both primed and naive pluripotent stem cell lines remains a major unresolved problem. Here we show that expressing the maternal-specific linker histone *H1FOO* fused to a destabilizing domain (*H1FOO-DD*), together with *OCT4*, *SOX2*, *KLF4*, and *LMYC*, in human somatic cells improves the quality of reprogramming to both primed and naive pluripotency. *H1FOO-DD* expression was associated with altered chromatin accessibility around pluripotency genes and with suppression of the innate immune response. Notably, *H1FOO-DD* generates naive induced pluripotent stem cells with lower variation in transcriptome and methylome among clones and a more uniform and superior differentiation potency. Furthermore, we elucidated that upregulation of *FKBP1A*, driven by these five factors, plays a key role in *H1FOO-DD*-mediated reprogramming.

## Highlights

• *H1FOO-DD* improves both human primed and naive iPSC reprogramming efficiency
• *H1FOO-DD* alters chromatin accessibility that enhances iPSC reprogramming
• *H1FOO-DD* notably enhances the generation of higher-quality naive iPSCs
• *FKBP1A* plays a key role in *H1FOO-DD*-mediated iPSC reprogramming

## Graphical abstract

**Figure:**
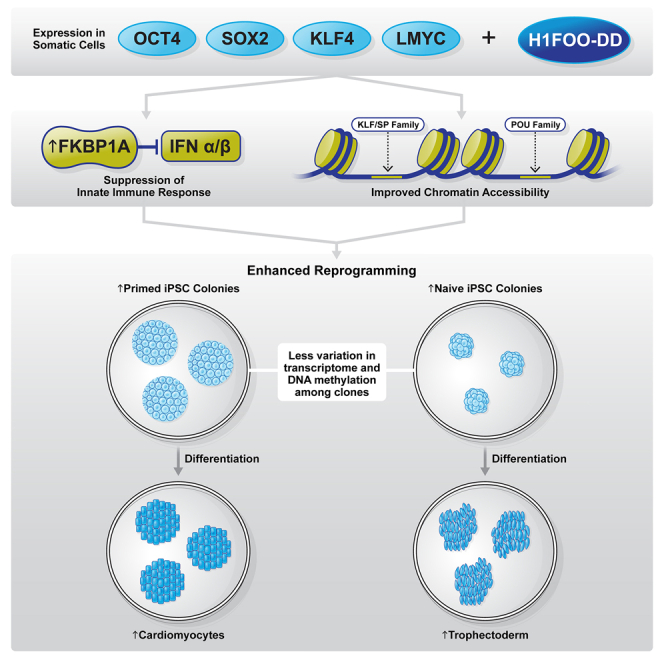

## Teaser

Kunitomi et al. showed that expressing the maternal-specific linker histone *H1FOO* fused to a destabilizing domain (*H1FOO-DD*), together with *OCT4*, *SOX2*, *KLF4*, and *LMYC*, in human somatic cells, improved the quality of reprogramming to both primed and naive pluripotency. This approach enables generation of human primed and naive iPSCs that are more homogenous and have improved differentiation potential.

## Introduction

Human induced pluripotent stem cells (iPSCs) generated from somatic cells using conventional reprogramming methods exhibit “primed pluripotency,” in that like the post-implantation epiblast; their differentiation into extraembryonic tissues is limited. On the other hand, human iPSCs with “naive pluripotency” can differentiate into both embryonic and extra-embryonic lineages, demonstrating characteristics similar to preimplantation epiblast cells and mouse iPSCs (Nichols and Smith, 2009; Weinberger et al., 2016). This capacity of naive pluripotent cells is critical for research into developmental biology and regenerative medicine, such as efforts to create blastoids and placentas (Io et al., 2021; Liu et al., 2021; Yu et al., 2021). Recently, methods for generating naive human iPSCs by introducing reprogramming factors into somatic cells using Sendai virus vectors have been reported (Kilens et al., 2018; Kunitomi et al., 2022; Liu et al., 2017) that will greatly advance these efforts.

However, both primed and naive iPSCs still face challenges for use in basic research and clinical applications (Yamanaka, 2020). In addition to the low generation efficiency, heterogeneity among cell lines, such as in gene expression, DNA methylation, and differentiation potency, is a critical issue because it can cause problems with the reproducibility of iPSC-based research and the safety of regenerative medicine using iPSC-derived differentiated cells. Notably, heterogeneity occurs even in reprogramming from the same somatic cells with the same genetic background (Francesconi et al., 2019), suggesting that conventional reprogramming methods are stochastically incomplete in some cells. Moreover, while there are many reports of efforts to improve the efficiency of primed iPSC colony generation (Liu et al., 2020), there are almost no reports of improved naive iPSC generation efficiency and very few reports of attempts to reduce human naive iPSC heterogeneity.

In this study, we focus on the maternally specific linker histone H1FOO, which is abundant in human oocytes and plays an important role in chromatin structural changes in the embryonic nucleus immediately after fertilization. We show that *H1FOO-DD*, which is a combination of *H1FOO* and a destabilizing domain (*DD*), enhances reprogramming to primed and naive human iPSCs. Adding *H1FOO-DD* to the set of reprogramming factors *OCT4*, *SOX2*, *KLF4*, and *LMYC* (OSKL) promotes the function of the *POU* family and *KLF/SP* family, which play important roles in the early stages of reprogramming. *H1FOO-DD* also drives *FKBP1A* upon co-expression with other reprogramming factors, resulting in suppression of the innate immune response, inflammatory response, and apoptosis during reprogramming. As a result, *H1FOO-DD* enhances the efficiency of reprogramming to a primed state and improves the differentiation potency of the generated iPSCs in some lineages. Moreover, *H1FOO-DD* also improves the reprogramming efficiency to a naive state and produces naive iPSCs with more uniform characteristics and superior differentiation potency.

## Results

### H1FOO-DD enhances reprogramming to primed pluripotency

**Figure 1:**
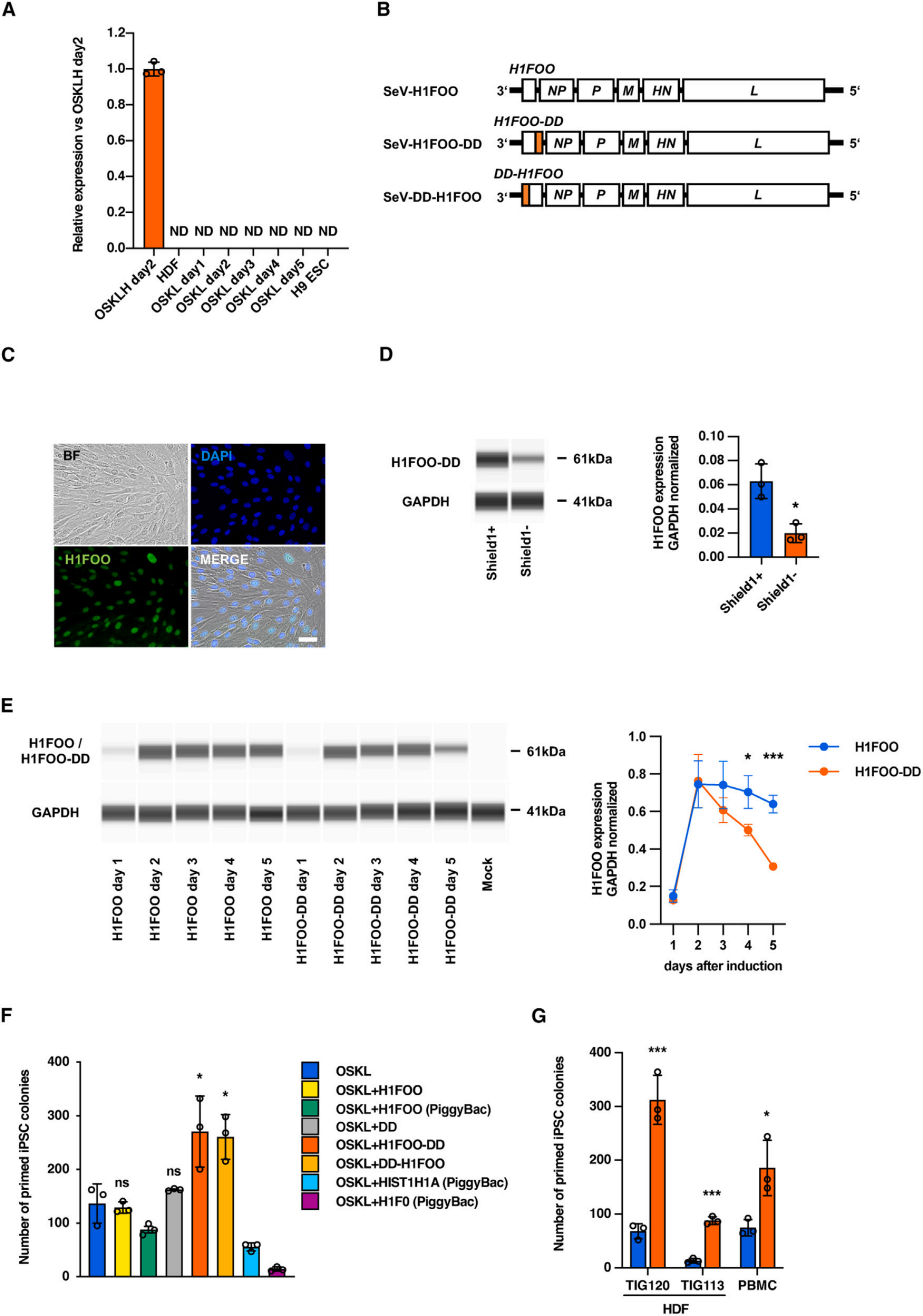
H1FOO-DD enhances reprogramming into primed pluripotency (A) qPCR analysis of *H1FOO* expression in HDFs, HDFs during reprogramming and H9 ESCs. Data are shown as the mean ± SD. *n* = 3. ND: not determined. (B) Schematic structure of the SeV-H1FOO vector and the modified vectors. We created vectors in which DD is added to the 5′ side (H1FOO-DD) or 3′ side (DD-H1FOO) of H1FOO for this study. (C) Representative phase-contrast image and immunofluorescent staining for H1FOO of HDFs at day 5 post SeV-H1FOO-DD infection. Scale bar, 50 μm. (D) Protein expression analysis of H1FOO-DD with Shield1 or without Shield1 by western blotting. Two days after the SeV vectors infection, cell culture temperature was raised from 35°C to 37°C to remove the SeV vectors. Three days after raising the temperature, 1 μM of Shield1 was added and the cells were collected the next day. We quantified the expression level of H1FOO-DD with GAPDH protein expression. Data are shown as the mean ± SD. *n* = 3. ^∗^*p* < 0.05. (E) Sequential protein expression analysis of H1FOO and H1FOO-DD by western blotting after the SeV-H1FOO or SeV-H1FOO-DD vector infection. We quantified the expression level of H1FOO and H1FOO-DD with GAPDH protein expression. Data are shown as the mean ± SD. *n* = 3. ^∗^*p* < 0.05, ^∗∗∗^*p* < 0.001. (F) Number of alkaline phosphatase (AP)-positive primed human iPSC colonies generated from HDFs at day 14. Each of the linker histone H1 related vectors were co-infected with SeV-OSKL. Data are shown as the mean ± SD. *n* = 3. ^∗^*p* < 0.05. (G) Number of AP-positive primed human iPSC colonies generated from HDFs and PBMC at day 14. We used CytoTune-EX-iPS vector for reprogramming in this experiment. Data are shown as the mean ± SD. *n* = 3. ^∗^*p* < 0.05.

We first examined the expression of *H1FOO* during the reprogramming of human dermal fibroblasts (HDFs). We used Sendai virus (SeV) vector for reprogramming the HDFs, which is one of the widely used methods for generating human iPSCs because of its high gene expression efficiency (Fusaki et al., 2009; Li et al., 2000). Moreover, we used *LMYC* instead of the proto-oncogene *CMYC*, which is reported to be more efficient in human iPSC reprogramming with OSKL (Akifuji et al., 2021; Nakagawa et al., 2008, 2010). No expression of *H1FOO* was observed in HDFs before or during the reprogramming process, up to day 5 after infection with OSKL, and *H1FOO* was also undetectable in H9 human embryonic stem cells (H9 ESCs) (Figure 1A). Thus, *H1FOO* is not endogenously expressed during the reprogramming of human iPSCs or in its end products, pluripotent stem cells (PSCs).

H1FOO is abundantly expressed in human oocytes, but its expression is known to be rapidly lost after fertilization (Ooga et al., 2016; Tanaka et al., 2001). Based on these findings, we hypothesized that we could recapitulate the developmental H1FOO expression pattern by constructing SeV vectors containing *H1FOO* with a system that rapidly degrades H1FOO. Therefore, we constructed not only a SeV-*H1FOO* vector which continuously and strongly expresses *H1FOO*, but also SeV-*H1FOO-DD* vectors with a destabilizing domain (DD) (Banaszynski et al., 2006) fused to the 3′ or 5′ side of *H1FOO* (SeV-*H1FOO-DD* and SeV-*DD*-*H1FOO*, respectively, Figure 1B). DD is a domain of the FKBP12 protein with a F36V mutation, and adding it to another protein results in rapid proteasomal degradation of the entire fusion product. Moreover, the addition of cell-permeable DD ligand named Shield1 protects DD-fusion proteins from degradation (Banaszynski et al., 2006). When HDFs were infected with SeV-*H1FOO-DD*, H1FOO localized to the nucleus (Figure 1C), and treatment with Shield1 significantly increased protein levels of H1FOO-DD (Figure 1D). This confirmed that DD promotes H1FOO degradation and that Shield1 works to prevent degradation of DD-fused protein as previously reported. Next, we sampled cells from day 1 to day 5 after SeV-H1FOO or SeV-H1FOO-DD infection and measured H1FOO protein expression levels. From day 2, H1FOO and H1FOO-DD were highly expressed, but H1FOO-DD degraded more rapidly than H1FOO, with significantly lower levels from day 4 onward (Figure 1E). This suggests that the DD-fused H1FOO protein is degraded and active in the cells for a shorter amount of time than un-tagged H1FOO.

Next, we examined if the addition of *H1FOO* or *DD*-fused *H1FOO* to OSKL improves the reprogramming efficiency of HDFs. To investigate whether the different vector backbones alter reprogramming efficiency, we also tested exogenous expression of OSKL plus *H1FOO* in the PiggyBac system. In addition, we overexpressed somatic H1 subtypes other than *H1FOO*, such as *HIST1H1A* and *H1F0* with OSKL using the PiggyBac system to check their effect. After introducing these vectors into HDFs, we compared the efficiency of iPSC generation on day 14 (Figure 1F). Regardless of vector type, neither *H1FOO* nor *DD* improved reprogramming efficiency, and somatic H1 markedly decreased the efficiency. In contrast, *H1FOO-DD* and *DD-H1FOO* significantly enhanced iPSC colony generation.

In the conventional method of iPSC generation using SeV vectors, iPSCs immediately after generation still express many SeV vector-derived pluripotency genes. Thus, some of the cells in the culture may have undergone incomplete reprogramming and be mixed in the generated iPSC population. To solve this problem and achieve a more accurate comparison of reprogramming efficiency, we used the CytoTuneEX-iPS kit for iPSC generation. With this approach, the SeV vectors are degraded and removed early after the generation of iPSCs because the target sequence of miR-367, which is specifically highly expressed in human PSCs (Zhang et al., 2015), is placed tandemly downstream of the P gene of the SeV vectors. Applying this approach to two types of HDFs and peripheral blood mononuclear cells (PBMCs) showed that *H1FOO-DD* markedly improved reprogramming efficiency in every cell type, with a maximum difference of about 8-fold compared to OSKL only (Figure 1G). This result suggests that *H1FOO-DD* generated more iPSCs regardless of the degree of transgene persistence. In summary, *H1FOO-DD* and *DD-H1FOO* have a positive effect on the generation of human primed iPSCs.

We then infected HDFs with OSKL or OSKL plus SeV-*H1FOO-DD* (OSKLH) and generated eight clones of iPSCs from each reprogramming cocktail to compare their characteristics. The OSKL group was infected with SeV-Mock instead of *H1FOO-DD* to achieve equal total multiplicity of infection (MOI). We first compared the transcriptome using bulk RNA sequencing (RNA-seq) and DNA methylation using DNA methylation array in these two groups as well as H9 ESCs and 201B7 iPSCs as PSC controls. Differentially expressed genes (DEGs) or differentially methylated regions were not detected between OSKL-iPSCs and OSKLH-iPSCs, but OSKLH-iPSCs tended to be relatively more aggregated in the transcriptome principal component analysis (PCA) than OSKL-iPSCs (Figure S1A). From this result, we hypothesized that the OSKLH-iPSCs may have less variation in gene expression among clones. To test this hypothesis, we calculated the number of genes with mean absolute error (MAE) of >2.0 in expression among clones in the same group, which we defined as high variability. Interestingly, the OSKLH-iPSCs had approximately half as many genes with high MAE as the OSKL-iPSCs, indicating that OSKLH-iPSCs has less variation in transcriptome than OSKL-iPSCs (Figure S1B). Then, we investigated Gene Ontology (GO) terms for the 1,350 genes with MAE >2.0 genes among the OSKL-iPSCs and found that many terms were related to gene expression (Figure S1C). We also compared the variability of DNA methylation with the same method and obtained results similar to transcriptome (Figure S1D). These analyses indicated that *H1FOO-DD* contributes to the generation of primed iPSC clones with less variation in gene expression and DNA methylation.

Next, we compared the trilineage differentiation potency of these iPSC clones in adherent culture using the STEMdiff Trilineage Differentiation Kit (Figure S1E) and compared their gene expression using Scorecard analysis (Tsankov et al., 2015), which examines the expression of 96 genes for each clone. There was no great difference between the two groups in ectoderm and mesoderm, but OSKLH-iPSCs showed a trend toward uniformly higher scores in endoderm, which is the most difficult differentiation to induce in this system (Figure S1F). Furthermore, we attempted terminal differentiation of the mesodermal lineage into cardiomyocytes. After 6 trials for each clone, we noted that OSKLH-iPSCs differentiated into cardiomyocytes with higher expression of TNNT2 (Figures S1G–S1I). In summary, OSKLH-iPSCs showed significantly higher potential for cardiomyocyte differentiation and slightly higher potential endoderm differentiation compared to OSKL-iPSCs.

### Single-cell RNA-seq analysis reveals improved reprogramming of OSKLH-infected cells

**Figure 2:**
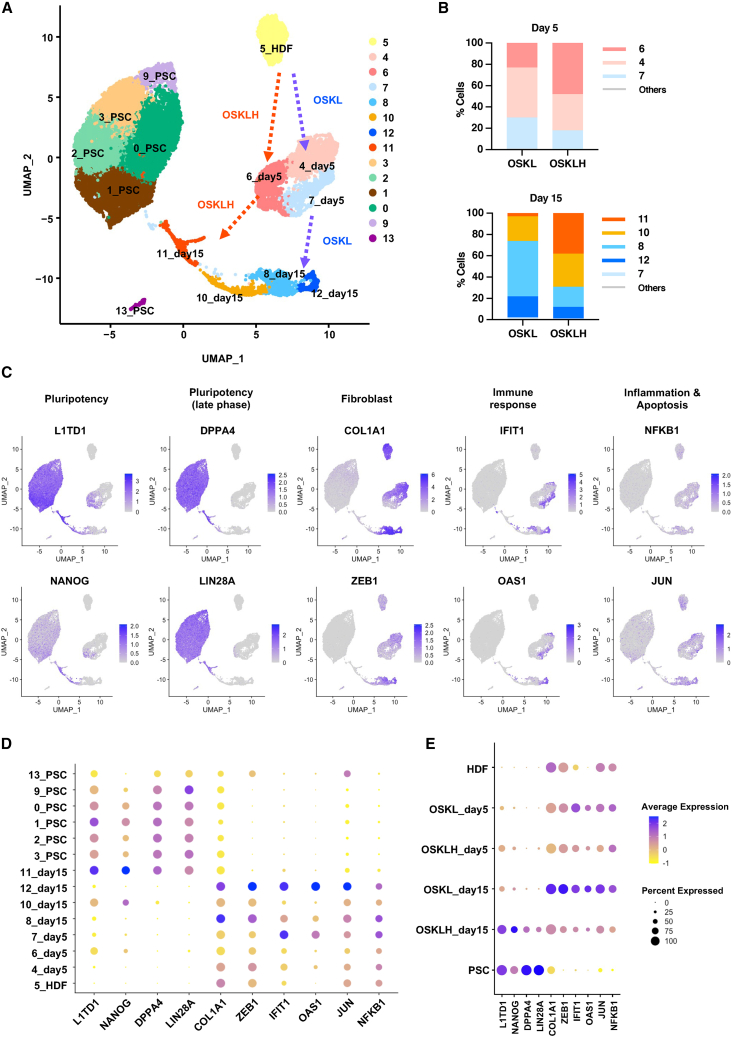
Single-cell RNA-seq analysis of the reprogramming process to iPSCs (A) UMAP of single-cell RNA-seq analysis. Dashed arrows indicate the reprogramming process inferred to be followed by the majority of SeV-OSKL- or SeV-OSKLH-infected HDFs. (B) Percentage of cells in each cluster at day 5 and day 15 after SeV infection. (C) Feature plot of representative marker gene expression in the UMAP. (D) Dot plot of representative marker gene expression by cluster. The color of the dot indicates the average expression of the gene, and the size of the dot indicates the percentage of cells in which the gene is expressed. (E) Dot plot of representative marker gene expression by cell type.

We next investigated the mechanism of OSKLH-mediated improvement of reprogramming using single-cell RNA-seq (scRNA-seq) analysis. We sampled control HDFs, HDFs at day 5 and 15 after infection of SeV-OSKL or SeV-OSKLH, and at early passage (P2) of generated iPSCs. Uniform manifold approximation and projection (UMAP) plots revealed that on day 5, cells in the reprogramming process were classified into three clusters (Figure 2A). On day 15, cells in the reprogramming process were divided into five clusters, and PSCs into five clusters. Comparing the contribution of OSKL- or OSKLH-infected HDFs to each cluster, the highest numbers of OSKL and OSKLH cells on day 5 were in clusters #4 and #6, respectively, and on day 15, the highest numbers of OSKL and OSKLH cells were in clusters #8 and #11, respectively (Figures 2B, and S2A). We then extracted DEGs among these clusters and visualized them in feature plot (Figure 2C), dot plot (Figure 2D), and heatmap (Figure S2B). According to these plots, on day 5, cluster #6 had the highest expression of pluripotency markers and suppressed expression of fibroblast-related genes, innate immune responses associated with general viral infection, and apoptosis markers. Conversely, cluster #7 showed higher expression of immune response and apoptosis markers. On day 15, cluster #8 was similar to cluster #7 in gene expression pattern. Cluster #11 showed not only a high level of pluripotency markers which are expressed in the late phase of reprogramming but also high expression of naive pluripotency, epithelial, and mesoendodermal markers which are transiently upregulated during iPSC generation (Takahashi et al., 2014) (Figure S2C). Next, we detected DEGs among clusters #6 and #7 on day 5 and clusters #11 and #12 on day 15, which are considered to be the cell groups with the most contrasting characteristics, and analyzed their GO terms (Table S1). This showed that the innate immune response, which is associated with viral infection, was suppressed, and cell proliferation was enhanced in cluster #6 compared to cluster #7. In addition, cell proliferation and stem cell maintenance-related genes were upregulated in cluster #11 compared to cluster #12, while the expression of genes related to innate immune response, inflammatory response, and apoptosis was suppressed in cluster #11. It is known that exogenous single-stranded RNA, such as the SeV vector genome used in this study, activates innate immunity in mammalian cells via interferon- and NF-κB-dependent pathways, which can impair reprogramming (Warren et al., 2010). These results suggested clusters #6, #10, and #11 were in the process of successful reprogramming, while clusters #8 and #12 were in the process of incomplete or unsuccessful reprogramming. There were more OSKLH-infected cells belonging to clusters #6, #10, and #11 than there were OSKL-infected cells. Finally, we compared the expression of representative groups of genes characterizing clusters in each cell groups (Figure 2E). Especially on day 15, OSKLH-infected cells showed expression of pluripotency markers more similar to PSCs than OSKL-infected cells, whereas OSKL-infected cells were notable for fibroblast, innate immunity, inflammation, and apoptosis markers. These results indicated that a higher percentage of OSKLH-infected cells were in the process of successful reprogramming.

### OSKLH enhances chromatin opening and transcription factor binding around pluripotency markers

**Figure 3:**
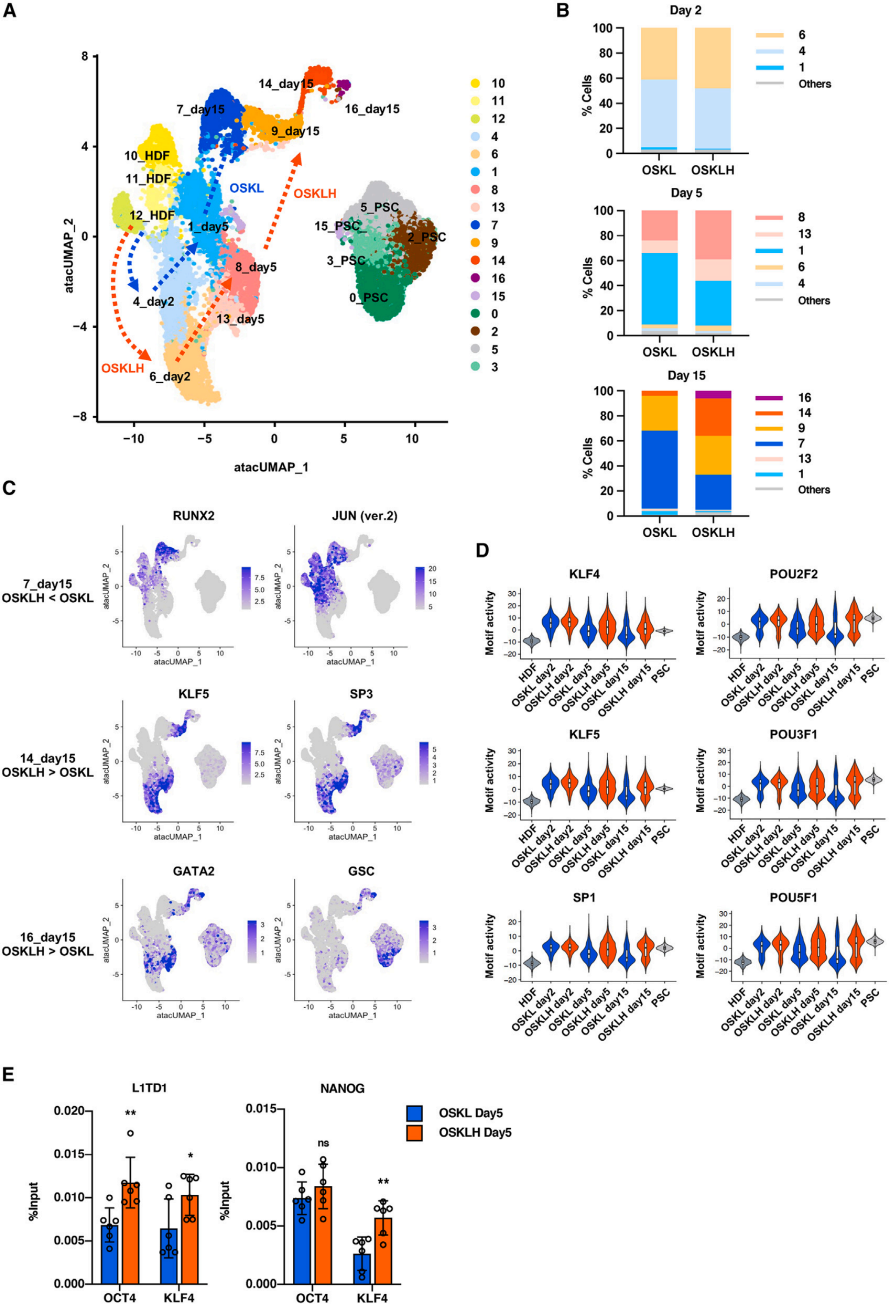
Single-cell ATAC-seq analysis of the reprogramming process to iPSCs (A) UMAP of single-cell ATAC-seq analysis. Dashed arrows indicate the reprogramming process inferred to be followed by the majority of SeV-OSKL- or SeV-OSKLH-infected HDFs. (B) Percentage of cells in each cluster at day 2, day 5, and day 15 after SeV infection. (C) Feature plot of representative motif in #7, #14, and #16 in the UMAP. (D) Violin plot of KLF/SP and POU family motif activity level in each cell group. (E) ChIP-qPCR analysis of *L1TD1* and *NANOG* immunoprecipitated with OCT4 or KLF4. Data are shown as the mean ± SD. *n* = 6. ^∗^*p* < 0.05, ^∗∗^*p* < 0.01.

Linker histone H1 is known as a chromatin remodeling factor, and somatic H1, which exists ubiquitously in the body, functions to aggregate chromatin to form heterochromatin, whereas H1FOO functions to form relatively open chromatin (Ooga et al., 2016; Saeki et al., 2005). Given this role, we hypothesized that the *H1FOO-DD*-mediated increase in reprogramming efficiency may be due to H1FOO selectively opening chromatin regions important for the early stages of reprogramming. To test this hypothesis, we performed single-cell assay for transposase-accessible chromatin using sequencing (scATAC-seq) of HDFs infected with OSKL or OSKLH at day 2, day 5, and day 15, and as well as PSCs (H9 ESC). When we calculated the percentage of reads counted within the detected peak region (peak±500 bp), we observed a clear tendency for PSCs to have a lower ratio of reads within the peak compared to HDFs (Figure S3A). In addition, a comparison of OSKL- and OSKLH-infected cells showed that OSKLH cells had a higher ratio of reads in the peak region similar to PSCs, especially on day 15, implying that many OSKLH-introduced cells were transitioning to a nuclear state similar to that of PSCs. We next projected all the obtained peaks onto the UMAP, which resulted in 16 clusters (Figure 3A). We created plots projecting only OSKL- or OSKLH-infected cells onto UMAP, as well as tabulating the percentage of them belonging to each cluster by post-infection day (Figures 3B and S3B). On day 2, there was no significant difference in the distribution of cells in the clusters between the OSKL and OSKLH groups, but on day 5, most OSKL cells were in cluster #1, whereas most OSKLH cells were in cluster #8, and based on their position on the UMAP and the open region profile of transcription start site, cluster #8 was closer to the PSC clusters than cluster #1. On day 15, the majority of OSKL cells were in cluster #7, whereas OSKLH cells had a particularly large population of cells in clusters #9 and #14, and only OSKLH cells had some population in cluster #16. We then examined the specific characteristics of each cluster by gene activity analysis. Since the gene bodies and promoter regions of expressed genes are open chromatin, we predicted the gene expression activity of the cells using tags in the respective gene regions and 2 kb upstream. Based on this analysis, we extracted cells predicted to have high activity of specific gene expression in the UMAP (Figure S3C). We focused on pluripotency markers present at relatively early stages of reprogramming, such as *L1TD1* and *NANOG*, and found that they were expressed in clusters #4, #6, #8, #9, #13, and #14 and PSCs, while pluripotency markers mainly appearing late in reprogramming, such as *DPPA4* and *LIN28*, were strongly expressed in clusters #14 and #16 and PSCs. In summary, a larger proportion of OSKLH-infected cells changed to a PSC-like chromatin state, and the regions of the pluripotent marker genes were opened earlier in OSKLH-infected cells than in OSKL-infected cells.

In order to determine which transcription factors were activated in *H1FOO-DD*-mediated reprogramming, we performed motif analysis in each cluster based on the extracted peaks. We analyzed and listed top 10 motifs in clusters #1 and #7, which were dominated by OSKL cells, as well as in clusters #8, #14, and #16, which were dominated by OSKLH cells (Table S2). We then visualized the distribution of representative motifs in clusters #7, #14, and #16 with the feature plot (Figure 3C). Comparing clusters #1 and #8, #1 showed motifs of inflammation and apoptosis-related transcription factors at lower levels, while cluster #8 had many *POU* and *KLF* family motifs outside the top 10, in addition to transcription factors related to cell proliferation. In cluster #7, peak regions with *RUNX2/3*, *TEAD1-4*, and *AP-1* motifs were specifically detected, which are known as reprogramming inhibiting factors (Li et al., 2017). On the other hand, cluster #14 had *KLF/SP* family motifs, and cluster #16 had mesoendoderm lineage markers such as *GATA* family and *GSC*. We then extracted motifs that were significantly more abundant in OSKLH than in OSKL cells at each time point (Table S3, and Figure 3D). Interestingly, the *POU* and *KLF/SP* families were highly detected at all time points but especially on day 2, when motif activity of the *POU* family was significantly increased about 3-fold. In addition, the *KLF* family, especially *KLF2*, *4*, and *5*, had significantly higher motif activity at day 5 and day 15. To verify these analyses, we performed ChIP-qPCR of the day 5 HDFs after infection with SeV-OSKL or SeV-OSKLH using *OCT4* and *KLF 4* antibodies to detect *L1TD1* and *NANOG*. The results showed that OSKLH-infected cells had significantly higher levels of *L1TD1* occupying both *OCT4* and *KLF4*, and higher levels of *NANOG* occupying *KLF4* (Figure 3E). These results indicated that *H1FOO-DD* enhanced reprogramming by predominantly increasing the transcriptional activity of the *POU* and *KLF/SP* families from the early stage of reprogramming.

Finally, we integrated the gene activity data from scATAC-seq with the transcriptome data from scRNA-seq to investigate how well the results of the scATAC-seq and scRNA-seq analyses matched (Figure S3D). Interestingly, similar to the UMAP of scRNA-seq, the clusters of HDFs, day 5 cells, day 15 cells, and PSCs were obviously separated. Especially in day 15, cells were clearly distributed in four clusters as in scRNA-seq. Next, we classified the cells analyzed by scATAC-seq with the scRNA-seq-derived clusters and calculated the percentage in each group (Figure S3E). The day 2 OSKLH group had a lower percentage of cells in cluster #5 and a higher percentage of cells belonging to cluster #6, a group with a more suppressed immune response and advanced reprogramming, compared to day 2 OSKL cells of the same time course. This trend was more pronounced when comparing day 5 OSKL and OSKLH cells. At day 15, the OSKLH group accounted for nearly 30% of the most advanced reprogramming cells in cluster #11, while the OSKL group accounted for 4%, a difference of more than 7-fold. This difference was in general consistent with the difference in the number of iPSC colonies in the generation. These results showed that the cluster distribution of scATAC-seq was almost identical to that of scRNA-seq, and that reprogramming was more accelerated in OSKLH-introduced cells in terms of both transcriptome and chromatin accessibility.

### FKBP1A suppresses innate immune responses and promotes reprogramming

**Figure 4:**
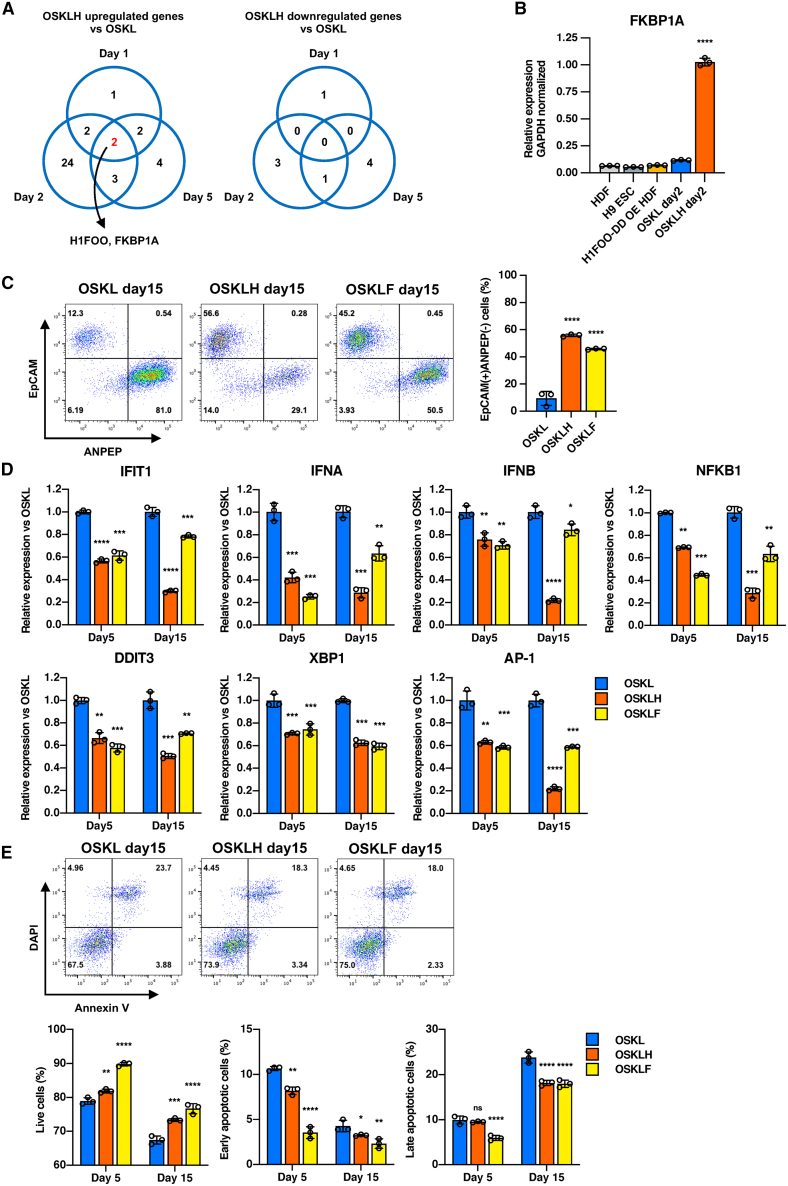
FKBP1A suppresses innate immune responses and promotes reprogramming (A) Upregulated and downregulated DEGs of bulk RNA-seq analysis comparing SeV-OSKL-infected group to SeV-OSKLH-infected group at day 1, 2, and 5. (B) qPCR analysis of FKBP1A expression in PSC and several conditions of HDF. Data are shown as the mean ± SD. *n* = 3. ^∗∗∗∗^*p* < 0.0001. (C) Quantification of EpCAM and ANPEP expression in HDFs during reprogramming at day 15 by flow cytometry. Data are shown as the mean ± SD. *n* = 3. ^∗∗∗∗^*p* < 0.0001. (D) qPCR analysis of innate immune response-related marker gene expression during reprogramming at day 5 and day 15. Data are shown as the mean ± SD. *n* = 3. ^∗^*p* < 0.05, ^∗∗^*p* < 0.01, ^∗∗∗^*p* < 0.001, ^∗∗∗∗^*p* < 0.0001. (E) Quantification of Annexin V and DAPI expression in HDFs during reprogramming at day 5 and day 15 by flow cytometry. Data are shown as the mean ± SD. *n* = 3. ^∗^*p* < 0.05, ^∗∗^*p* < 0.01, ^∗∗∗^*p* < 0.001, ^∗∗∗∗^*p* < 0.0001.

In order to elucidate the mechanism by which *H1FOO-DD* enhances the speed and quality of reprogramming, we attempted to detect DEGs between OSKL- and OSKLH-infected HDFs in the early stages of reprogramming by bulk RNA-seq. We used three types of HDFs infected with OSKL or OSKLH and collected cells on day 1, day 2, and day 5 for bulk RNA-seq. In the day 2 samples, we detected 19 genes which were upregulated and 2 genes which were downregulated in OSKLH compared to OSKL (except *H1FOO*; Figure S4A). Next, to evaluate the role of each of these genes in reprogramming efficiency, we overexpressed them or knocked them down individually in dermal fibroblasts with the PiggyBac system or with shRNA, and then infected with SeV-OSKL to generate primed and naive iPSCs. Overexpression of *FKBP1A* and *APOE* significantly improved both primed and naive reprogramming (Figure S4B). *APOE* has been reported to be highly expressed during the reprogramming process (Tanabe et al., 2013), and *H1FOO* and *FKBP1A* were the two DEGs commonly detected on day 1, day 2, and day 5 in all three types of HDFs (Figure 4A). Based on these results, we hypothesized that *FKBP1A* plays a key role in *H1FOO-DD*-mediated improved reprogramming.

Next, we examined the expression of *FKBP1A* by qPCR at day 2, when SeV-*H1FOO-DD* was introduced alone or together with OSKL to investigate how *FKBP1A* expression changes when *H1FOO-DD* is overexpressed in the beginning of reprogramming. Infection of HDFs with SeV-H1FOO-DD alone did not upregulate *FKBP1A* expression compared to HDFs or PSCs, whereas infection with SeV-OSKL upregulated *FKBP1A* expression about 2-fold. However, *H1FOO-DD* synergized with OSKL to greatly increase *FKBP1A* expression over 8-fold (Figure 4B).

Furthermore, we examined how *FKBP1A* expression changed during reprogramming using the scRNA-seq and the scATAC-seq data previously shown in Figures 2 and 3. We compared the chromatin status of the *FKBP1A* coding region in OSKL- and OSKLH-infected HDFs at day 2 and 5 in the scATAC-seq data and found no obvious differences in chromatin accessibility between the two groups (Figure S4C). Next, we examined the proportion of *FKBP1A*-positive cells and their expression levels in both groups using scRNA-seq data. Interestingly, although the percentage of *FKBP1A*-positive cells was not significantly different between the two groups, OSKLH cells showed markedly higher expression of the gene, especially on day 5 (Figure S4D). In addition, the distribution of cells with high *FKBP1A* expression (Figure S4E) and cells with high *H1FOO* expression (Figure S4F) was almost identical. These results suggest that although the chromatin accessibility of the *FKBP1A* coding region was not significantly different between the OSKL and OSKLH groups, *FKBP1A* transcription was markedly activated in the presence of OSKLH.

FKBP1A is an immunophilin protein known to be involved in immunosuppression, and it binds to TGFBR1 and suppresses its phosphorylation, thereby inhibiting the activity of downstream signaling pathways (Chen et al., 1997; Yamaguchi et al., 2006). Moreover, inhibition of TGFBR1 phosphorylation (pTGFBR1) is known to promote mesenchymal-to-epithelial transition (MET) and improve reprogramming efficiency (Ruetz et al., 2017). We hypothesized that *FKBP1A* plays a central role in the mechanism of the enhanced reprogramming effect of H1FOO-DD by suppressing innate immunity and promoting MET. To test this hypothesis, we used HDFs with overexpression of OSKL and *FKBP1A* (OSKLF). In our examination, this combination of factors achieved the same degree of suppression of pTGFBR1 as OSKLH (Figure S4G). Next, to test whether *FKBP1A* promotes MET during the reprogramming process, we performed flow cytometry of OSKLF-overexpressing HDFs to measure the expression of MET marker EpCAM and the fibroblast marker ANPEP on day 15. Although OSKLF-infected cells did not achieve the level of EpCAM+ANPEP− cells observed in the OSKLH condition, the degree of EpCAM+ANPEP− epithelial transition was much greater than when OSKL was expressed alone (Figure 4C).

To examine whether *FKBP1A* enhances the suppression of innate immune responses during reprogramming, we sampled OSKLF-overexpressing HDFs on day 5 and day 15 to examine their innate immune responses and expression of apoptosis markers. OSKLF and OSKLH significantly suppressed the expression of these markers compared to OSKL (Figure 4D). Finally, we performed an apoptosis assay using AnnexinV to compare the inhibition of apoptosis during the reprogramming process and found that OSKLF inhibited apoptosis as well as OSKLH on both day 5 and day 15 (Figure 4E). These results indicate that the expression of *FKBP1A* is strongly driven by OSKLH, which promotes MET and enhances reprogramming efficiency by suppressing innate immune responses, inflammation, and apoptosis.

### Excessive innate immune response leads to irreversible selection of iPSCs with low differentiation potency

**Figure 5:**
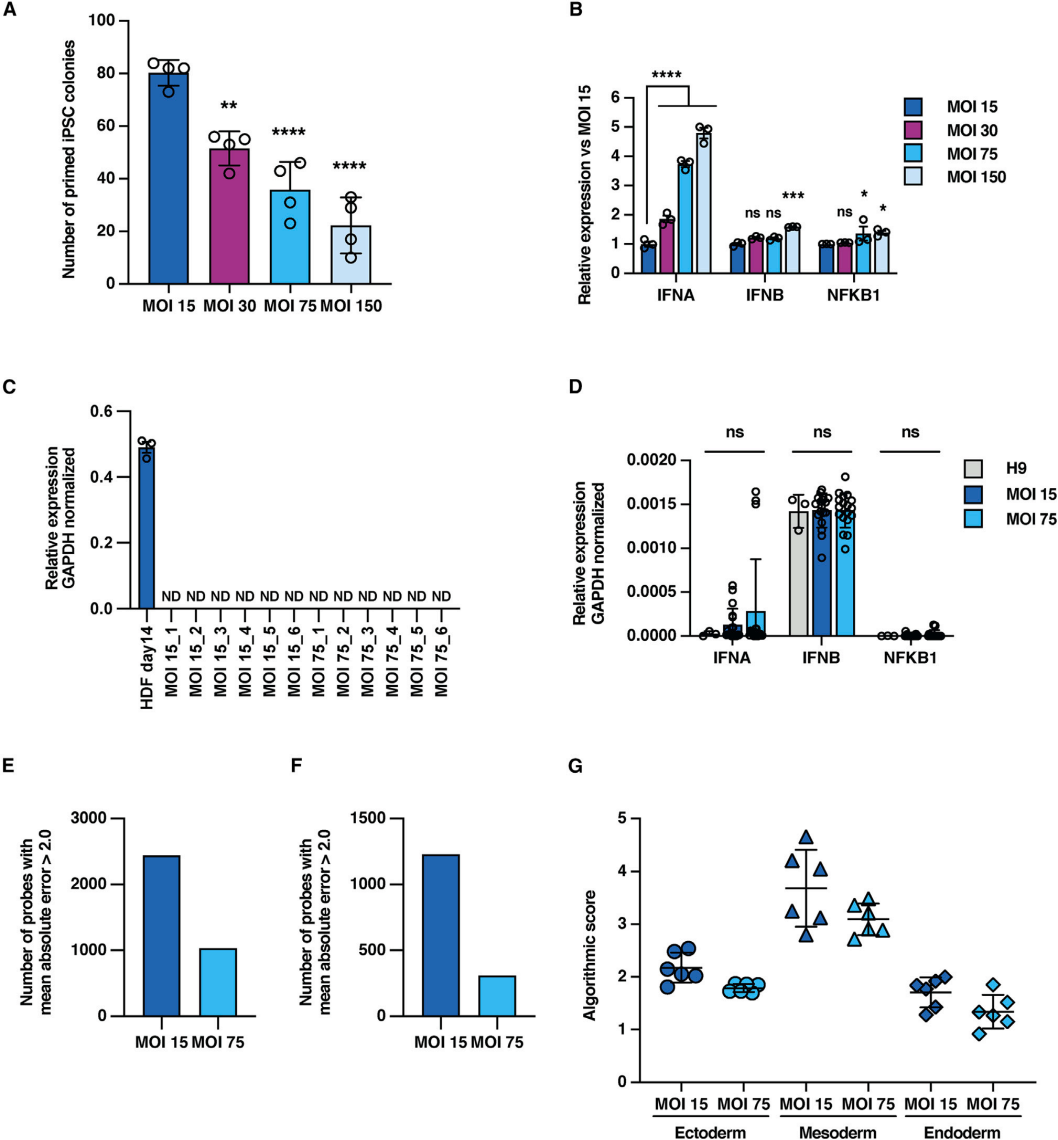
Excessive innate immune response deteriorates reprogramming (A) Number of AP-positive primed human iPSC colonies generated from HDFs at day 14. Each HDF was infected with SeV-Mock in addition to OSKL MOI = 15 except for MOI 15 condition. Data are shown as the mean ± SD. *n* = 4. ^∗∗^*p* < 0.01, ^∗∗∗∗^*p* < 0.0001. (B) qPCR analysis of innate immune response-related inflammation marker gene expression in each condition at day 14. Data are shown as the mean ± SD. *n* = 3. ^∗^*p* < 0.05, ^∗∗∗^*p* < 0.001, ^∗∗∗∗^*p* < 0.0001. (C) qPCR analysis of SeV genome expression in generated iPSC clones at p20 (day 160). Data are shown as the mean ± SD. *n* = 3 for each clone. ND, not detected even after 40 amplification cycles. (D) qPCR analysis of innate immune response-related inflammation marker gene expression in each condition at p20 (day 160). Data are shown as the mean ± SD. *n* = 3 for each clone. (E) Number of genes with MAE >2.0 in clones in the MOI 15 and MOI 75 groups in RNA-seq. (F) Number of genes with MAE >2.0 in clones in the MOI 15 and MOI 75 groups in DNA methylation array. (G) Dot plot of algorithmic scores generated by Scorecard analysis based on 96 genes expression per sample. *n* = 1 of each point.

The activation of the innate immune response we observed in the beginning of reprogramming is considered to be related to viral infection. It has been reported that the transient inflammation caused by overexpression of genes related to innate immune response in iPSCs reduces their differentiation ability and increases heterogeneity among iPSC clones in the short term (Eggenberger et al., 2019). However, the comparison of differentiation potency between OSKL-iPSCs and OSKLH-iPSCs was performed at p20, when the innate immune response is completely suppressed, and we observed that OSKLH-iPSC tended to show better potential to differentiate into some lineages. Therefore, to verify the hypothesis that innate immunosuppression by *H1FOO-DD* and *FKBP1A* contributed to the enhanced differentiation potency of generated iPSCs, we examined the effect of excessive innate immune activation on reprogramming, including in iPSCs that were maintained for many passages. SeV-OSKL is usually used at a total MOI = 15 to generate iPSCs, but to amplify the immune response we infected HDFs not only with OSKL MOI = 15, but also with SeV-Mock MOI = 15, 60, and 135 simultaneously, so that the total MOI = 30, 75, and 150, respectively. Reprogramming efficiency decreased relative to the total MOI (Figure 5A). Comparing the innate immune responses by qPCR showed that the expression of *IFNA* and *NFkB1* increased significantly with increasing MOI (Figure 5B).

When SeV-OSKL and SeV-OSKLH were compared, the difference in their iPSC colony generation efficiency and *IFNA* expression level was generally about 3-fold, similar to the difference between MOI = 15 and MOI = 75. Therefore, we compared the cell characteristics between iPSCs generated by infection at a total MOI = 15 (MOI 15-iPSCs) as a control group and at a total MOI = 75 (MOI 75-iPSCs) as representative of excessive innate immune activation. We established six clones each of MOI 15-iPSCs and MOI 75-iPSCs that were negative for the SeV genome (Figure 5C), and the transcriptome and DNA methylome were compared at p20, a remote stage corresponding to day 160 post SeV infection, when markers related to the innate immune response had converged to the equivalent of H9 ESCs (Figure 5D). Interestingly, MOI 75-iPSCs had about half the number of genes with high variation in expression with MAE>2.0 compared to MOI 15-iPSCs (Figure 5E), and the number of methylated probes meeting the same criteria was reduced to about one-quarter (Figure 5F). Next, we differentiated the iPSCs using the Trilineage Differentiation Kit at p20 and compared the expression levels of the marker genes for each lineage using the Scorecard. MOI 75-iPSCs had markedly lower differentiation ability in all lineages compared to MOI 15-iPSCs, especially in the ectoderm and mesoderm lineages, and showed less variability (Figure 5G). In summary, excessive SeV infection caused a stronger innate immune response which decreased in the efficiency of iPSC generation, but the variation in the transcriptome and DNA methylome of the generated iPSCs was relatively suppressed. Moreover, a marked decrease in differentiation capacity was observed even at the remote stage after SeV infection, when the innate immune response was considered to have ended. These results indicate that the innate immune response to excessive SeV vector in the early stages of reprogramming not only reduces the efficiency of iPSC generation, but also has the effect of irreversible selection of iPSCs with low differentiation potency.

### H1FOO-DD enhances reprogramming into naive pluripotency

**Figure 6:**
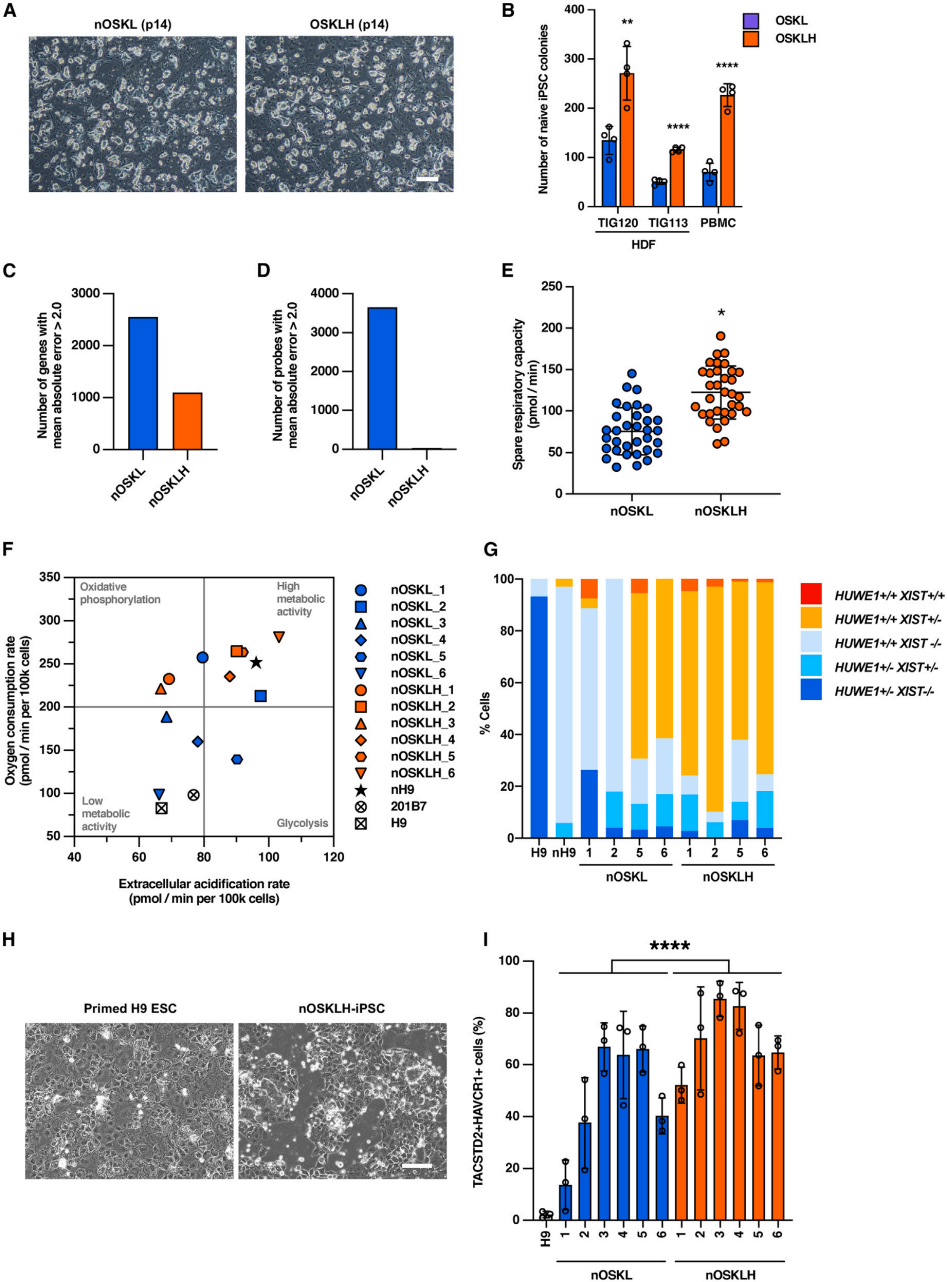
H1FOO-DD enhances reprogramming into naive pluripotency (A) Representative phase-contrast images of naive iPSCs reprogrammed with SeV-OSKL or SeV-OSKLH on iMEF feeder cells (p14). Scale bars, 200 μm. (B) Comparison of AP-positive naive human iPSC colonies generated from HDFs or PBMCs at day 14. Data are shown as the mean ± SD. *n* = 4. ^∗∗^*p* < 0.01, ^∗∗∗∗^*p* < 0.0001. (C) Number of genes with MAE >2.0 in clones of nOSKL-iPSCs and nOSKLH-iPSCs in RNA-seq. (D) Number of genes with MAE >2.0 in clones of nOSKL-iPSCs and nOSKLH-iPSCs in DNA methylation array. (E) Spare respiratory capacity of 6 clones each of nOSKL-iPSCs and nOSKLH-iPSCs analyzed by Seahorse. *n* = 1 of each point and *n* = 6 for each clone. ^∗^*p* < 0.05. (F) Metabolic profiles showing ECAR and OCR under FCCP-induced respiration measured at 50 min in the Seahorse analysis shown in Figure S5E. Each point shows the average of *n* = 6 for each clone. (G) Quantification of the RNA fluorescence *in situ* hybridization patterns for *XIST* with *HUWE1* in cells with bi-allelic *UTX* expression. 100 cells were analyzed in each cell line. (H) Representative phase-contrast images of primed H9 ESC and nOSKLH-iPSC-derived differentiated cells on day 3 after trophectoderm induction. Scale bars, 400 μm. (I) Percentage of TACSTD2+HAVCR1+ cells differentiated from primed H9 ESCs (H9), nOSKL-iPSCs, and nOSKLH-iPSCs analyzed by flow cytometry. Data are shown as the mean ± SD. *n* = 3. ^∗∗∗∗^*p* < 0.0001.

The scRNA-seq and scATAC-seq analyses showed that SeV-OSKLH-infected HDFs transiently had a significantly larger group of cells with naive state marker expression. Therefore, we hypothesized that H1FOO-DD may have a positive effect on reprogramming to the naive state, which requires a higher level of pluripotency. To examine this hypothesis, we generated naive iPSCs from HDFs and PBMCs by infecting with SeV-OSKL (nOSKL-iPSCs) or SeV-OSKLH (nOSKLH-iPSCs) using naive iPSC culture media under on-feeding and hypoxia conditions (Kunitomi et al., 2022). There was no significant difference in the morphology of naive iPSCs generated by infection with either SeV-OSKL or SeV-OSKLH, and the typical dome-like shape of naive PSCs was observed, similar to mouse PSCs (Figure 6A). However, SeV-OSKLH induced significantly more efficient generation of iPSC colonies and regardless of the original cell type, as was the case with primed iPSCs (Figure 6B).

Next, we generated 6 clones each of nOSKL-iPSCs and nOSKLH-iPSCs and compared their transcriptome and methylome by bulk RNA-seq and DNA methylation arrays. As comparison, we included naive PSCs from the datasets of Reset H9 ESC (nH9) (Takashima et al., 2014), which were converted to naive state by overexpression of *KLF2* and *NANOG*, 4i/5i/6iLA naive PSCs (Theunissen et al., 2014), and human blastocyst cells (Yan et al., 2013). In PCA, both nOSKL-iPSCs and nOSKLH-iPSCs were located in almost the same area as nH9 cells, clearly distinguishing them from primed PSCs (Figure S5A). Moreover, there was no significant difference in expression of pluripotency markers between nOSKL-iPSCs and nOSKLH-iPSCs (Figure S5B), and no DEGs were found between nOSKL-iPSCs and nOSKLH-iPSCs. Next, we examined the number of genes that showed MAE >2.0 among the clones. As with the primed iPSCs, nOSKLH-iPSCs had less than half as many genes with MAE >2.0 in expression compared to nOSKL-iPSCs (Figure 6C). DNA methylation analysis showed that all naive PSCs were markedly demethylated compared to primed PSCs, and in PCA, naive PSCs formed a distinct group from primed PSCs generated from the same cells (Figures S5C and S5D). When we examined the number of probes with the MAE >2.0 in DNA methylation in the same manner as in Figure 6C, remarkably, nOSKL-iPSCs had more than double the number of genes with MAE >2 compared to their primed counterparts, whereas variability in methylation in nOSKLH-iPSCs was suppressed to only 29 probes (Figure 6D). In summary, OSKLH-iPSCs showed a more uniform trend in transcriptome and DNA methylation than OSKL-iPSCs, especially in DNA methylation, even in the naive state.

Compared to primed PSCs, naive PSCs are known to have enhanced aerobic and anaerobic metabolism. We compared the expression of oxidative phosphorylation, glycolytic system, and tricarboxylic acid cycle-related genes in nOSKL and nOSKLH using bulk RNA-seq data but found no significant differences between the two groups (Figures S6A–S6C). We further examined the metabolic function of the generated naive iPSCs by monitoring the extracellular acidification rate (ECAR) and oxygen consumption rate (OCR) using a Seahorse flux analyzer (Figure S6D). nOSKLH-iPSCs showed clearly higher spare respiratory capacity than nOSKL-iPSCs, indicating higher aerobic metabolism (Figure 6E). Metabolic states defined by ECAR and OCR showed a trend toward a more energetic phenotype in nOSKLH-iPSCs than in nOSKL-iPSCs, and some nOSKLH-iPSC clones showed profiles similar to or higher than those of nH9 cells (Figure 6F). In summary, nOSKLH-iPSCs were more active in both aerobic metabolism and glycolysis than nOSKL-iPSCs and were reprogrammed to a level similar to more naive ESCs.

In principle, female-derived somatic cells and primed human PSCs have one X chromosome inactivated, but it is known that reprogramming to the naive state results in reactivation of the inactivated allele (Weinberger et al., 2016). The naive iPSCs generated in this study were all female-derived cells, so we performed RNA fluorescence *in situ* hybridization to examine the expression of the X chromosome gene *HUWE1* and the X chromosome inactivation factor *XIST*. To ensure that only normal cells with a diploid X chromosomes were included in the analysis, we only considered cells with biallelic expression of *UTX*, which escapes X chromosome inactivation. As previously reported, primed H9 ESCs were predominantly *HUWE1*^+/−^, and Reset nH9 ESCs were almost all *HUWE1*^+/+^, but few cells expressing *XIST* were observed (Sahakyan et al., 2017). All naive PSCs generated in this study were predominantly *HUWE1*^+/+^ X-active cells. nOSKLH-iPSCs showed a large number of cells with an expression pattern very similar to that of preimplantation blastocysts, such as *HUWE1*^+/+^, *XIST*^+/−^ or *HUWE1*^+/+^, and *XIST*^+/+^ in all clones (Figure 6G). Therefore, nOSKLH-iPSCs more uniformly retained an X chromosome state more similar to preimplantation blastocyst than nH9 ESC or nOSKL-iPSCs.

One of the main unresolved issues facing naive human PSCs is the apparent higher frequency of chromosomal aberrations than in primed PSCs (Theunissen and Jaenisch, 2014). We tested primed PSCs and naive PSCs by karyotyping to see if this problem is improved by H1FOO-DD (Table S4). Most of the primed iPSC clones showed no abnormalities, whereas the naive iPSC clones showed polyploidy in all clones as well as numerous other karyotypic abnormalities in some clones, similar to the Reset H9 ESCs and OSKL-iPSCs. Given the possibility that the t2iLGö+Y medium caused karyotypic aberrations, we also examined genomic aberrations of naive iPSCs generated in PXGLY (Bredenkamp et al., 2019) or AXGY (Khan et al., 2021) media (Table S5). We generated 3 clones of naive iPSCs each in each medium condition. For PXGLY medium, naive iPSCs were generated under the following two conditions: naive iPSCs generated using PXGLY medium only (clone name: PXGLY), and those generated using t2iLGö+Y medium until day 14 from the beginning of reprogramming, followed by using PXGLY medium to maintain the naive iPSCs (clone name: t2iLGö+Y to PXGLY). We were unable to generate naive iPSCs in AXGY alone, so we generated naive iPSCs using t2iLGö+Y or 5iLA for the first 14 days, and then changed the medium to AXGY medium (clone names: t2iLGö+Y→AXGY and 5iLA→AXGY). We then compared CNVs by SNP genotyping array (Figure S6E), and no abnormalities were detected in t2iLGö+Y medium or PXGLY medium. However, when AXGY medium was used, some abnormalities were detected in both nOSKL-iPSCs and nOSKLH-iPSCs, with slightly more abnormalities in the nOSKL-iPSC group (Table S5). Taken together, H1FOO-DD was found to have no apparent effect on suppressing genomic abnormality. In addition, there was no obvious indication that cells grown in t2iLGö+Y medium were more prone to genomic aberrations than those grown in PXGLY or AXGY.

Finally, to investigate the naive PSC-specific differentiation potency, we differentiated nOSKL-iPSCs and nOSKLH-iPSCs into naive-derived trophectoderm (nTE), which is difficult to induce differentiation from primed PSCs (Io et al., 2021). After a 3-day differentiation induction period, naive iPSCs differentiated into cells with morphology identical to previously reported naive PSC-derived differentiated cells, in obvious difference to primed PSCs (Figure 6H). The expression of TACSTD2 and HAVCR1, representative markers of nTE, was also examined using flow cytometry, and we found that there were almost no TACSTD2+HAVCR1+ cells in primed iPSCs, but naive iPSCs were able to differentiate to nTE, with nOSKLH-iPSCs showing a significantly better capacity than nOSKL-iPSCs (Figure 6I).

## Discussion

We found that addition of *H1FOO-DD*, human *H1FOO* with a destabilizing domain, enhanced both primed and naive reprogramming when overexpressed in human somatic cells for a short period with OSKL. *H1FOO-DD* promoted the chromatin accessibility and activity of the *POU* and *KLF/SP* families and suppressed the innate immune response in the early stages of reprogramming. In addition to enhancing reprogramming efficiency, *H1FOO-DD* also made the transcriptome and methylome more uniform among generated iPSC lines, and generated iPSCs with higher potential to differentiate to some lineages. Therefore, the addition of *H1FOO-DD* to iPSC reprogramming protocols is expected to advance research into human early development, disease modeling, drug discovery, and personalized cell therapy efforts.

Reprogramming is a phenomenon that involves drastic modification of the epigenome, so factors that modify the epigenome to recapitulate its early developmental state are very effective in reprogramming (Okita et al., 2007; Papp and Plath, 2013; Takahashi et al., 2007). H1FOO is involved in chromatin decondensation immediately after fertilization in mice, and is known to be rapidly downregulated after fertilization (Funaya et al., 2018; Tanaka et al., 2003). It has been reported to improve the reprogramming efficiency of mouse iPSCs and reduce the heterogeneity of differentiation potency among generated iPSC clones (Kunitomi et al., 2016). Based on these previous findings and our results of this study, we postulate that H1FOO-DD changes the wide range of chromatin in somatic cells to a chromatin more similar to that of the fertilized egg when it achieves totipotency and thereby facilitates faster and more efficient binding of reprogramming factors such as OCT4 and KLF4 to target genes. After reprogramming factors bind to the target regions and reprogramming begins, H1FOO-DD is rapidly degraded and thus does not interfere with the dynamically changing chromatin structure during the reprogramming process.

It has been reported in several previous studies that a strong and sustained innate immune response during the reprogramming process reduces reprogramming efficiency (Diebold et al., 2004; Warren et al., 2010). In support of this, our scRNA-seq and scATAC-seq analyses showed that innate immune responses were strongly persistent in cell clusters where reprogramming was considered unsuccessful. In addition, our study found that iPSCs generated during the process of excessive innate immune response showed more uniform transcriptome, methylome, and inferior differentiation potency even after the innate immune response had disappeared by long-term passaging. These results suggest that excessive innate immune activation in the early stage of reprogramming forced uniformly low-quality reprogramming in SeV-infected cells, and this environment led to the selection of iPSCs with poor differentiation ability that just barely achieved reprogramming to the PSC level. Thus, it is crucial to minimize the innate immune response in the early stages of iPSC generation to ensure their function. *H1FOO-DD* suppressed innate immune responses, and we further showed that this occurred at least in part through upregulation of *FKBP1A*. scATAC-seq results showed that chromatin accessibility around *FKBP1A* was not altered by the presence of *H1FOO-DD*. This suggests that changes in chromatin structure, which is considered to be one of the major functions of H1FOO, may not be directly related to its effect on *FKBP1A* expression. This may also be one of the reasons why high expression of H1FOO-DD alone did not increase *FKBP1A* expression.

Notably, the naive iPSCs we generated in this study showed many karyotypic abnormalities after late passages, and H1FOO-DD did not suppress the formation of karyotypic abnormalities. Since the primed iPSCs did not show such pronounced abnormalities, differences in culture environment and pluripotency state are likely the main causes of karyotypic abnormalities. Further work is necessary to completely solve this problem.

## Experimental procedures

### Resource availability

#### Lead contact

Further information and requests for resources and reagents should be directed to and will be fulfilled by the lead contact, Akira Kunitomi: akira.kunitomi@gladstone.ucsf.edu.

#### Materials availability

All unique/stable reagents generated in this study, including SeV vectors, are available from the lead contact with a completed Materials Transfer Agreement.

#### Data and code availability

• Single-cell RNA-seq, single-cell ATAC-seq, bulk RNA-seq, DNA methylation array, and SNP genotyping array data are accessible in the Gene Expression Omnibus database of the National Center for Biotechnology Information website. The accession number is GSE224850.
• This paper does not report original code.
• Any additional information required to reanalyze the data reported in this paper is available from the lead contact upon request.

### Experimental model and subject details

We obtained HDFs collected under informed consent from the Tokyo Metropolitan Institute of Gerontology (Kondo and Yonezawa, 1995). iMEFs were purchased from Thermo Fisher Scientific and PBMCs were purchased from Cellular Technology Limited. Primed and naive ESC clones were obtained from WiCELL (Thomson et al., 1998) and Kyoto University (Takashima et al., 2014). All cells except naive PSCs were cultured in humidified incubators at 37°C in 5% CO_2_ and 20% O_2_, and naive PSCs were cultured under 5% O_2_ throughout. Recombinant DNA experiments in this study were carried out under the approval of Kyoto University and The J. David Gladstone Institutes.

### Method details

#### Cell culture

HDFs and iMEFs were maintained in Dulbecco’s modified Eagle’s medium (DMEM, Nacalai Tesque) supplemented with 10% fetal bovine serum (FBS, Japan Bio Serum). PBMCs were cultured in StemSpan ACF (STEMCELL) with 100 ng/mL human SCF (R&D), 100 ng/mL human TPO (R&D), 100 ng/mL human Flt3/Flk2 (R&D), 50 ng/mL human IL-6 (R&D), and 20 ng/mL human IL-3 (R&D) for 5 days before the SeV vectors infection. Primed PSCs were maintained in StemFit AK02N medium (Ajinomoto) on laminin 511-E8 fragments (iMatrix-511, Nippi)-coated plates. Naive PSCs were cultured on iMEFs and maintained in t2iLGö (Takashima et al., 2014) medium composed of N2B27 medium (NDiff227, Takara Bio) with 1 μM CHIR99021 (Merck), 1 μM PD0325901 (Merck), 10 μg/mL human LIF (PeproTech), and 2.5 μM Gö6983 (Merck). The day before naive PSCs were plated, iMEF cells were seeded in cell culture dishes at a concentration of 25,000 cells/cm^2^ and cultured overnight. The next day, the cells were washed twice with PBS(−) before plating. Medium was changed every other day and 10 μM Y27632 (Wako) was added just before every medium change. Primed iPSCs were passaged every 6–7 days, and naive iPSCs were every 3–4 days using Accutase (Innovative Cell Technologies).

#### Reprogramming of human somatic cells into primed iPSCs

We used CytoTune-iPS 2.0L or CytoTuneEX-iPS as SeV-OSKL vectors. HDFs were initially maintained in DMEM (Nacalai Tesque) supplemented with 10% FBS. PBMCs were cultured for 5 days in the StemSpan ACF-based medium described in the cell culture section. At day 0, the cells were counted and infected with three SeV vectors: (1) polycistronic *KLF4-OCT4-SOX2*, (2) *LMYC*, and (3) *KLF4* at a MOI of 5, respectively. *H1FOO-DD* was infected at an MOI of 0.3 for HDFs and at an MOI of 1 for PBMCs.

In HDFs, from day 1, the medium was changed every other day. At day 5, the cells were reseeded on laminin 511-E8 fragment-coated plates. The next day the medium was changed to StemFit AK02N medium. When iPSC colonies grew to the point that they were visible to the eye around day 20, the colonies were manually picked and cloned.

In PBMCs, at day 1, the cells were counted and reseeded on laminin 511-E8 fragment-coated plates with StemSpan ACF-based medium. Beginning on day 2 and then every other day, the same amount of StemFit AK02N medium was added as day 1. At day 8, the medium was completely changed to StemFit AK02N medium.

#### Reprogramming of human somatic cells into naive iPSCs

We used CytoTuneEX-iPS as SeV-OSKL vectors for generating naive iPSCs. HDFs and PBMCs were cultured and infected with SeV vectors as in primed iPSC generation. According to the previously reported protocol (Kunitomi et al., 2022), we started incubating cells in a 5% O_2_ hypoxic incubator at 35°C from the time of infection.

In HDFs, we reseeded cells on iMEF feeder cells at day 5. From the next day, medium was changed to t2iLGö + Y27632 medium. Around day 14, we did the first passage of generated naive iPSCs, and the incubation temperature was changed to 38°C to remove the SeV vectors. After confirming removal of the SeV vectors, the temperature was changed to 37°C.

In PBMC reprogramming, we counted and reseeded cells on iMEF feeder cells with StemSpan ACF-based medium at day 1. Beginning on day 2 and then every other day, we gradually shifted the medium to t2iLGö + Y27632 medium as we did for primed iPSC generation.

### Quantification and statistical analysis

All “n” in figure legends indicate the number of independent replicates from separate experiments. Error bars represent mean ± SD. Statistical significance was determined by two-tailed unpaired Student’s t test or one-way ANOVA using Prism software (GraphPad), and it was set at *p* < 0.05 indicated by asterisk.

## Supplemental information

Document S1. Figures S1–S6, Tables S1–S5, and Supplementary experimental procedures

Document S2. Article plus supplemental information
