# Supplementary material for: H1FOO-DD promotes efficiency and uniformity in reprogramming to naive pluripotency
Source: Stem Cell Reports. 2024 May 2;19(5):710–28. doi: 10.1016/j.stemcr.2024.04.005 (PMC11103934; doi:10.1016/j.stemcr.2024.04.005)
Supplement: Document S1. Figures S1–S6, Tables S1–S5, and Supplementary experimental procedures [file mmc1.pdf]

Stem Cell Reports, Volume *19*

Supplemental Information

H1FOO-DD promotes efficiency and uniformity in reprogramming to naive pluripotency

Akira Kunitomi, Ryoko Hirohata, Mitsujiro Osawa, Kaho Washizu, Vanessa Arreola, Norikazu Saiki, Tomoaki M. Kato, Masaki Nomura, Haruko Kunitomi, Tokiko Ohkame, Yusuke Ohkame, Jitsutaro Kawaguchi, Hiroto Hara, Kohji Kusano, Takuya Yamamoto, Yasuhiro Takashima, Shugo Tohyama, Shinsuke Yuasa, Keiichi Fukuda, Naoko Takasu, and Shinya Yamanaka

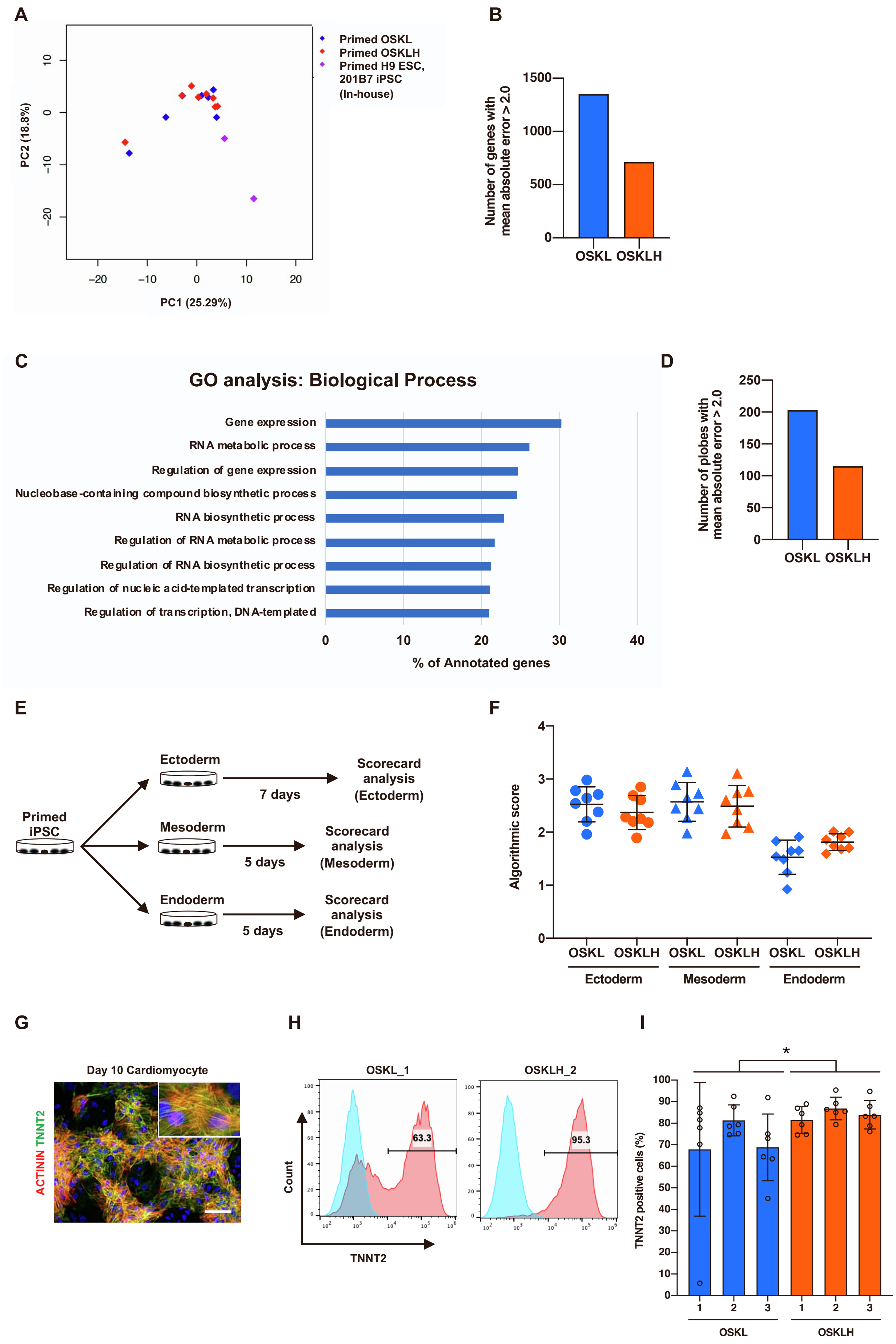

A
Primed OSKL
Primed OSKLH
Primed H9 ESC, 201B7 iPSC (In-house)
PC2 (18.8%)
PC1 (25.29%)
B
Number of genes with mean absolute error > 2.0
OSKL OSKLH
C
GO analysis: Biological Process
Gene expression
RNA metabolic process
Regulation of gene expression
Nucleobase-containing compound biosynthetic process
RNA biosynthetic process
Regulation of RNA metabolic process
Regulation of RNA biosynthetic process
Regulation of nucleic acid-templated transcription
Regulation of transcription, DNA-templated
% of Annotated genes
D
Number of plobes with mean absolute error > 2.0
OSKL OSKLH
E
Primed iPSC
Ectoderm
Mesoderm
Endoderm
7 days
5 days
5 days
Scorecard analysis (Ectoderm)
Scorecard analysis (Mesoderm)
Scorecard analysis (Endoderm)
F
Algorithmic score
OSKL OSKLH OSKL OSKLH OSKL OSKLH
Ectoderm Mesoderm Endoderm
G
Day 10 Cardiomyocyte
ACTININ TNNT2
H
OSKL_1
OSKLH_2
63.3
95.3
Count
TNNT2
I
TNNT2 positive cells (%)
OSKL
OSKLH

Figure S1 related to Figure 1. Characterization of H1FOO-DD vector

(A). PCA of RNA-seq data from primed human PSCs in this study compared to human PSCs.

(B). Number of genes with MAE >2.0 among the clones in each group in RNA-seq.

(C). GO terms for the 1350 genes with MAE >2.0 genes among the OSKL-iPSC clones.

(D). Number of probes with MAE >2.0 among the clones in each group in DNA methylation array.

(E). Schematic representation of the trilineage differentiation and analysis protocol.

(F). Dot plot of algorithmic scores generated by Scorecard analysis based on 96 genes expression per sample. *n*=1 of each point.

(G). Representative immunofluorescent staining for ACTININ and TNNT2 of cardiomyocytes at day 10 post differentiation from OSKLH-iPSCs. Scale bar, 100 μm.

(H). Quantification of TNNT2 expression in primed OSKL-iPSC and OSKLH-iPSC by flow cytometry.

(I). Percentage of TNNT2 expressed cells by flow cytometry. *n*=6 of each clone.

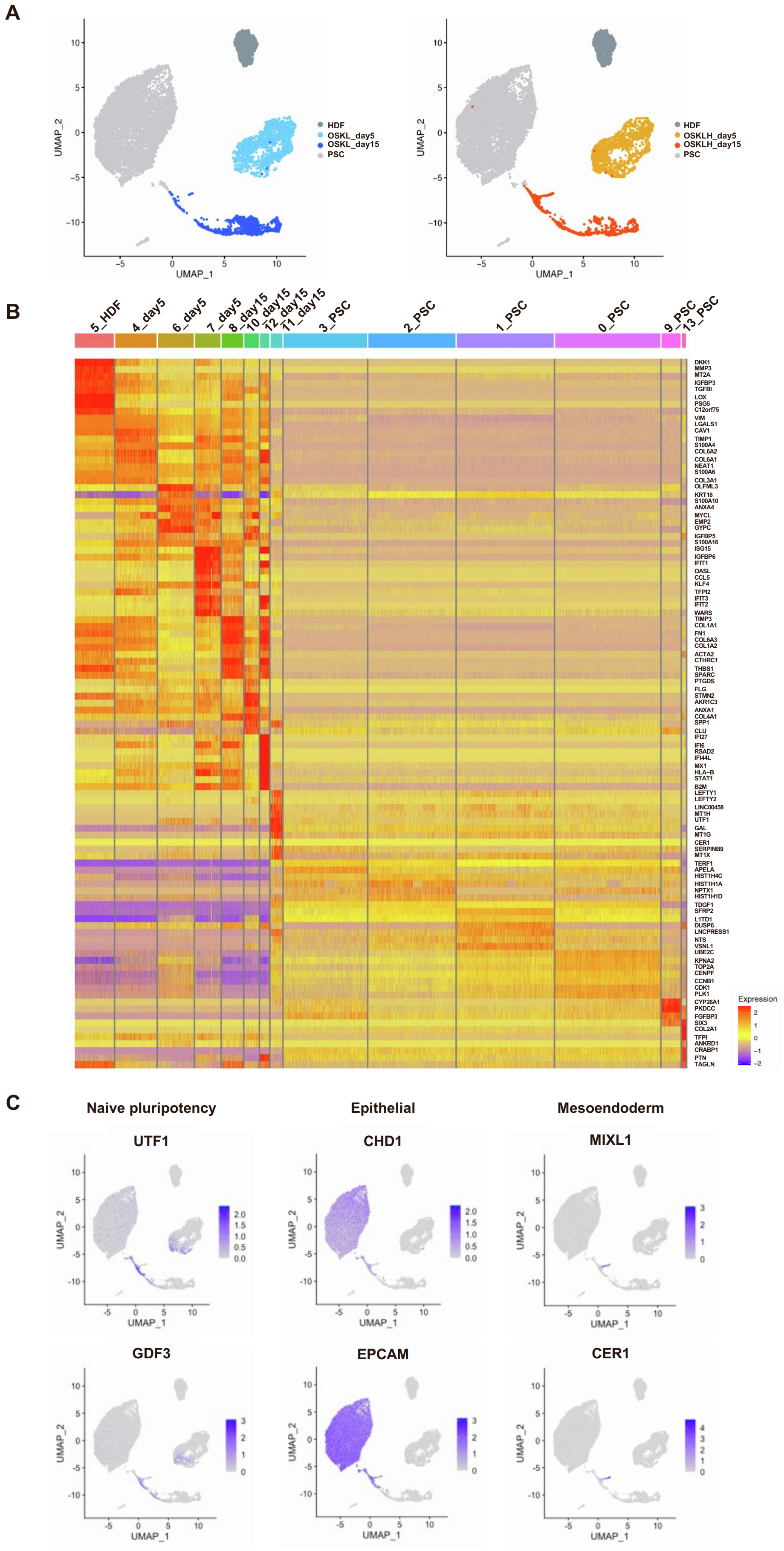
A
HDF
OSKL_day5
OSKL_day15
PSC
HDF
OSKLH_day5
OSKLH_day15
PSC
UMAP_1
UMAP_2
B
5_HDF
4_day5
6_day5
7_day5
8_day15
10_day15
12_day15
11_day15
3_PSC
2_PSC
1_PSC
0_PSC
9_PSC
13_PSC
Expression
C
Naive pluripotency
Epithelial
Mesoendoderm
UTF1
CHD1
MIXL1
GDF3
EPCAM
CER1

Figure S2 related to Figure 2. Specific gene expression in each cell group and clusters in single cell RNA-seq analysis

(A). HDF, PSC and OSKL or HDF, PSC and OSKLH plotted UMAPs for comparing the difference in distribution between OSKL and OSKLH.

(B). Heatmap of top 10 representative marker genes expression in each cluster.

(C). Feature plot of naive pluripotency, epithelial and mesoendoderm related gene expression in the UMAP.

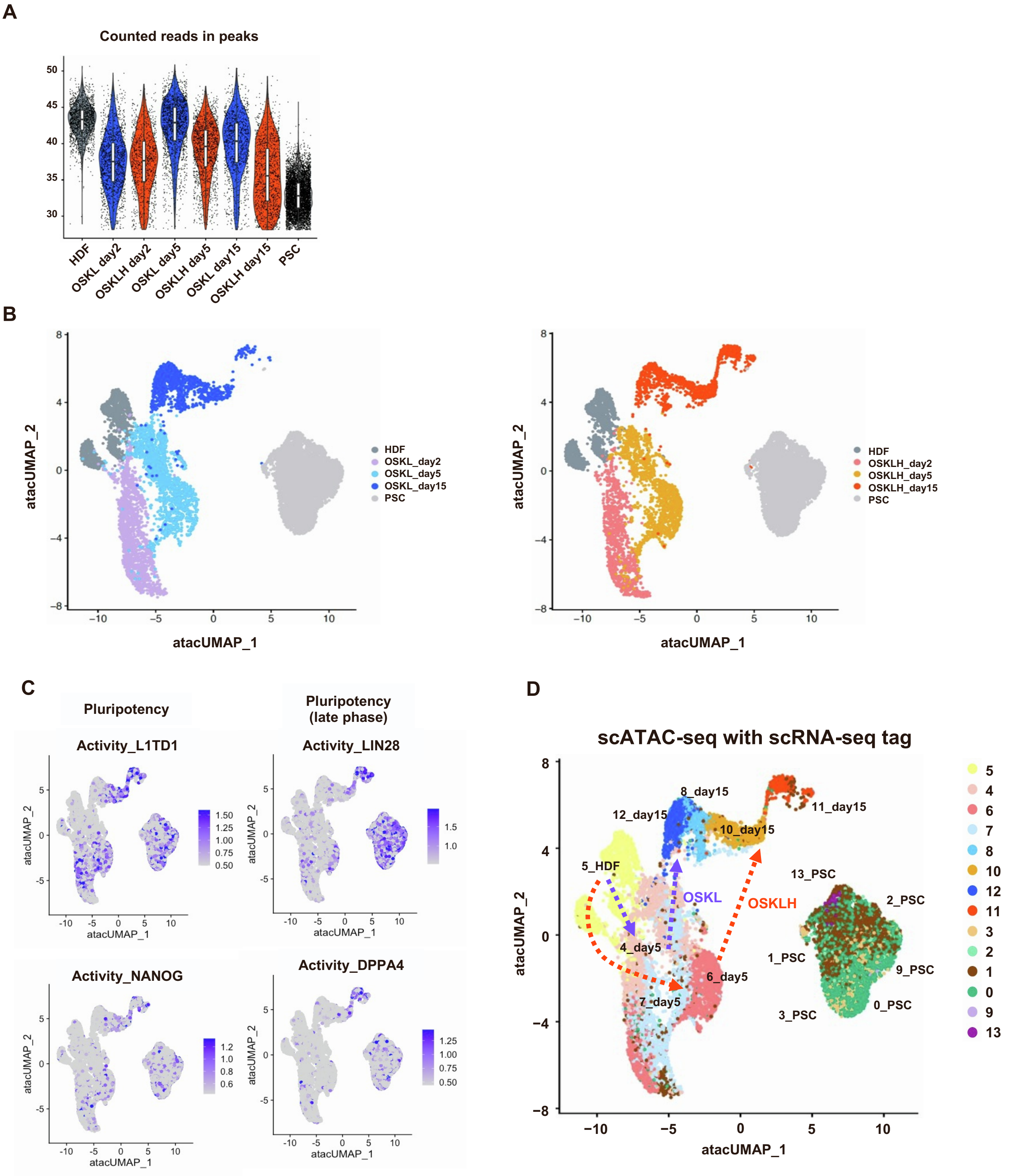

E

scRNA-seq derived clusters

scATAC-seq ID	5_HDF	4_day5	6_day5	7_day5	8_day15	10_day15	12_day15	11_day15	3_PSC	2_PSC	1_PSC	0_PSC	9_PSC	13_PSC	Total (%)
HDF_day0	97.9	1.3	0	0.5	0	0	0	0	0	0	0.3	0	0	0	100
OSKL_day2	8.4	26.9	16.1	34.3	0	0	0	0.2	0.4	0.4	8.9	4.4	0	0	100
OSKLH_day2	3.9	25.2	24.4	32.8	0	0	0	0	0.9	0.4	8.7	3.7	0	0	100
OSKL_day5	3.9	32.7	24.2	31.5	0.4	0	1.0	0	0.7	0	4.4	1.2	0	0	100
OSKLH_day5	0.3	28.4	46.5	19.7	0	0.1	0.5	0	0.4	0	3.5	0.8	0	0	100
OSKL_day15	0.9	3.5	0.3	2.7	26.5	24.9	34.7	4.0	0.2	0	1.9	0.3	0	0	100
OSKLH_day15	0.2	0.8	0.5	1.4	13.2	28.7	15.5	29.6	0.2	0	8.9	1.2	0	0	100
H9_ESC	0	0	0	0	0	0	0	0	12.1	6.7	31.3	45.7	1.5	2.7	100

Figure S3 related to Figure 3. Specific gene expression in each cell group and clusters in single cell RNA-seq analysis

(A). Percentage of reads counted within the detected peak region (peak±500 bp) in HDF, SeV-OSKL or SeV-OSKLH infected HDF and PSC.

(B). HDF, PSC and OSKL or HDF, PSC and OSKLH plotted UMAPs for comparing the difference in distribution between OSKL and OSKLH.

(C). Gene activity plot of pluripotency markers *L1TD1*, *NANOG*, *LIN28* and *DPPA4*. *LIN28* and *DPPA4* are known to be expressed from the late reprogramming stage.

(D). Plot of clusters produced by single cell RNA-seq on the single cell ATAC-seq UMAP by matching the gene activity data of single cell ATAC-seq and the gene expression data of single cell RNA-seq. Dashed arrows indicate the reprogramming process inferred to be followed by the majority of SeV-OSKL or SeV-OSKLH-infected HDFs.

(E). Percentage of cells in each cell group in single cell ATAC-seq that matched the gene expression patterns of the clusters created by single cell RNA-seq analysis.

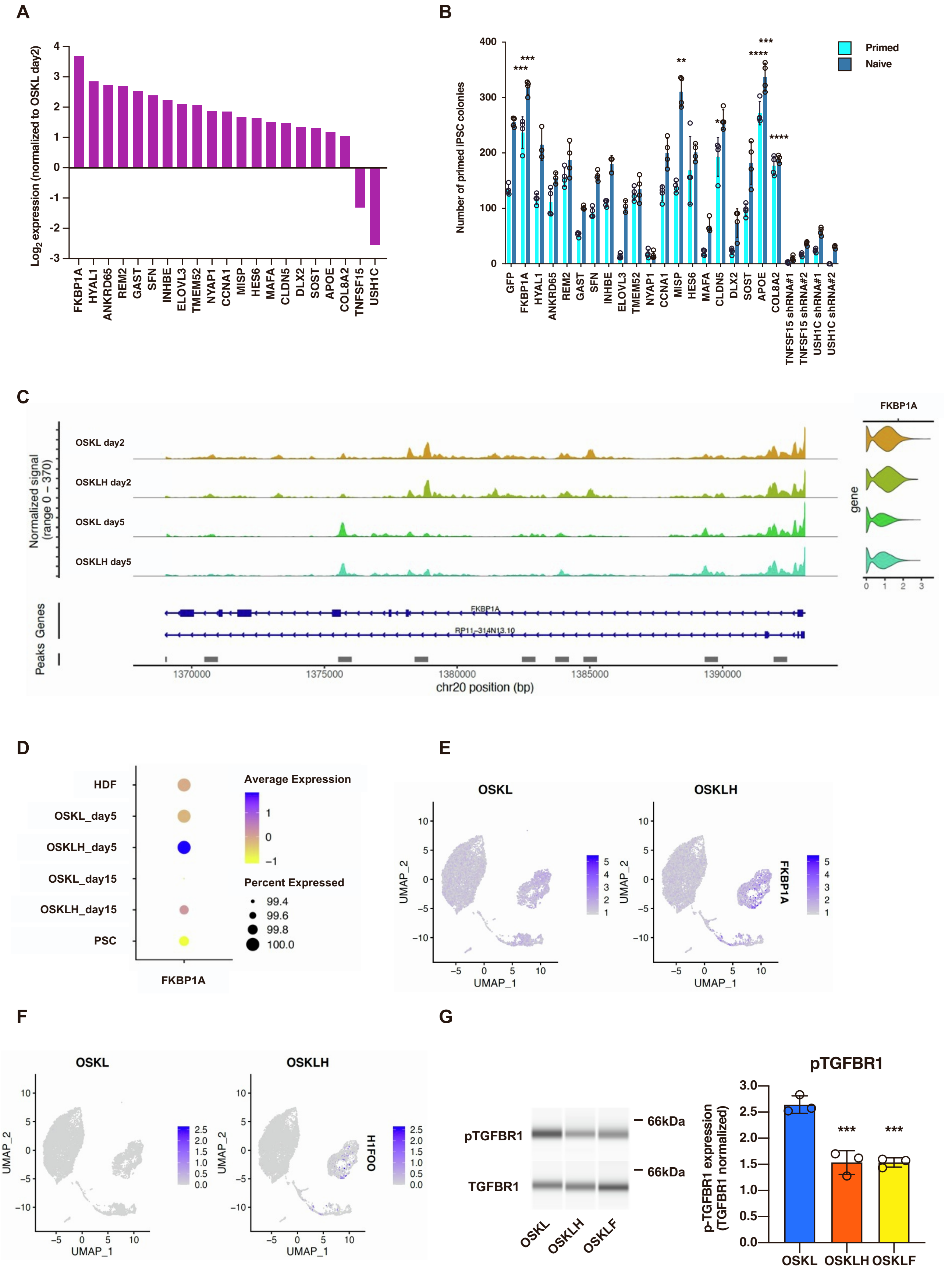
A
Log2 expression (normalized to OSKL day2)
FKBP1A
HYAL1
ANKRD65
REM2
GAST
SFN
INHBE
ELOVL3
TMEM52
NYAP1
CCNA1
MISP
HES6
MAFA
CLDN5
DLX2
SOST
APOE
COL8A2
TNFSF15
USH1C
B
Number of primed iPSC colonies
Primed
Naive
GFP
TNFSF15 shRNA#1
TNFSF15 shRNA#2
USH1C shRNA#1
USH1C shRNA#2
C
Normalized signal (range 0 – 370)
OSKL day2
OSKLH day2
OSKL day5
OSKLH day5
FKBP1A
gene
Peaks Genes
RP11-314N13.10
chr20 position (bp)
D
HDF
OSKL_day5
OSKLH_day5
OSKL_day15
OSKLH_day15
PSC
Average Expression
Percent Expressed
99.4
99.6
99.8
100.0
E
OSKL
OSKLH
UMAP_1
UMAP_2
F
H1FOO
G
pTGFBR1
TGFBR1
66kDa
OSKL
OSKLH
OSKLF
p-TGFBR1 expression (TGFBR1 normalized)

Figure S4 related to Figure 4. Exploration of factors that play an important role in modified reprogramming by H1FOO-DD

(A). Representative DEGs detected in the bulk RNA-seq analysis.

(B). Number of primed and naive human iPSC colonies generated from HDFs at day 14. Data are shown as the mean ± s.d. n=3. *P<0.05.

(C). Coverage plot of *FKBP1A* coding region in OSKL and OSKLH at day 2 and day 5 obtained by single cell ATAC-seq.

(D). Dot plot of *FKBP1A* expression in OSKL and OSKLH in the UMAP obtained by single cell RNA-seq.

(E). Feature plot of *FKBP1A* expression in OSKL and OSKLH in the UMAP obtained by single cell RNA-seq.

(F). Feature plot of *H1FOO* expression in OSKL and OSKLH in the UMAP obtained by single cell RNA-seq.

(G). Protein expression analysis of phosphorylated TGFBR1 (pTGFBR1) and TGFBR1by Western blotting. We quantified the expression level of pTGFBR1 with TGFBR1 protein expression. Data are shown as the mean ± s.d. n=3. ***P<0.001.

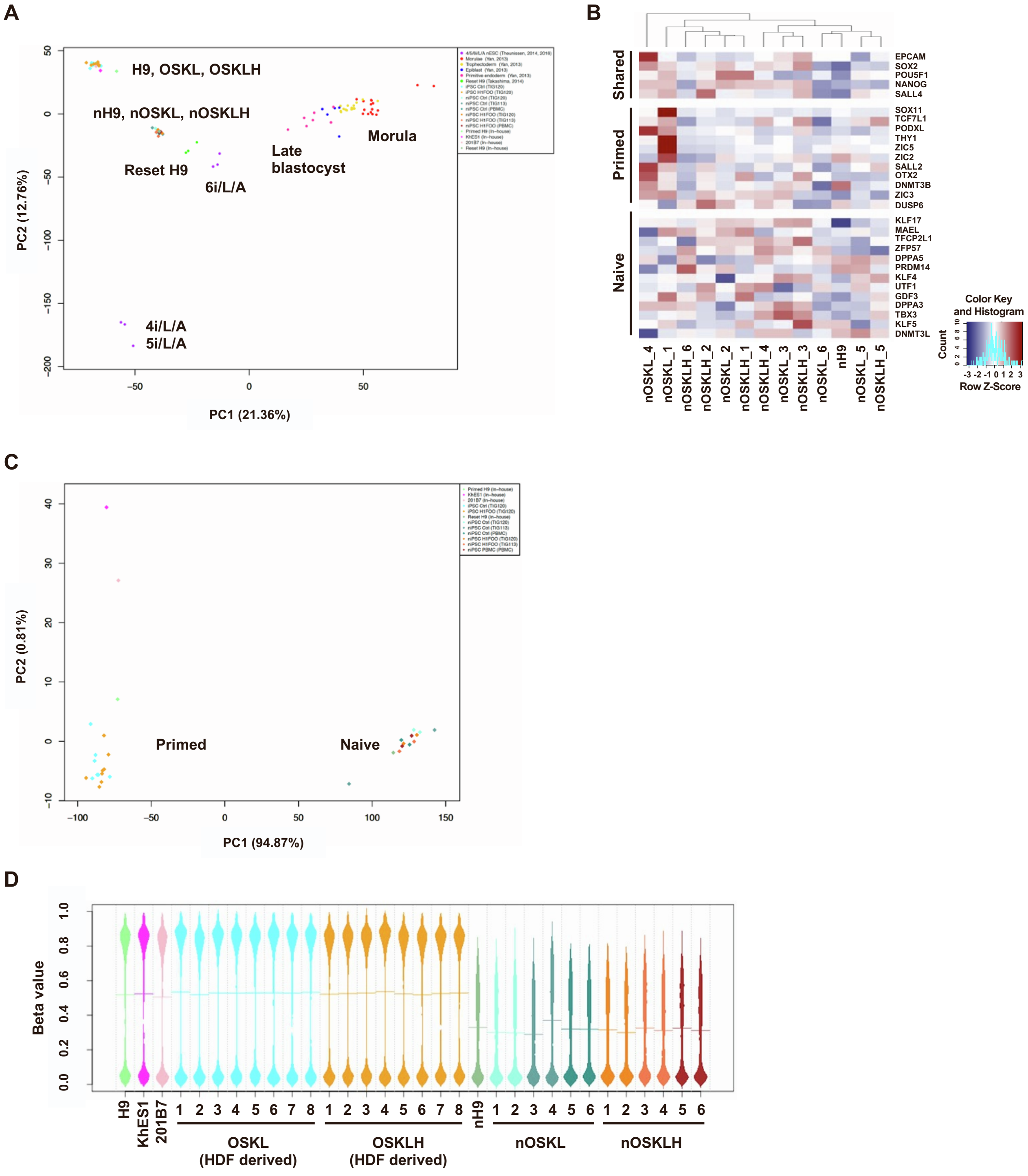

Figure S5 related to Figure 6. Exploration of factors that play an important role in modified reprogramming by H1FOO-DD

(A). PCA of RNA-seq data from primed and naive human PSCs in this study compared to reset naive human iPSCs and pre-implantation embryo samples from (Takashima et al., 2014; Theunissen et al., 2014; Yan et al., 2013).

(B) Heatmap of the RNA-seq data depicting expression levels of shared, primed, and naive

pluripotency-associated marker genes in naive PSCs.

(C). PCA of DNA methylation data from primed and naive human PSCs in this study.

(D). Beanplot of the global DNA methylation levels in primed and naive human PSCs analyzed using DNA methylation arrays. Horizontal lines in the beanplot represent mean methylation beta values.

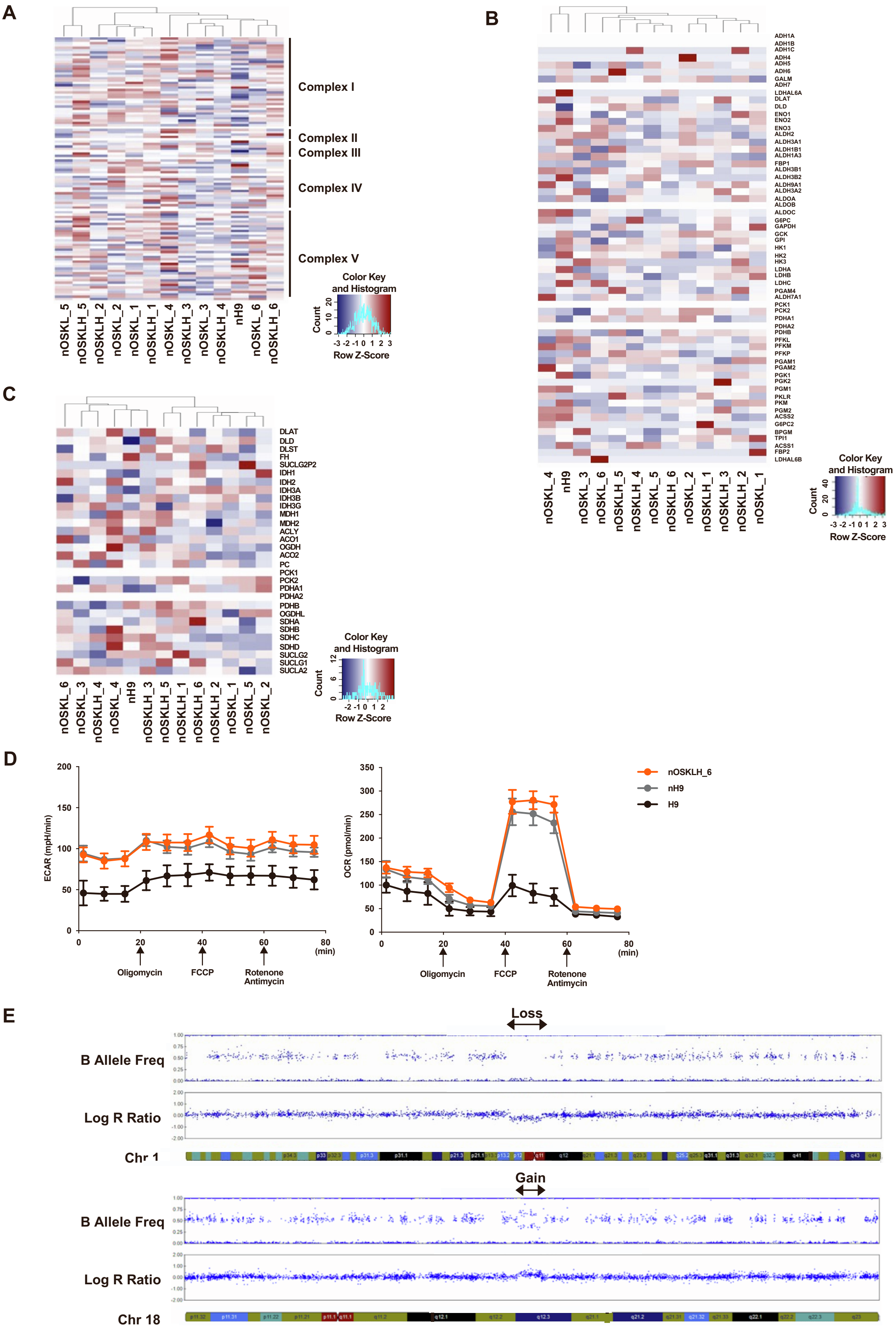
A
Complex I
Complex II
Complex III
Complex IV
Complex V
Color Key and Histogram
Count
Row Z-Score
nOSKL_5
nOSKLH_5
nOSKLH_2
nOSKL_2
nOSKL_1
nOSKLH_1
nOSKL_4
nOSKLH_3
nOSKL_3
nOSKLH_4
nH9
nOSKL_6
nOSKLH_6
B
nOSKL_4
nH9
nOSKL_3
nOSKL_6
nOSKLH_5
nOSKLH_4
nOSKL_5
nOSKLH_6
nOSKL_2
nOSKLH_1
nOSKLH_3
nOSKLH_2
nOSKL_1
Color Key and Histogram
Count
Row Z-Score
C
DLAT
DLD
DLST
FH
SUCLG2P2
IDH1
IDH2
IDH3A
IDH3B
IDH3G
MDH1
MDH2
ACLY
ACO1
OGDH
ACO2
PC
PCK1
PCK2
PDHA1
PDHA2
PDHB
OGDHL
SDHA
SDHB
SDHC
SDHD
SUCLG2
SUCLG1
SUCLA2
nOSKL_6
nOSKL_3
nOSKLH_4
nOSKL_4
nH9
nOSKLH_3
nOSKLH_5
nOSKLH_1
nOSKLH_6
nOSKLH_2
nOSKL_1
nOSKL_5
nOSKL_2
Color Key and Histogram
Count
Row Z-Score
D
ECAR (mpH/min)
OCR (pmol/min)
(min)
Oligomycin
FCCP
Rotenone Antimycin
nOSKLH_6
nH9
H9
E
Loss
Gain
B Allele Freq
Log R Ratio
Chr 1
Chr 18

Figure S6 related to Figure 6. Examining the metabolic function and genomic aberrations of generated naive iPSCs

(A) Heatmap of genes encoding proteins of the electron transport chain located in the inner membrane of mitochondria. These genes reflect the activity of oxidative phosphorylation.

(B) Heatmap of genes encoding proteins of glycolytic system in naive PSCs.

(C) Heatmap of genes encoding proteins of TCA cycle in naive PSCs.

(D). Extracellular acidification rate (ECAR) and oxygen consumption rate (OCR) profiles measured by Seahorse of indicated cell lines. Oligomycin, FCCP and rotenone/antimycin were injected at indicated time points to evaluate mitochondrial capacity. n=6 for each point.

(E) Representative CNVs found in generated naive iPSCs: loss in Chr 1, and gain in Chr 18.

Table S1. GO terms and key genes of DEGs when comparing cluster #6 vs. #7 on day 5 and cluster #11 vs. #12 on day 15

	GO terms & Key Genes					
	down-regulated			up-regulated		
	GO terms	FDR	Key genes	GO terms	FDR	Key genes
#6 vs. #7	～type I interferon signaling pathway	1.155E-17	*IFITM3, HLA-C, ISG15, IFIT1, OASL, IFI27*	～cell division	6.64E-06	*CENPV, UBE2C, TUBB, SMC1A, SMCA*
	～defense response to virus	1.787E-09	*RSAD2, ZC3HAV1, DDX60, IFIT2, HERC5*	～cell proliferation	3.26E-04	*CDV3, MCM7, CD70, PRKDC, TNFSF9*
	～extracellular matrix organization	1.748E-06	*FGF2, FN1, COL6A2, COL8A1, FBN1*	～nucleosome assembly	1.801.E-03	*NPM1, NASP, NAP1L1, ANP32B, HIST1H4C*
	～Immune response	2.824E-06	*IFI6, HLA-C, CCL5, CCL2, B2M*	～cell-cell adhesion	8.802.E-03	*KRT18, RANBP1, CAST, CCNB1, FSCN1*
	～negative regulation of proliferation	1.303E-03	*MEG3, CXCL8, WARS, RARRES3, SOD2*	～mitotic nuclear division	1.945.E-02	*CENPF, CDC20, ASPM, CCNB2, BIRC5*
#11 vs. #12	～type I interferon signaling pathway	9.936E-26	*IFIT1, IFIT3, STAT1, HLA-B, OAS1, ISG20*	～translational initiation	1.950E-81	*RPSA, RPS3, RPS3a, RPL6, RPS25*
	～collagen catabolic process	1.020E-14	*COL1A1, COL1A2, CTSL, MMP1, MMP2*	～cell proliferation	2.504E-06	*MCM7, CKS1B, FRAT2, PRDX1, NANOG*
	～positive regulation of I-kappaB kinase/ NF-kappaB signaling	2.960E-06	*LGALS1, BST2, ECM1, SLC20A1, TRIM38*	～somatic stem cell population maintenance	4.056E-04	*KLF4, POU5F1, SOX2, DPPA4, SALL4*
	～positive regulation of fibroblast proliferation	1.276E-05	*JUN, ANXA2, WNT5A, FN1, S100A6*	～stem cell population maintenance	5.755E-03	*LIN28A, DPPA2, TET1, NANOG NODAL*
	～apoptotic process	1.775E-03	*SH3KBP1, PTEN, COMP, MYDGF, TNFSF10*	～negative regulation of apoptotic process	2.705E-02	*HDSPD1, EPCAM, KRT18, TDGF1, NPM1*

Table S2. Top 10 motifs in clusters #1, #7, #8, #14, and #16 based on the extracted peaks

1_day5
OSKLH < OSKL

motif	observed	background	percent.observed	percent.background	fold.enrichment	pvalue	motif.name
MA1123.2	352	23103	33.05164319	9.998009313	3.305822405	5.43E-94	**TWIST1**
MA1638.1	308	22443	28.92018779	9.712388998	2.977659544	6.28E-70	**HAND2**
MA0698.1	318	25652	29.85915493	11.10110959	2.689745082	2.70E-62	**ZBTB18**
MA0091.1	229	16105	21.50234742	6.969568454	3.0851763	7.72E-53	**TAL1::TCF3**
MA1468.1	159	10064	14.92957746	4.355277052	3.427928301	2.55E-41	**ATOH7**
MA1642.1	269	25244	25.25821596	10.92454431	2.312061287	1.42E-39	**NEUROG2(var.2)**
MA0835.2	335	36828	31.45539906	15.9376136	1.97365803	1.74E-36	**BATF3**
MA1634.1	341	38007	32.01877934	16.44783534	1.94668652	4.57E-36	**BATF**
MA0462.2	341	38036	32.01877934	16.46038533	1.945202297	5.33E-36	**BATF::JUN**
MA0490.2	292	30139	27.41784038	13.04289498	2.102128432	8.04E-36	**JUNB**

8_day5
OSKLH > OSKL

motif	observed	background	percent.observed	percent.background	fold.enrichment	pvalue	motif.name
MA1123.2	850	23103	24.63768116	9.998009313	2.464258673	1.51E-137	**TWIST1**
MA1638.1	795	22443	23.04347826	9.712388998	2.37258601	9.91E-119	**HAND2**
MA0698.1	802	25652	23.24637681	11.10110959	2.094058852	1.72E-92	**ZBTB18**
MA0091.1	579	16105	16.7826087	6.969568454	2.40798391	5.96E-86	**TAL1::TCF3**
MA1640.1	526	14850	15.24637681	6.426457096	2.372438901	2.35E-75	**MEIS2(var.2)**
MA0761.2	926	35294	26.84057971	15.27376274	1.757299767	2.96E-69	**ETV1**
MA1642.1	705	25244	20.43478261	10.92454431	1.870538673	2.05E-60	**NEUROG2(var.2)**
MA0062.3	902	36146	26.14492754	15.64247261	1.671406318	2.94E-57	**GABPA**
MA1639.1	423	12466	12.26086957	5.394761897	2.272735998	5.22E-55	**MEIS1(var.2)**
MA1113.2	466	14433	13.50724638	6.245996988	2.162544491	7.72E-55	**PBX2**

7_day15
OSKLH < OSKL

motif	observed	background	percent.observed	percent.background	fold.enrichment	pvalue	motif.name
MA0511.2	1005	12495	13.5207857	5.40731188	2.50046344	4.48E-159	**RUNX2**
MA0684.2	1114	17773	14.9872192	7.69140889	1.94856617	3.47E-103	**RUNX3**
MA1601.1	1442	28149	19.3999731	12.1817065	1.59254971	3.60E-73	**ZNF75D**
MA0809.2	1420	28103	19.1039957	12.1617996	1.57081981	3.32E-68	**TEAD4**
MA0808.1	1315	25847	17.6913763	11.1854974	1.58163519	2.95E-64	**TEAD3**
MA1121.1	1249	25009	16.8034441	10.8228462	1.55259013	1.97E-56	**TEAD2**
MA0090.3	1319	26825	17.7451904	11.6087348	1.52860675	2.97E-56	**TEAD1**
MA1141.1	1629	35543	21.915781	15.3815195	1.42481248	6.76E-52	**FOS::JUND**
MA0489.1	1652	36625	22.2252119	15.8497637	1.40224247	1.32E-48	**JUN(var.2)**
MA0462.2	1702	38036	22.8978878	16.4603853	1.39109063	3.06E-48	**BATF::JUN**

14_day15
OSKLH > OSKL

motif	observed	background	percent.observed	percent.background	fold.enrichment	pvalue	motif.name
MA0599.1	2324	65239	49.5416755	28.2327027	1.75476206	1.31E-212	**KLF5**
MA0746.2	1486	36653	31.6776807	15.8618809	1.99709485	6.06E-163	**SP3**
MA0039.4	2308	70417	49.2005969	30.4735239	1.61453585	2.71E-161	**KLF4**
MA0741.1	1668	43918	35.5574504	19.0058682	1.87086694	8.13E-160	**KLF16**
MA0079.4	1349	33768	28.7571946	14.613374	1.96786825	1.55E-139	**SP1**
MA1653.1	2004	60460	42.7201023	26.1645519	1.63274733	8.57E-136	**ZNF148**
MA0685.1	1143	27974	24.3658069	12.1059738	2.01270937	4.26E-121	**SP4**
MA1564.1	1252	32748	26.6894052	14.1719607	1.88325425	8.03E-114	**SP9**
MA1515.1	1516	43246	32.3172032	18.7150548	1.72680249	4.54E-112	**KLF2**
MA0747.1	1301	35711	27.7339586	15.4542229	1.79458773	3.92E-104	**SP8**

16_day15
OSKLH > OSKL

motif	observed	background	percent.observed	percent.background	fold.enrichment	pvalue	motif.name
MA0036.3	550	12124	25.9067358	5.24675864	4.93766486	1.77E-219	**GATA2**
MA0482.2	568	13506	26.7545926	5.84483027	4.57747981	2.01E-211	**GATA4**
MA0037.3	453	9106	21.3377296	3.94069484	5.41471251	3.48E-193	**GATA3**
MA0766.2	426	10235	20.0659444	4.42927868	4.53029621	1.26E-152	**GATA5**
MA1104.2	482	13632	22.7037211	5.89935779	3.84850724	2.07E-146	**GATA6**
MA0140.2	307	11642	14.4606689	5.03816926	2.87022292	3.93E-61	**GATA1::TAL1**
MA0648.1	209	7934	9.84455959	3.4335024	2.86720627	3.95E-41	**GSC**
MA0891.1	195	7617	9.1851154	3.29631809	2.78647726	1.02E-36	**GSC2**
MA0035.4	215	9094	10.1271785	3.93550174	2.57328778	1.48E-35	**GATA1**
MA0682.2	264	12519	12.4352332	5.41769807	2.2952983	1.62E-35	**PITX1**

Table S3. Top 10 motifs significantly higher for OSKLH than OSKL at day 2, 5 and 15

	motif	observed	background	percent.observed	percent.background	fold.enrichment	pvalue	motif.name
**Day 2 OSKLH > OSKL**	MA0507.1	120	27677	38.70967742	11.97744465	3.231881136	2.78E-33	**POU2F2**
	MA0627.2	110	26653	35.48387097	11.5343004	3.076378257	3.00E-28	**POU2F3**
	MA0788.1	100	23878	32.25806452	10.3333968	3.121729004	7.76E-26	**POU3F3**
	MA1115.1	103	27430	33.22580645	11.87055341	2.799010737	4.81E-23	**POU5F1**
	MA0786.1	96	25541	30.96774194	11.05307345	2.801731309	2.49E-21	**POU3F1**
	MA0787.1	93	24571	30	10.63329814	2.821325953	8.39E-21	**POU3F2**
	MA0784.1	92	24581	29.67741935	10.63762572	2.78985369	3.12E-20	**POU1F1**
	MA0792.1	81	22166	26.12903226	9.592515017	2.723897978	4.73E-17	**POU5F1B**
	MA0785.1	81	22671	26.12903226	9.811057834	2.663222733	1.71E-16	**POU2F1**
	MA0789.1	75	20487	24.19354839	8.865914245	2.72882725	8.85E-16	**POU3F4**

	motif	observed	background	percent.observed	percent.background	fold.enrichment	pvalue	motif.name
**Day 5 OSKLH > OSKL**	MA0039.4	307	70417	55.01792115	30.47352386	1.80543351	2.16E-33	**KLF4**
	MA0599.1	292	65239	52.3297491	28.23270266	1.853515398	4.40E-33	**KLF5**
	MA1515.1	220	43246	39.4265233	18.71505479	2.106674212	4.21E-30	**KLF2**
	MA0079.4	178	33768	31.89964158	14.61337395	2.182907361	4.64E-25	**SP1**
	MA1511.1	184	36701	32.97491039	15.88265333	2.076158795	1.65E-23	**KLF10**
	MA1107.2	223	53149	39.96415771	23.00065779	1.737522382	2.79E-19	**KLF9**
	MA0761.2	169	35294	30.28673835	15.27376274	1.982925809	2.80E-19	**ETV1**
	MA0473.3	169	36325	30.28673835	15.7199363	1.926645107	4.83E-18	**ELF1**
	MA1517.1	138	27711	24.7311828	11.99215842	2.062279526	8.25E-17	**KLF6**
	MA0062.3	158	36146	28.31541219	15.64247261	1.810162172	2.33E-14	**GABPA**

	motif	observed	background	percent.observed	percent.background	fold.enrichment	pvalue	motif.name
**Day 15 OSKLH > OSKL**	MA0599.1	1127	65239	51.04166667	28.23270266	1.807891624	9.26E-114	**KLF5**
	MA0039.4	1138	70417	51.53985507	30.47352386	1.69129948	3.27E-95	**KLF4**
	MA1515.1	780	43246	35.32608696	18.71505479	1.887575931	1.63E-76	**KLF2**
	MA1653.1	977	60460	44.24818841	26.16455192	1.691150245	9.12E-76	**ZNF148**
	MA0079.4	659	33768	29.84601449	14.61337395	2.042376701	2.74E-75	**SP1**
	MA0741.1	781	43918	35.37137681	19.0058682	1.861076613	7.70E-74	**KLF16**
	MA0746.2	677	36653	30.66123188	15.86188094	1.933013619	3.92E-68	**SP3**
	MA0685.1	560	27974	25.36231884	12.10597379	2.095025091	7.82E-66	**SP4**
	MA1564.1	620	32748	28.07971014	14.17196074	1.981356755	3.83E-65	**SP9**
	MA0516.2	638	34645	28.89492754	14.99290277	1.927240374	6.13E-63	**SP2**

Table S4. Karyotype analysis of primed and naive PSCs used in this experiment

	Cell line	Gender	Passage number	Karyotypic abnormalities (20 or 21 cells examined)	Representative abnormalities	Polyploid(%)
Primed	OSKLH_1	F	30	none		0%
	OSKLH_2	F	11	none		0%
	OSKLH_3	F	30	none		0%
	OSKLH_4	F	29	none		0%
	OSKLH_5	F	30	none		0%
	OSKLH_6	F	28	none		0%
	OSKLH_7	F	28	none		0%
	OSKLH_8	F	11	20/21	46,XX,t(6;7)(q23;q21)[20]	0%
	OSKLH_9	F	35	none		0%
	OSKLH_10	F	28	none		0%
Naive	Reset H9 ESC	F	85	19/20	46,XX,+21,der(21;21)(q10;q10)[5], 14 other abnormalities	32.3%
	nOSKL_1	F	16	18/20	48,XX,+11,+21[5], 13 other abnormalities	44.9%
	nOSKL_2	F	16	11/20		24.1%
	nOSKLH_1	F	16	14/20	46,XX,add(15)(p11.2)[2], 12 other abnormalities	48.5%
	nOSKLH_2	F	16	8/20		24.4%

Table S5. SNP genotyping array results of naive iPSCs generated in this study

No.	1		2	3	4	5	6	7	8		9
Type of CNV	gain	gain	loss	loss	gain	gain	loss	loss	gain	gain	gain
Location	chr1: 202,818,754-202,849,770	chr1: 203,144,833-203,158,972	chr1: 229,575,709-230,062,961	chr2: 11,451,984-11,796,774	chr4: 148,550,590-148,659,137	chr6: 162,803,457-162,847,889	chr7: 146,879,634-147,223,640	chr10: 24,647,899-24,736,083	chr15: 60,220,932-60,259,176	chr15: 60,585,694-60,638,256	chr18: 37,340,664-38,067,013
Size (bp)	31,017	14,140	487,253	344,791	108,548	44,433	344,007	88,185	38,245	52,563	726,350
t2iLGö+Y OSKL_1 (p12)	-	-	-	-	-	-	-	-	-	-	-
t2iLGö+Y OSKL_2 (p12)	-	-	-	-	-	-	-	-	-	-	-
t2iLGö+Y OSKL_3 (p12)	-	-	-	-	-	-	-	-	-	-	-
t2iLGö+Y OSKLH_1 (p12)	-	-	-	-	-	-	-	-	-	-	-
t2iLGö+Y OSKLH_2 (p12)	-	-	-	-	-	-	-	-	-	-	-
t2iLGö+Y OSKLH_3 (p12)	-	-	-	-	-	-	-	-	-	-	-
PXGLY OSKL_1 (p13)	-	-	-	-	-	-	-	-	-	-	-
PXGLY OSKL_2 (p13)	-	-	-	-	-	-	-	-	-	-	-
PXGLY OSKL_3 (p13)	-	-	-	-	-	-	-	-	-	-	-
PXGLY OSKLH_1 (p13)	-	-	-	-	-	-	-	-	-	-	-
PXGLY OSKLH_2 (p13)	-	-	-	-	-	-	-	-	-	-	-
PXGLY OSKLH_3 (p13)	-	-	-	-	-	-	-	-	-	-	-
t2iLGö+Y → PXGLY OSKL_1 (p13)	-	-	-	-	-	-	-	-	-	-	-
t2iLGö+Y → PXGLY OSKL_2 (p13)	-	-	-	-	-	-	-	-	-	-	-
t2iLGö+Y → PXGLY OSKL_3 (p13)	-	-	-	-	-	-	-	-	-	-	-
t2iLGö+Y → PXGLY OSKLH_1 (p13)	-	-	-	-	-	-	-	-	-	-	-
t2iLGö+Y → PXGLY OSKLH_2 (p13)	-	-	-	-	-	-	-	-	-	-	-
t2iLGö+Y → PXGLY OSKLH_3 (p13)	-	-	-	-	-	-	-	-	-	-	-
t2iLGö+Y → AXGY OSKL_1 (p13)	-	-	-	-	-	-	-	-	-	-	-
t2iLGö+Y → AXGY OSKL_2 (p17)	-	-	-	-	-	-	-	-	-	-	○
t2iLGö+Y → AXGY OSKL_3 (p17)	○	○	-	-	-	-	-	-	-	-	-
t2iLGö+Y → AXGY OSKLH_1 (p17)	-	-	-	-	-	-	-	-	-	-	-
t2iLGö+Y → AXGY OSKLH_2 (p17)	-	-	-	-	-	-	-	-	-	-	-
t2iLGö+Y → AXGY OSKLH_3 (p17)	-	-	-	-	-	-	-	-	-	-	-
5iLA → AXGY OSKL_1 (p17)	-	-	-	-	-	-	-	-	-	-	-
5iLA → AXGY OSKL_2 (p17)	-	-	-	-	-	-	-	-	-	-	-
5iLA → AXGY OSKL_3 (p17)	-	-	-	-	-	○	-	-	○	○	-
5iLA → AXGY OSKLH_1 (p17)	-	-	-	-	-	-	-	-	-	-	-
5iLA → AXGY OSKLH_2 (p17)	-	-	○	○	○	-	-	○	-	-	-
5iLA → AXGY OSKLH_3 (p17)	-	-	-	-	-	-	○	-	-	-	-

SUPPLEMENTARY EXPERIMENTAL PROCEDURES

Construction of SeV-*H1FOO*, SeV-*H1FOO-DD*, and SeV-*DD-H1FOO* vectors

The insert sequence containing the open reading frame of *H1FOO*, *H1FOO-DD*, and *DD-H1FOO* genes were amplified by PCR from cDNAs and inserted in the NotI site of the plasmids containing the F-defecive SeV vector backbone. The gene specific primers were designed to introduce NotI sites on both ends of the amplified fragment and SeV-specific transcriptional regulatory signal sequences were also added after the coding sequence. We obtained DD sequence information from a previous report (Banaszynski et al., 2006). In *H1FOO-DD, DD* was inserted downstream of the C-terminus of *H1FOO* and in *DD-H1FOO*, upstream of the N-terminus of *H1FOO.* The TS15 mutations are reported elsewhere (Schlaeger, 2018).The plasmids pSeV18+H1FOO/TS15ΔF, pSeV18+H1FOO-DD/TS15ΔF, and pSeV18+DD-H1FOO/TS15ΔF were constructed according to the previously reported methods (Inoue et al., 2003) . SeV-H1FOO, SeV-H1FOO-DD, and SeV-DD-H1FOO vectors were recovered from these plasmids and propagated as previously described (Komuta et al., 2016). When stabilizing H1FOO-DD to prevent degradation, 1 μM Shield1 (Takara) was added to the cell culture medium.

iPSC colony formation assay

For both naive and primed iPSCs, iPSC colony counts were performed on day 14 after the SeV vector infection. We visually counted AP-positive colonies using an optical microscope (Olympus) after cells were fixed in 4% paraformaldehyde (Nacalai Tesque) for 15 minutes at room temperature and then stained using the Alkaline Phosphatase Assay Kit (Sigma) according to the manufacturer's protocol.

Immunocytochemistry

The cells plated on glass bottomed dishes (AGC) were washed once with phosphate-buffered saline (PBS) and fixed with 4% paraformaldehyde (Nacalai Tesque) for 15min at room temperature. The cells were permeabilized with 0.5% Triton X-100 (Nacalai Tesque) in PBS for 10 min at room temperature. After blocking with ImmunoBlock (KAC) for 15 min, the cells were incubated at room temperature for 60 min with the primary antibodies which were diluted in ImmunoBlock. After being washed twice with PBS, the samples were exposed to fluorescence-conjugated secondary antibodies along with 4',6-diamidino-2-phenylindole (DAPI, Invitrogen) for 60 min at room temperature. Images were obtained using a BZ-X710 imaging system (KEYENCE). The list of antibodies used in this study is provided in the key resources table.

Trilineage differentiation

We used STEMdiff Trilineage Differentiation Kit (STEMCELL) to differentiate iPSCs into trilineage. 4x10^5 cells for ectoderm, 2x10^5 cells for mesoderm, and 4x10^5 cells for endoderm were seeded into 6 wells coated with Matrigel (Corning). For ectoderm differentiation, we started

culturing in ectoderm medium instead of mTeSR1 (STEMCELL) from the first day. To promote differentiation, we added 10 uM SB431542 (Wako) to the ectoderm medium. In mesoderm and endoderm differentiation, we cultured iPSCs in mTeSR1 on the first day. We collected and analyzed ectoderm differentiated cells on day 7 and mesoderm and endoderm differentiated cells on day 5.

Cardiomyocyte differentiation

Four days prior to the start of differentiation, 4x10^4 iPSCs were seeded, and maintained by changing iPSC media every other day. On Day 0, we replaced the medium with RPMI (Wako) containing 2 % B-27 supplement minus insulin (B-27MI, Gibco), 6 uM CHIR (Wako) and 1 ng/ml BMP4 (R&D). 24 hours later on Day 1, we washed cells with PBS and replaced with RPMI containing only B-27MI. On Day 3, we replaced the medium with RPMI containing B-27MI and 5 uM IWR-1 (Sigma). On Day 7, the medium was replaced with MEM alpha (Gibco) containing 5 % FBS, and cells were collected and analyzed on Day 10.

Naive PSC-derived trophectoderm (TE) differentiation

For TE differentiation, naive PSCs were dissociated, and iMEF feeder cells were removed as described above. 500,000 cells were plated in laminin 511-E8 (0.15 μg/cm^2 iMatrix 511; Nippi)-coated 6 wells with initial TE differentiation medium: NDiff227, 2 μM A83-01 (Wako), 2 μM PD0325921 (Sigma) and 10 ng/mL BMP4 (R&D). The next day, the medium was changed to NDiff227, 2 μM A83-01 (Wako), 2 μM PD0325921 (Sigma) and 1 μg/mL JAK inhibitor I (Sigma). At day 2, the medium was changed again. At day 3, we harvested the cells using Accutase (Innovative Cell Technologies) for 30 min and analyzed TACSTD2 and HAVCR1 expression by flow cytometry.

Flow cytometry

Dissociated cells were stained on ice for 20 min with fluorescent conjugated antibodies. Analyses were performed using the BD LSR Fortessa (BD Biosciences) flow cytometer equipped with FACS Diva software (BD biosciences) and Gallios flow cytometer (Beckman Coulter). The data were analyzed using FlowJo software (LLC).

RNA and DNA extraction and real-time quantitative PCR (qPCR)

When extracting nucleic acids from naive PSCs, iMEFs were removed by incubating naive PSCs for 2 hours at 37°C on a gelatin-coated dish before sampling for nucleic acid extraction. Total RNA and DNA were extracted from cell lysates using the AllPrep DNA/RNA Mini Kit (QIAGEN), and the RNA was incubated with RNase-Free DNase Set (QIAGEN) to remove genomic DNA. For qPCR, the reverse transcription reaction was performed with 1 μg of DNase-treated RNA using PrimeScript RT Master Mix (Takara) containing oligo dT primer and random 6 mers. qPCR analysis was performed on StepOne Plus (Applied Biosystems) or QuantStudio3 (Applied Biosystems) using

TaqMan Fast Advanced Master Mix (Applied Biosystems) or Fast SYBR Green Master Mix (Applied Biosystems), and Scorecard analysis was performed on QuantStudio 12K (Applied Biosystems) or StepOne Plus (Applied Biosystems) using TaqMan Gene Expression Master Mix (Applied Biosystems) according to the manufacturer's protocol.

Western blot analysis

The cells were lysed using M-PER Mammalian Protein Extraction Reagent (Thermo) containing protease inhibitor (Sigma) and phosphatase inhibitor cocktail 2 and 3 (Sigma). Protein concentration was determined using Pierce BCA Protein Assay Kit (Thermo).

For detection, we used WES or JESS system (ProteinSimple) which performs protein separation and detection using an automated capillary electrophoresis system. Signals were detected with an HRP-conjugated secondary anti-rabbit antibody and were visualized using Compass software (ProteinSimple).

Cell metabolism analysis

Oxygen consumption rate and extracellular acidification rate were measured using a Seahorse XF96 Analyzer (Agilent) and Seahorse XF Cell Mito Stress Test Kit (Agilent). PSCs were dissociated and cells were incubated on gelatin for 2 hours at 37 °C to remove feeder cells. Seahorse plate was coated with Matrigel (Corning) for 1hr at 37 °C prior cell seeding. Cells were seeded at 100,000 cells per well and incubated overnight. The next day, culture media were exchanged for XF Base Medium (Agilent) supplemented with 2 mM pyruvate, 20 mM glucose and 2 mM Glutamax (Gibco) with an adjusted pH of 7.4 and cells incubated at 37 °C in atmospheric CO_2 incubator for 1 hr. During the mito stress kit experiment, Oligomycin (6 μM), FCCP (2 μM), Rotenone and Antimycin-A (1 μM) were injected at indicated time points. Metabolic profiling was performed by mapping the OCR and ECAR at eighth time point from the beginning of the analysis as indicated in Figure S5D. The spare respiratory capacity was calculated as the difference between basal and FCCP-induced OCR (Nicholas et al., 2017).

RNA-FISH

Dissociated naive iPSCs were incubated on gelatin for 2 hours at 37 °C to remove iMEF feeder cells. Then the cells were seeded on Matrigel-coated slides in PSC medium. The next day, the cells were fixed in 4 % paraformaldehyde for 15 min at room temperature. The slides were treated with 0.2 M HCl for 20 min, permeabilized with 0.2% Triton X-100 for 10 min, digested with pepsin solution (0.005% in 0.1 M HCl) at 37 ºC for 2-6 min, and dehydrated. Bacterial artificial chromosomes (BACs) RP11-155O24 and RP11-256P2 were used to generate *HUWE1* and *UTX* RNA FISH probes, respectively. BAC DNAs were labelled by nick-translation with Cy5-dUTP (RP11-155O24) and Cy3-dUTP (RP11-256P2). The labelled probes and the *XIST* RNA FISH probe (Chromosome Science Labo) were mixed with sonicated salmon sperm DNA and Cot-1 DNA in hybridization

solution. The probes were denatured at 85ºC for 10 min, applied to the pretreated slides, covered with cover slips, and hybridized at 37ºC overnight. The slides were then washed with 50% formamide / 2xSSC at 37ºC for 20 min, 1xSSC for 15 min at room temperature, counterstained using DAPI, and mounted. The FISH images were captured with the CW4000 FISH application program (Leica Microsystems Imaging Solution) using a cooled CCD camera mounted on a Leica DMRA2 microscope. We examined the expression of *UTX*, *HUWE1*, and *XIST* in 100 cells per clone.

Chromosome analysis

The samples in exponentially growing phase were incubated with final concentration of 0.02 μg/mL Metaphase Arresting Solution (Genial Genetic Solutions) for 90 min at 37 ℃. The cells were collected in tubes and subjected to hypotonic treatment with 0.075 M KCl solution for 30 min at 37 ℃. After fixing the cells with Carnoir's fixative (methanol: acetic acid ratio 3 : 1), the cell suspension was dropped a few drops on the glass slides and air dried.

For conventional Giemsa staining (non-banding technique), after staining the slides with Giemsa solution/phosphate buffer, the slides were observed by BX-51 or BX-53 microscopy (OLYMPUS). A total of 30 metaphases were counted for the chromosomal counts and these metaphase chromosomes were classified into distinguishable groups (A~G) based on morphological features.

For GTG method (G-banding), after trypsin treatment and Giemsa staining, the slides were observed by AxioImagerZ2 microscopy (CarlZeiss microscopy) equipped with CoolCube1m CCD camera (MetaSystems) and Metafer Slide Scanning System (MetaSytems). At least 20 metaphases were analyzed based on G-band by using Ikaros Karyotyping System (MetaSystems).

Any samples suspected to be chromosomal abnormality and required detail analysis were further analyzed by mBAND (multicolor chromosome banding) method using an appropriate mBAND Probe kit (MetaSystems). For mBAND analysis, we used Isis FISH Imaging System.

SNP genotyping array

Copy number variation (CNV) was evaluated with SNP genotyping array. Genomic DNA was hybridized onto the Infinium OmniExpress24 v1.4 DNA Analysis Kit (Illumina), and intensities were scanned by iScan (Illumina) following the manufacturer’s protocol. After exporting a final report using GenomeStudio (2.0.4) (Illumina), CNV analysis was performed with PennCNV (1.0.3) (Wang et al., 2007), Mosaic Alteration Detection-MAD (1.0.1) (González et al., 2011) and GWAS tools (1.16.1) (Gogarten et al., 2012). Only CNVs in test samples against control samples were reported. Log R Ratios and B-Allele Frequencies were visualized with GenomeStudio.

RNA sequencing and data analysis

RNA sequencing libraries were made from 100 ng of total RNA as starting materials with the TruSeq Stranded mRNA LT Sample Prep Kit Set A (Illumina) or TruSeq Stranded Total RNA Library Prep Gold (Illumina) following the manufacturer’s protocol. For Hiseq2500, clusters were generated with

the HiSeq PE Cluster Kit v4-cBot (Illumina) using illumina cBot. Sequencing was performed with the HiSeq SBS Kit v4 using HiSeq2500 (2 x 126 PE mode). NovaSeq 6000 (2 x 101 PE mode) with the NovaSeq 6000 S1 Reagent Kit v1.5 (Illumina) and NextSeq 500 (76 SE mode) with the NextSeq 500/550 High Output Kit v2.5 (Illumina) was also used for sequencing. FASTQ files were generated from bcl files using bcl2fastq v2.17.1.14 (Illumina) and processed using ENCODE long-rna-seq-pipeline v2.3.4. Briefly, the sequenced reads were mapped to the human reference genome (GRCh38) using TopHat 2.1.1 (Kim et al., 2013) or STAR 2.5.1b (Dobin et al., 2013) with GENCODE v24 gene annotations, the normalized gene expression data was calculated using RSEM 1.2.23 (Li and Dewey, 2011), and the gene count data was obtained using featureCounts bundled in Subread 1.5.1 (Liao et al., 2014). For the characterization of our PSC lines, the data sets of GSE59435 (Theunissen et al., 2014) and GSE75868 (Theunissen et al., 2016) obtained from GEO and supplemental data in Yan *et al*. (Yan et al., 2013) and Takashima *et al*. (Takashima et al., 2014) were used. The expression values of 4,720 genes included in all data were normalized by quantile among samples, and z-scores for each gene were used for the PCA. Log2-scaled, quantile normalized FPKM values were used for the expression heatmap of PSC markers.

DNA methylation analysis

The bisulfite conversion of 500 ng genomic DNA was performed using the EZ DNA Methylation Kit (Zymo Research), and the global DNA methylation status was profiled using Infinium Human Methylation 450K or EPIC BeadChip Kit (illumina) according to the manufacturer's protocols. After exporting the DNA methylation values using GenomeStudio V2011.1, data processing was conducted using the "minfi" package in R 3.6.3 (Aryee et al., 2014). In total, 424,444 probes common between 450K and EPIC and not located at known SNP sites were used for the PCA of PSC samples.

Single cell RNA-seq analysis

Single cell RNA-seq libraries were prepared according to the manufacturer's protocol using the 10x Genomics Chromium Next GEM Single Cell 3' Kit v3 and Single Index Kit, where the number of target cells was 2500. The libraries were sequenced with Hiseq2500, where clusters were generated with the HiSeq PE Cluster Kit v4-cBot (Illumina) using illumina cBot and sequencing was performed with the HiSeq SBS Kit v4 (28-8-0-91 cycles). Raw sequencing data was converted to the standard FASTQ files by executing cellranger mkfastq (3.1.1, 10x Genomics). Gene counts of each sample were generated by executing cellranger count (3.1.1, 10x Genomics) with refdata-cellranger-GRCh38-3.0.0 as the reference dataset. Single cell RNA-seq data processing including quality control, count normalization, and clustering was performed using Seurat (Butler et al., 2018) version 3.2.2. Cells with gene numbers less than 4000 or larger than 11000 were removed. Cells with higher proportion of mitochondrial mRNA (proportion of mitochondrial mRNA > 25) were also removed. After the quality control, each count matrix was log-normalized by pool, and then merged. Principal

component analysis (PCA) of the combined object was performed by RunPCA function using 2000 variable genes selected by FindVariableFeatures function with vst method. For the visualization, uniform manifold approximation and projection (UMAP) dimensional reduction was used with 30 dimensions of PCA to use as input features. To detect the clusters, k-nearest neighbor (kNN) graph was constructed using Seurat function, FindNeighbors and FindClusters with default parameters other than resolution = 0.5. Data visualization was performed using DimPlot, FeaturePlot, DotPlot and VlnPlot functions in Seurat.

Single cell ATAC-seq analysis

Single cell ATAC-seq libraries were prepared according to the manufacturer's protocol using the 10x Genomics Chromium Single Cell ATAC Reagent Kit v1, where the number of target cells was 2500. The libraries were sequenced with Hiseq2500, where clusters were generated with the HiSeq PE Cluster Kit v4-cBot (Illumina) using illumina cBot and sequencing was performed with the HiSeq SBS Kit v4 (50-8-16-50 cycles). Raw sequencing data was converted to the standard FASTQ files by executing cellranger-atac mkfastq (1.1.0, 10x Genomics). Fastq files from single cell ATAC-seq were mapped by CellRanger using the hg38 reference (refdata-cellranger-atac-GRCh38-1.2.0). Mapping was performed using cellranger-atac count function by pool and then the data aggregated by cellranger-atac aggr function. The peak size of aggregated 231,076 peak regions in peaks.bed output were re-sized into peak-centered 500 bp non-redundant regions and then all pools were re-mapped by cellranger-atac reanalyze function using the common 500bp regions.

Count data and metadata from CellRanger output was processed following the Signac "Guided analyses" (https://satijalab.org/signac/articles/overview.html) using Seurat (v3.2.2), Signac (Stuart et al., 2021) (v1.1.0), TFBSTools (Tan and Lenhard, 2016) (v1.22.0) and chromVAR (Schep et al., 2017) (c1.6.0). For quality control, the mapping rate of reads in transcript start site (TSS) regions (TSS.enrichment) was checked and all data were retained because all cells had TSS.enrichment more than 2%. The median of ATAC counts per cell were 28,455 ± 7,526.4 (standard deviation). The percent reads in peak regions (pct_reads_in_peaks) were calculated as % of (peak region fragments) /(passed filters).

All sample pools were merged and normalized by term frequency inverse document frequency (TFI-DF) normalization using RunTFIDF function with default parameters (Stuart et al., 2019). For the visualization, the singular value decomposition and UMAP dimensional reduction was performed using latent semantic indexing (LSI) components 2 to 50 (Cusanovich et al., 2018). To detect the clusters, kNN graph was constructed using Seurat function, FindNeighbors using LSI components 2 to 30 and FindClusters with smart local moving algorithm (algorithm = 3) (Butler et al., 2018). Data visualization was performed using DimPlot, FeaturePlot, DotPlot and VlnPlot functions in Seurat.

Cluster prediction using scRNA-seq data

To integrate the scATAC-seq data with scRNA-seq clusters, we computed gene activity counts for

each gene in each cell using GeneActivity function in Signac with default parameters. This gene activity counts were anchored with the cell type information of scRNA-seq data using FindTransferAnchors with a canonical correlation analysis reduction method and TransferData function using the LSI components 2 to 30 for weighted reduction.

Motif enrichment analysis by chromVAR

Motif enrichment analysis of scATAC-seq data was performed using AddMotifs function of TFBSTools package using a matrix set from JASPAR2020 datasets (Fornes et al., 2020). Differential accessibility to the motifs between groups were calculated by constructing a logistic regression model using FindMarkers function. Motif activities of individual cells were calculated using RunChromVAR function in Signac. Motif activities were vidualized using FeaturePlot and VlnPlot in Seurat.

SUPPLEMENTARY REFERENCES

Aryee, M.J., Jaffe, A.E., Corrada-Bravo, H., Ladd-Acosta, C., Feinberg, A.P., Hansen, K.D., and Irizarry, R.A. (2014). Minfi: a flexible and comprehensive Bioconductor package for the analysis of Infinium DNA methylation microarrays. Bioinformatics *30*, 1363-1369.

Banaszynski, L.A., Chen, L.C., Maynard-Smith, L.A., Ooi, A.G., and Wandless, T.J. (2006). A rapid, reversible, and tunable method to regulate protein function in living cells using synthetic small molecules. Cell *126*, 995-1004.

Butler, A., Hoffman, P., Smibert, P., Papalexi, E., and Satija, R. (2018). Integrating single-cell transcriptomic data across different conditions, technologies, and species. Nature biotechnology *36*, 411-420.

Cusanovich, D.A., Hill, A.J., Aghamirzaie, D., Daza, R.M., Pliner, H.A., Berletch, J.B., Filippova, G.N., Huang, X., Christiansen, L., DeWitt, W.S., *et al.* (2018). A Single-Cell Atlas of In Vivo Mammalian Chromatin Accessibility. Cell *174*, 1309-1324.e1318.

Dobin, A., Davis, C.A., Schlesinger, F., Drenkow, J., Zaleski, C., Jha, S., Batut, P., Chaisson, M., and Gingeras, T.R. (2013). STAR: ultrafast universal RNA-seq aligner. Bioinformatics *29*, 15-21.

Fornes, O., Castro-Mondragon, J.A., Khan, A., van der Lee, R., Zhang, X., Richmond, P.A., Modi, B.P., Correard, S., Gheorghe, M., Baranašić, D., *et al.* (2020). JASPAR 2020: update of the open-access database of transcription factor binding profiles. Nucleic Acids Res *48*, D87-d92.

Inoue, M., Tokusumi, Y., Ban, H., Kanaya, T., Tokusumi, T., Nagai, Y., Iida, A., and Hasegawa, M. (2003). Nontransmissible virus-like particle formation by F-deficient sendai virus is temperature sensitive and reduced by mutations in M and HN proteins. J Virol *77*, 3238-3246.

Kim, D., Pertea, G., Trapnell, C., Pimentel, H., Kelley, R., and Salzberg, S.L. (2013). TopHat2: accurate alignment of transcriptomes in the presence of insertions, deletions and gene fusions. Genome biology *14*, R36.

Komuta, Y., Ishii, T., Kaneda, M., Ueda, Y., Miyamoto, K., Toyoda, M., Umezawa, A., and Seko, Y. (2016).

In vitro transdifferentiation of human peripheral blood mononuclear cells to photoreceptor-like cells. Biol Open *5*, 709-719.

Li, B., and Dewey, C.N. (2011). RSEM: accurate transcript quantification from RNA-Seq data with or without a reference genome. BMC Bioinformatics *12*, 323.

Liao, Y., Smyth, G.K., and Shi, W. (2014). featureCounts: an efficient general purpose program for assigning sequence reads to genomic features. Bioinformatics *30*, 923-930.

Nicholas, D., Proctor, E.A., Raval, F.M., Ip, B.C., Habib, C., Ritou, E., Grammatopoulos, T.N., Steenkamp, D., Dooms, H., Apovian, C.M.*, et al.* (2017). Advances in the quantification of mitochondrial function in primary human immune cells through extracellular flux analysis. PloS one *12*, e0170975.

Schep, A.N., Wu, B., Buenrostro, J.D., and Greenleaf, W.J. (2017). chromVAR: inferring transcription-factor-associated accessibility from single-cell epigenomic data. Nature methods *14*, 975-978.

Schlaeger, T.M. (2018). Nonintegrating Human Somatic Cell Reprogramming Methods. Adv Biochem Eng Biotechnol *163*, 1-21.

Stuart, T., Butler, A., Hoffman, P., Hafemeister, C., Papalexi, E., Mauck, W.M., 3rd, Hao, Y., Stoeckius, M., Smibert, P., and Satija, R. (2019). Comprehensive Integration of Single-Cell Data. Cell *177*, 1888-1902.e1821.

Stuart, T., Srivastava, A., Madad, S., Lareau, C.A., and Satija, R. (2021). Single-cell chromatin state analysis with Signac. Nature methods *18*, 1333-1341.

Takashima, Y., Guo, G., Loos, R., Nichols, J., Ficz, G., Krueger, F., Oxley, D., Santos, F., Clarke, J., Mansfield, W.*, et al.* (2014). Resetting transcription factor control circuitry toward ground-state pluripotency in human. Cell *158*, 1254-1269.

Tan, G., and Lenhard, B. (2016). TFBSTools: an R/bioconductor package for transcription factor binding site analysis. Bioinformatics *32*, 1555-1556.

Theunissen, T.W., Friedli, M., He, Y., Planet, E., O'Neil, R.C., Markoulaki, S., Pontis, J., Wang, H., Iouranova, A., Imbeault, M.*, et al.* (2016). Molecular Criteria for Defining the Naive Human Pluripotent State. Cell stem cell.

Theunissen, T.W., Powell, B.E., Wang, H., Mitalipova, M., Faddah, D.A., Reddy, J., Fan, Z.P., Maetzel, D., Ganz, K., Shi, L.*, et al.* (2014). Systematic identification of culture conditions for induction and maintenance of naive human pluripotency. Cell stem cell *15*, 471-487.

Yan, L., Yang, M., Guo, H., Yang, L., Wu, J., Li, R., Liu, P., Lian, Y., Zheng, X., Yan, J.*, et al.* (2013). Single-cell RNA-Seq profiling of human preimplantation embryos and embryonic stem cells. Nature structural & molecular biology *20*, 1131-1139
