# Supplementary material for: H1FOO-DD promotes efficiency and uniformity in reprogramming to naive pluripotency
Source: Stem Cell Reports. 2024 May 2;19(5):710–28. doi: 10.1016/j.stemcr.2024.04.005 (PMC11103934; doi:10.1016/j.stemcr.2024.04.005)
Supplement: Document S2. Article plus supplemental information [file mmc2.pdf]

Stem Cell Reports
Article
OPEN ACCESS

H1FOO-DD promotes efficiency and uniformity in reprogramming to naive pluripotency

Akira Kunitomi,[1,2,14,*] Ryoko Hirohata,[1,3] Mitsujiro Osawa,[1,9] Kaho Washizu,[2] Vanessa Arreola,[2,10] Norikazu Saiki,[1,11] Tomoaki M. Kato,[1,3] Masaki Nomura,[1,3] Haruko Kunitomi,[2] Tokiko Ohkame,[1,12] Yusuke Ohkame,[1,13] Jitsutaro Kawaguchi,[4] Hiroto Hara,[4] Kohji Kusano,[4] Takuya Yamamoto,[1,5,6] Yasuhiro Takashima,[1] Shugo Tohyama,[7] Shinsuke Yuasa,[7] Keiichi Fukuda,[7] Naoko Takasu,[1,3] and Shinya Yamanaka[1,2,3,8]

[1]Center for iPS Cell Research and Application (CiRA), Kyoto University, Kyoto 606-8507, Japan
[2]Gladstone Institute of Cardiovascular Disease, San Francisco, CA 94158, USA
[3]CiRA Foundation, Kyoto 606-8397, Japan
[4]ID Pharma Co., Ltd, Ibaraki 300-2611, Japan
[5]Institute for the Advanced Study of Human Biology (WPI-ASHBi), Kyoto University, Kyoto 606-8501, Japan
[6]Medical-risk Avoidance Based on iPS Cells Team, RIKEN Center for Advanced Intelligence Project (AIP), Kyoto 606-8507, Japan
[7]Department of Cardiology, Keio University School of Medicine, Tokyo 160-8582, Japan
[8]Department of Anatomy, University of California, San Francisco, San Francisco, CA 94143, USA
[9]Present address: Thyas Co, Ltd., Kyoto 606–8501, Japan
[10]Present address: Center for Definitive and Curative Medicine, Stanford University, Stanford CA 94305, USA
[11]Present address: Institute of Research, Tokyo Medical and Dental University (TMDU), Tokyo 113–8510, Japan
[12]Present address: Yukioka School of Allied and Health Professions Department of Clinical Laboratory, Osaka 530-0061, Japan
[13]Present address: Chromosome Engineering Research Center, Tottori University, Yonago 683–8503, Japan
[14]Lead contact
*Correspondence: akira.kunitomi@gladstone.ucsf.edu
https://doi.org/10.1016/j.stemcr.2024.04.005

SUMMARY

Heterogeneity among both primed and naive pluripotent stem cell lines remains a major unresolved problem. Here we show that expressing the maternal-specific linker histone *H1FOO* fused to a destabilizing domain (*H1FOO-DD*), together with *OCT4*, *SOX2*, *KLF4*, and *LMYC*, in human somatic cells improves the quality of reprogramming to both primed and naive pluripotency. *H1FOO-DD* expression was associated with altered chromatin accessibility around pluripotency genes and with suppression of the innate immune response. Notably, *H1FOO-DD* generates naive induced pluripotent stem cells with lower variation in transcriptome and methylome among clones and a more uniform and superior differentiation potency. Furthermore, we elucidated that upregulation of *FKBP1A*, driven by these five factors, plays a key role in *H1FOO-DD*-mediated reprogramming.

INTRODUCTION

Human induced pluripotent stem cells (iPSCs) generated from somatic cells using conventional reprogramming methods exhibit "primed pluripotency," in that like the post-implantation epiblast; their differentiation into extraembryonic tissues is limited. On the other hand, human iPSCs with "naive pluripotency" can differentiate into both embryonic and extra-embryonic lineages, demonstrating characteristics similar to preimplantation epiblast cells and mouse iPSCs (Nichols and Smith, 2009; Weinberger et al., 2016). This capacity of naive pluripotent cells is critical for research into developmental biology and regenerative medicine, such as efforts to create blastoids and placentas (Io et al., 2021; Liu et al., 2021; Yu et al., 2021). Recently, methods for generating naive human iPSCs by introducing reprogramming factors into somatic cells using Sendai virus vectors have been reported (Kilens et al., 2018; Kunitomi et al., 2022; Liu et al., 2017) that will greatly advance these efforts.

However, both primed and naive iPSCs still face challenges for use in basic research and clinical applications (Yamanaka, 2020). In addition to the low generation efficiency, heterogeneity among cell lines, such as in gene expression, DNA methylation, and differentiation potency, is a critical issue because it can cause problems with the reproducibility of iPSC-based research and the safety of regenerative medicine using iPSC-derived differentiated cells. Notably, heterogeneity occurs even in reprogramming from the same somatic cells with the same genetic background (Francesconi et al., 2019), suggesting that conventional reprogramming methods are stochastically incomplete in some cells. Moreover, while there are many reports of efforts to improve the efficiency of primed iPSC colony generation (Liu et al., 2020), there are almost no reports of improved naive iPSC generation efficiency and very few reports of attempts to reduce human naive iPSC heterogeneity.

In this study, we focus on the maternally specific linker histone H1FOO, which is abundant in human oocytes and plays an important role in chromatin structural

© 2024 The Author(s).
This is an open access article under the CC BY license (http://creativecommons.org/licenses/by/4.0/).

changes in the embryonic nucleus immediately after fertilization. We show that *H1FOO-DD*, which is a combination of *H1FOO* and a destabilizing domain (*DD*), enhances reprogramming to primed and naive human iPSCs. Adding *H1FOO-DD* to the set of reprogramming factors *OCT4*, *SOX2*, *KLF4*, and *LMYC* (OSKL) promotes the function of the *POU* family and *KLF/SP* family, which play important roles in the early stages of reprogramming. *H1FOO-DD* also drives *FKBP1A* upon co-expression with other reprogramming factors, resulting in suppression of the innate immune response, inflammatory response, and apoptosis during reprogramming. As a result, *H1FOO-DD* enhances the efficiency of reprogramming to a primed state and improves the differentiation potency of the generated iPSCs in some lineages. Moreover, *H1FOO-DD* also improves the reprogramming efficiency to a naive state and produces naive iPSCs with more uniform characteristics and superior differentiation potency.

RESULTS

H1FOO-DD enhances reprogramming to primed pluripotency

We first examined the expression of *H1FOO* during the reprogramming of human dermal fibroblasts (HDFs). We used Sendai virus (SeV) vector for reprogramming the HDFs, which is one of the widely used methods for generating human iPSCs because of its high gene expression efficiency (Fusaki et al., 2009; Li et al., 2000). Moreover, we used *LMYC* instead of the proto-oncogene *CMYC*, which is reported to be more efficient in human iPSC reprogramming with OSKL (Akifuji et al., 2021; Nakagawa et al., 2008, 2010). No expression of *H1FOO* was observed in HDFs before or during the reprogramming process, up to day 5 after infection with OSKL, and *H1FOO* was also undetectable in H9 human embryonic stem cells (H9 ESCs) (Figure 1A). Thus, *H1FOO* is not endogenously expressed during the reprogramming of human iPSCs or in its end products, pluripotent stem cells (PSCs).

H1FOO is abundantly expressed in human oocytes, but its expression is known to be rapidly lost after fertilization (Ooga et al., 2016; Tanaka et al., 2001). Based on these findings, we hypothesized that we could recapitulate the developmental H1FOO expression pattern by constructing SeV vectors containing *H1FOO* with a system that rapidly degrades H1FOO. Therefore, we constructed not only a SeV-*H1FOO* vector which continuously and strongly expresses *H1FOO*, but also SeV-*H1FOO-DD* vectors with a destabilizing domain (DD) (Banaszynski et al., 2006) fused to the 3′ or 5′ side of *H1FOO* (SeV-*H1FOO-DD* and SeV-*DD-H1FOO*, respectively, Figure 1B). DD is a domain of the FKBP12 protein with a F36V mutation, and adding it to another protein results in rapid proteasomal degradation of the entire fusion product. Moreover, the addition of cell-permeable DD ligand named Shield1 protects DD-fusion proteins from degradation (Banaszynski et al., 2006). When HDFs were infected with SeV-*H1FOO-DD*, H1FOO localized to the nucleus (Figure 1C), and treatment with Shield1 significantly increased protein levels of H1FOO-DD (Figure 1D). This confirmed that DD promotes H1FOO degradation and that Shield1 works to prevent degradation of DD-fused protein as previously reported. Next, we sampled cells from day 1 to day 5 after SeV-H1FOO or SeV-H1FOO-DD infection and measured H1FOO protein expression levels. From day 2, H1FOO and H1FOO-DD were highly expressed, but H1FOO-DD degraded more rapidly than H1FOO, with significantly lower levels from day 4 onward (Figure 1E). This suggests that the DD-fused H1FOO protein is degraded and active in the cells for a shorter amount of time than un-tagged H1FOO.

Next, we examined if the addition of *H1FOO* or *DD*-fused *H1FOO* to OSKL improves the reprogramming efficiency of HDFs. To investigate whether the different vector backbones alter reprogramming efficiency, we also tested exogenous expression of OSKL plus *H1FOO* in the PiggyBac system. In addition, we overexpressed somatic H1 subtypes other than *H1FOO*, such as *HIST1H1A* and *H1F0* with OSKL using the PiggyBac system to check their effect. After introducing these vectors into HDFs, we compared the efficiency of iPSC generation on day 14 (Figure 1F). Regardless of vector type, neither *H1FOO* nor *DD* improved reprogramming efficiency, and somatic H1 markedly decreased the efficiency. In contrast, *H1FOO-DD* and *DD-H1FOO* significantly enhanced iPSC colony generation.

In the conventional method of iPSC generation using SeV vectors, iPSCs immediately after generation still express many SeV vector-derived pluripotency genes. Thus, some of the cells in the culture may have undergone incomplete reprogramming and be mixed in the generated iPSC population. To solve this problem and achieve a more accurate comparison of reprogramming efficiency, we used the CytoTuneEX-iPS kit for iPSC generation. With this approach, the SeV vectors are degraded and removed early after the generation of iPSCs because the target sequence of miR-367, which is specifically highly expressed in human PSCs (Zhang et al., 2015), is placed tandemly downstream of the P gene of the SeV vectors. Applying this approach to two types of HDFs and peripheral blood mononuclear cells (PBMCs) showed that *H1FOO-DD* markedly improved reprogramming efficiency in every cell type, with a maximum difference of about 8-fold compared to OSKL only (Figure 1G). This result suggests that *H1FOO-DD* generated more iPSCs regardless of the degree of transgene persistence. In summary, *H1FOO-DD* and *DD-H1FOO* have a positive effect on the generation of human primed iPSCs.

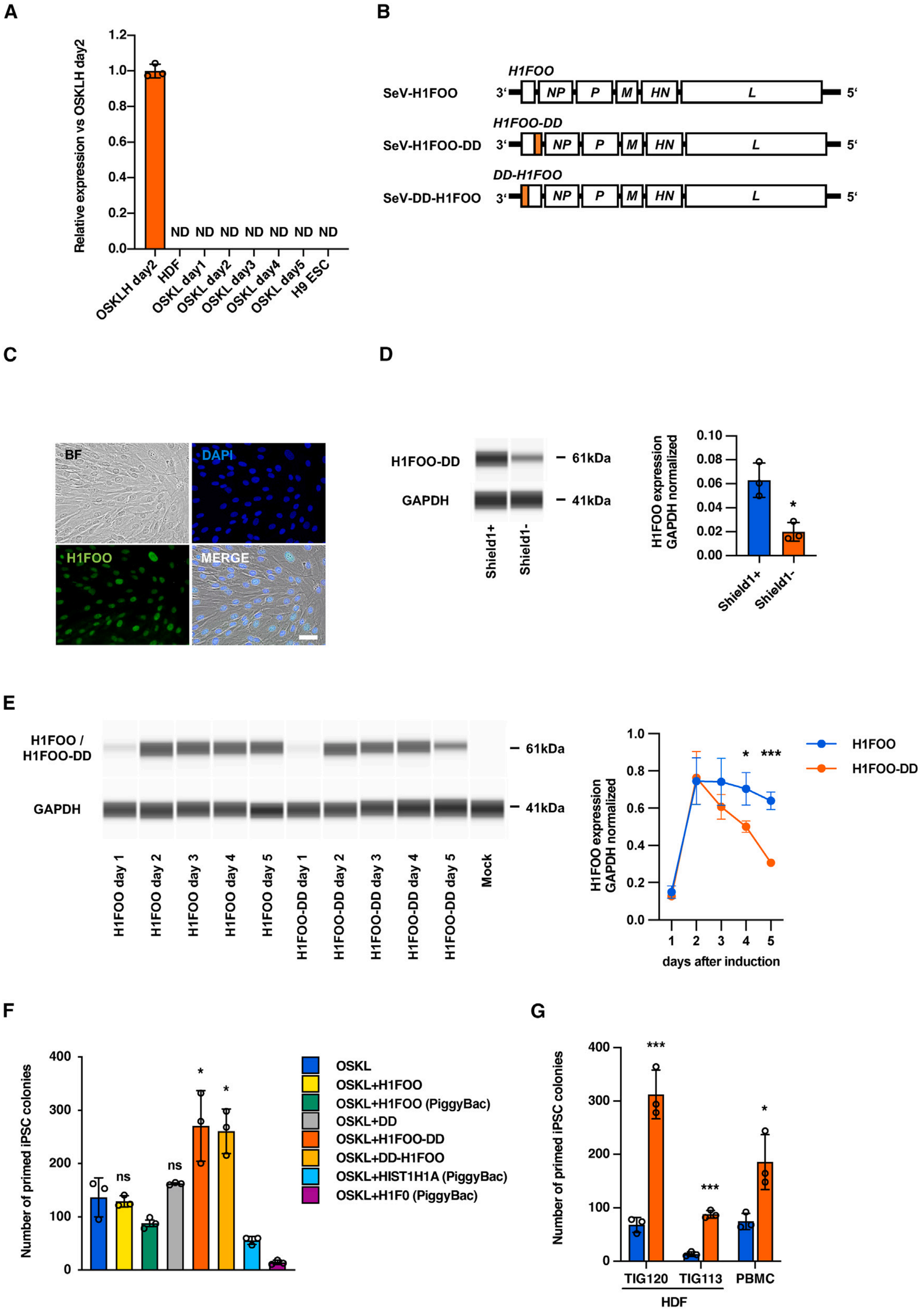

(legend on next page)

We then infected HDFs with OSKL or OSKL plus SeV-*H1FOO-DD* (OSKLH) and generated eight clones of iPSCs from each reprogramming cocktail to compare their characteristics. The OSKL group was infected with SeV-Mock instead of *H1FOO-DD* to achieve equal total multiplicity of infection (MOI). We first compared the transcriptome using bulk RNA sequencing (RNA-seq) and DNA methylation using DNA methylation array in these two groups as well as H9 ESCs and 201B7 iPSCs as PSC controls. Differentially expressed genes (DEGs) or differentially methylated regions were not detected between OSKL-iPSCs and OSKLH-iPSCs, but OSKLH-iPSCs tended to be relatively more aggregated in the transcriptome principal component analysis (PCA) than OSKL-iPSCs (Figure S1A). From this result, we hypothesized that the OSKLH-iPSCs may have less variation in gene expression among clones. To test this hypothesis, we calculated the number of genes with mean absolute error (MAE) of >2.0 in expression among clones in the same group, which we defined as high variability. Interestingly, the OSKLH-iPSCs had approximately half as many genes with high MAE as the OSKL-iPSCs, indicating that OSKLH-iPSCs has less variation in transcriptome than OSKL-iPSCs (Figure S1B). Then, we investigated Gene Ontology (GO) terms for the 1,350 genes with MAE >2.0 genes among the OSKL-iPSCs and found that many terms were related to gene expression (Figure S1C). We also compared the variability of DNA methylation with the same method and obtained results similar to transcriptome (Figure S1D). These analyses indicated that *H1FOO-DD* contributes to the generation of primed iPSC clones with less variation in gene expression and DNA methylation.

Next, we compared the trilineage differentiation potency of these iPSC clones in adherent culture using the STEMdiff Trilineage Differentiation Kit (Figure S1E) and compared their gene expression using Scorecard analysis (Tsankov et al., 2015), which examines the expression of 96 genes for each clone. There was no great difference between the two groups in ectoderm and mesoderm, but OSKLH-iPSCs showed a trend toward uniformly higher scores in endoderm, which is the most difficult differentiation to induce in this system (Figure S1F). Furthermore, we attempted terminal differentiation of the mesodermal lineage into cardiomyocytes. After 6 trials for each clone, we noted that OSKLH-iPSCs differentiated into cardiomyocytes with higher expression of TNNT2 (Figures S1G–S1I). In summary, OSKLH-iPSCs showed significantly higher potential for cardiomyocyte differentiation and slightly higher potential endoderm differentiation compared to OSKL-iPSCs.

Single-cell RNA-seq analysis reveals improved reprogramming of OSKLH-infected cells

We next investigated the mechanism of OSKLH-mediated improvement of reprogramming using single-cell RNA-seq (scRNA-seq) analysis. We sampled control HDFs, HDFs at day 5 and 15 after infection of SeV-OSKL or SeV-OSKLH, and at early passage (P2) of generated iPSCs. Uniform manifold approximation and projection (UMAP) plots revealed that on day 5, cells in the reprogramming process were classified into three clusters (Figure 2A). On day 15, cells in the reprogramming process were divided into five clusters, and PSCs into five clusters. Comparing the contribution of OSKL- or OSKLH-infected HDFs to each cluster, the highest numbers of OSKL and OSKLH cells on day 5 were in clusters #4 and #6, respectively, and on day 15, the highest numbers of OSKL and OSKLH cells were in clusters #8 and #11, respectively (Figures 2B, and S2A). We then extracted DEGs among these clusters and visualized them in feature plot (Figure 2C), dot plot (Figure 2D), and heatmap (Figure S2B). According to these

Figure 1. H1FOO-DD enhances reprogramming into primed pluripotency

(A) qPCR analysis of *H1FOO* expression in HDFs, HDFs during reprogramming and H9 ESCs. Data are shown as the mean ± SD. $n = 3$. ND: not determined.

(B) Schematic structure of the SeV-H1FOO vector and the modified vectors. We created vectors in which DD is added to the 5′ side (H1FOO-DD) or 3′ side (DD-H1FOO) of H1FOO for this study.

(C) Representative phase-contrast image and immunofluorescent staining for H1FOO of HDFs at day 5 post SeV-H1FOO-DD infection. Scale bar, 50 μm.

(D) Protein expression analysis of H1FOO-DD with Shield1 or without Shield1 by western blotting. Two days after the SeV vectors infection, cell culture temperature was raised from 35°C to 37°C to remove the SeV vectors. Three days after raising the temperature, 1 μM of Shield1 was added and the cells were collected the next day. We quantified the expression level of H1FOO-DD with GAPDH protein expression. Data are shown as the mean ± SD. $n = 3$. $^*p < 0.05$.

(E) Sequential protein expression analysis of H1FOO and H1FOO-DD by western blotting after the SeV-H1FOO or SeV-H1FOO-DD vector infection. We quantified the expression level of H1FOO and H1FOO-DD with GAPDH protein expression. Data are shown as the mean ± SD. $n = 3$. $^*p < 0.05$, $^{***}p < 0.001$.

(F) Number of alkaline phosphatase (AP)-positive primed human iPSC colonies generated from HDFs at day 14. Each of the linker histone H1 related vectors were co-infected with SeV-OSKL. Data are shown as the mean ± SD. $n = 3$. $^*p < 0.05$.

(G) Number of AP-positive primed human iPSC colonies generated from HDFs and PBMC at day 14. We used CytoTune-EX-iPS vector for reprogramming in this experiment. Data are shown as the mean ± SD. $n = 3$. $^*p < 0.05$.

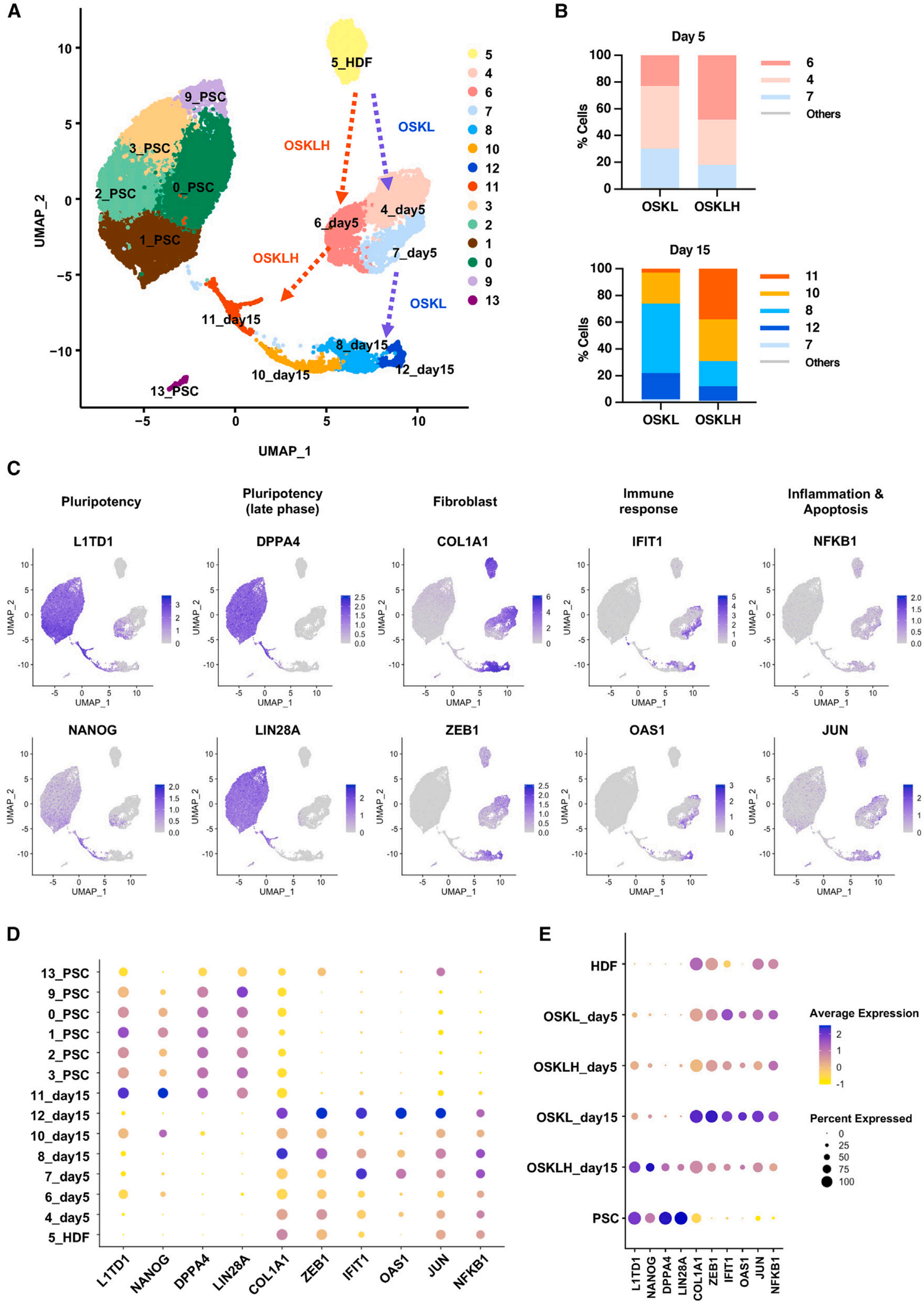

(legend on next page)

plots, on day 5, cluster #6 had the highest expression of pluripotency markers and suppressed expression of fibroblast-related genes, innate immune responses associated with general viral infection, and apoptosis markers. Conversely, cluster #7 showed higher expression of immune response and apoptosis markers. On day 15, cluster #8 was similar to cluster #7 in gene expression pattern. Cluster #11 showed not only a high level of pluripotency markers which are expressed in the late phase of reprogramming but also high expression of naive pluripotency, epithelial, and mesoendodermal markers which are transiently upregulated during iPSC generation (Takahashi et al., 2014) (Figure S2C). Next, we detected DEGs among clusters #6 and #7 on day 5 and clusters #11 and #12 on day 15, which are considered to be the cell groups with the most contrasting characteristics, and analyzed their GO terms (Table S1). This showed that the innate immune response, which is associated with viral infection, was suppressed, and cell proliferation was enhanced in cluster #6 compared to cluster #7. In addition, cell proliferation and stem cell maintenance-related genes were upregulated in cluster #11 compared to cluster #12, while the expression of genes related to innate immune response, inflammatory response, and apoptosis was suppressed in cluster #11. It is known that exogenous single-stranded RNA, such as the SeV vector genome used in this study, activates innate immunity in mammalian cells via interferon- and NF-κB-dependent pathways, which can impair reprogramming (Warren et al., 2010). These results suggested clusters #6, #10, and #11 were in the process of successful reprogramming, while clusters #8 and #12 were in the process of incomplete or unsuccessful reprogramming. There were more OSKLH-infected cells belonging to clusters #6, #10, and #11 than there were OSKL-infected cells. Finally, we compared the expression of representative groups of genes characterizing clusters in each cell groups (Figure 2E). Especially on day 15, OSKLH-infected cells showed expression of pluripotency markers more similar to PSCs than OSKL-infected cells, whereas OSKL-infected cells were notable for fibroblast, innate immunity, inflammation, and apoptosis markers. These results indicated that a higher percentage of OSKLH-infected cells were in the process of successful reprogramming.

OSKLH enhances chromatin opening and transcription factor binding around pluripotency markers

Linker histone H1 is known as a chromatin remodeling factor, and somatic H1, which exists ubiquitously in the body, functions to aggregate chromatin to form heterochromatin, whereas H1FOO functions to form relatively open chromatin (Ooga et al., 2016; Saeki et al., 2005). Given this role, we hypothesized that the *H1FOO-DD*-mediated increase in reprogramming efficiency may be due to H1FOO selectively opening chromatin regions important for the early stages of reprogramming. To test this hypothesis, we performed single-cell assay for transposase-accessible chromatin using sequencing (scATAC-seq) of HDFs infected with OSKL or OSKLH at day 2, day 5, and day 15, and as well as PSCs (H9 ESC). When we calculated the percentage of reads counted within the detected peak region (peak±500 bp), we observed a clear tendency for PSCs to have a lower ratio of reads within the peak compared to HDFs (Figure S3A). In addition, a comparison of OSKL- and OSKLH-infected cells showed that OSKLH cells had a higher ratio of reads in the peak region similar to PSCs, especially on day 15, implying that many OSKLH-introduced cells were transitioning to a nuclear state similar to that of PSCs. We next projected all the obtained peaks onto the UMAP, which resulted in 16 clusters (Figure 3A). We created plots projecting only OSKL- or OSKLH-infected cells onto UMAP, as well as tabulating the percentage of them belonging to each cluster by post-infection day (Figures 3B and S3B). On day 2, there was no significant difference in the distribution of cells in the clusters between the OSKL and OSKLH groups, but on day 5, most OSKL cells were in cluster #1, whereas most OSKLH cells were in cluster #8, and based on their position on the UMAP and the open region profile of transcription start site, cluster #8 was closer to the PSC clusters than cluster #1. On day 15, the majority of OSKL cells were in cluster #7, whereas OSKLH cells had a particularly large population of cells in clusters #9 and #14, and only OSKLH cells had some population in cluster #16. We then examined the specific characteristics of each cluster by gene activity analysis. Since the gene bodies and promoter regions of expressed genes are open chromatin, we predicted the gene expression activity of the cells using tags in the respective gene regions

Figure 2. Single-cell RNA-seq analysis of the reprogramming process to iPSCs

(A) UMAP of single-cell RNA-seq analysis. Dashed arrows indicate the reprogramming process inferred to be followed by the majority of SeV-OSKL- or SeV-OSKLH-infected HDFs.

(B) Percentage of cells in each cluster at day 5 and day 15 after SeV infection.

(C) Feature plot of representative marker gene expression in the UMAP.

(D) Dot plot of representative marker gene expression by cluster. The color of the dot indicates the average expression of the gene, and the size of the dot indicates the percentage of cells in which the gene is expressed.

(E) Dot plot of representative marker gene expression by cell type.

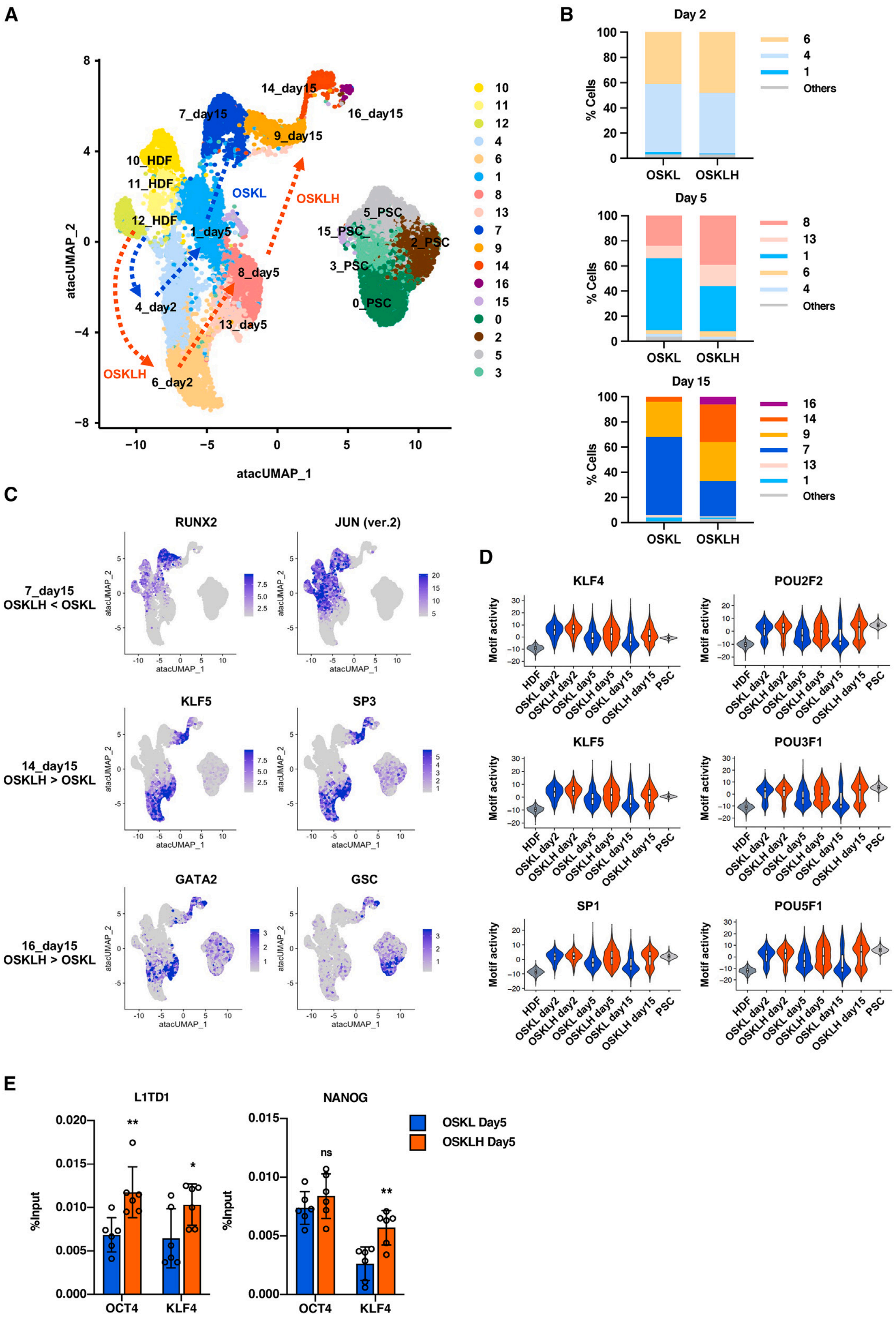

(legend on next page)

and 2 kb upstream. Based on this analysis, we extracted cells predicted to have high activity of specific gene expression in the UMAP (Figure S3C). We focused on pluripotency markers present at relatively early stages of reprogramming, such as *L1TD1* and *NANOG*, and found that they were expressed in clusters #4, #6, #8, #9, #13, and #14 and PSCs, while pluripotency markers mainly appearing late in reprogramming, such as *DPPA4* and *LIN28*, were strongly expressed in clusters #14 and #16 and PSCs. In summary, a larger proportion of OSKLH-infected cells changed to a PSC-like chromatin state, and the regions of the pluripotent marker genes were opened earlier in OSKLH-infected cells than in OSKL-infected cells.

In order to determine which transcription factors were activated in *H1FOO-DD*-mediated reprogramming, we performed motif analysis in each cluster based on the extracted peaks. We analyzed and listed top 10 motifs in clusters #1 and #7, which were dominated by OSKL cells, as well as in clusters #8, #14, and #16, which were dominated by OSKLH cells (Table S2). We then visualized the distribution of representative motifs in clusters #7, #14, and #16 with the feature plot (Figure 3C). Comparing clusters #1 and #8, #1 showed motifs of inflammation and apoptosis-related transcription factors at lower levels, while cluster #8 had many *POU* and *KLF* family motifs outside the top 10, in addition to transcription factors related to cell proliferation. In cluster #7, peak regions with *RUNX2/3*, *TEAD1-4*, and *AP-1* motifs were specifically detected, which are known as reprogramming inhibiting factors (Li et al., 2017). On the other hand, cluster #14 had *KLF/SP* family motifs, and cluster #16 had mesoendoderm lineage markers such as *GATA* family and *GSC*. We then extracted motifs that were significantly more abundant in OSKLH than in OSKL cells at each time point (Table S3, and Figure 3D). Interestingly, the *POU* and *KLF/SP* families were highly detected at all time points but especially on day 2, when motif activity of the *POU* family was significantly increased about 3-fold. In addition, the *KLF* family, especially *KLF2*, *4*, and *5*, had significantly higher motif activity at day 5 and day 15. To verify these analyses, we performed ChIP-qPCR of the day 5 HDFs after infection with SeV-OSKL or SeV-OSKLH using *OCT4* and *KLF 4* antibodies to detect *L1TD1* and *NANOG*. The results showed that OSKLH-infected cells had significantly higher levels of *L1TD1* occupying both *OCT4* and *KLF4*, and higher levels of *NANOG* occupying *KLF4* (Figure 3E). These results indicated that *H1FOO-DD* enhanced reprogramming by predominantly increasing the transcriptional activity of the *POU* and *KLF/SP* families from the early stage of reprogramming.

Finally, we integrated the gene activity data from scATAC-seq with the transcriptome data from scRNA-seq to investigate how well the results of the scATAC-seq and scRNA-seq analyses matched (Figure S3D). Interestingly, similar to the UMAP of scRNA-seq, the clusters of HDFs, day 5 cells, day 15 cells, and PSCs were obviously separated. Especially in day 15, cells were clearly distributed in four clusters as in scRNA-seq. Next, we classified the cells analyzed by scATAC-seq with the scRNA-seq-derived clusters and calculated the percentage in each group (Figure S3E). The day 2 OSKLH group had a lower percentage of cells in cluster #5 and a higher percentage of cells belonging to cluster #6, a group with a more suppressed immune response and advanced reprogramming, compared to day 2 OSKL cells of the same time course. This trend was more pronounced when comparing day 5 OSKL and OSKLH cells. At day 15, the OSKLH group accounted for nearly 30% of the most advanced reprogramming cells in cluster #11, while the OSKL group accounted for 4%, a difference of more than 7-fold. This difference was in general consistent with the difference in the number of iPSC colonies in the generation. These results showed that the cluster distribution of scATAC-seq was almost identical to that of scRNA-seq, and that reprogramming was more accelerated in OSKLH-introduced cells in terms of both transcriptome and chromatin accessibility.

FKBP1A suppresses innate immune responses and promotes reprogramming

In order to elucidate the mechanism by which *H1FOO-DD* enhances the speed and quality of reprogramming, we attempted to detect DEGs between OSKL- and OSKLH-infected HDFs in the early stages of reprogramming by bulk RNA-seq. We used three types of HDFs infected with OSKL or OSKLH and collected cells on day 1, day 2, and day 5 for bulk RNA-seq. In the day 2 samples, we detected 19 genes which were upregulated and 2 genes which were downregulated in OSKLH compared to OSKL (except

Figure 3. Single-cell ATAC-seq analysis of the reprogramming process to iPSCs

(A) UMAP of single-cell ATAC-seq analysis. Dashed arrows indicate the reprogramming process inferred to be followed by the majority of SeV-OSKL- or SeV-OSKLH-infected HDFs.

(B) Percentage of cells in each cluster at day 2, day 5, and day 15 after SeV infection.

(C) Feature plot of representative motif in #7, #14, and #16 in the UMAP.

(D) Violin plot of KLF/SP and POU family motif activity level in each cell group.

(E) ChIP-qPCR analysis of *L1TD1* and *NANOG* immunoprecipitated with OCT4 or KLF4. Data are shown as the mean ± SD. $n = 6$. $^{*}p < 0.05$, $^{**}p < 0.01$.

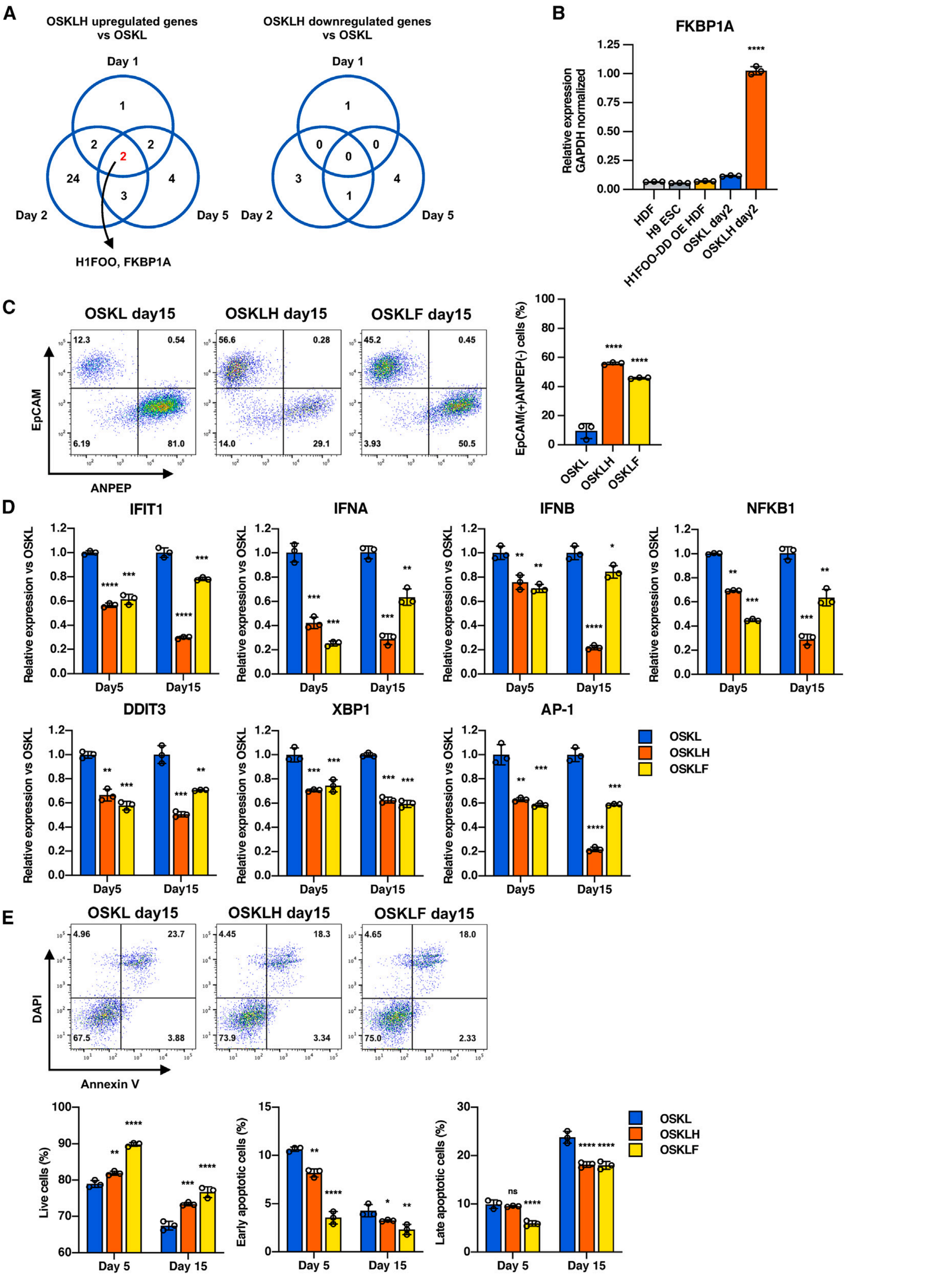

(legend on next page)

H1FOO; Figure S4A). Next, to evaluate the role of each of these genes in reprogramming efficiency, we overexpressed them or knocked them down individually in dermal fibroblasts with the PiggyBac system or with shRNA, and then infected with SeV-OSKL to generate primed and naive iPSCs. Overexpression of *FKBP1A* and *APOE* significantly improved both primed and naive reprogramming (Figure S4B). *APOE* has been reported to be highly expressed during the reprogramming process (Tanabe et al., 2013), and *H1FOO* and *FKBP1A* were the two DEGs commonly detected on day 1, day 2, and day 5 in all three types of HDFs (Figure 4A). Based on these results, we hypothesized that *FKBP1A* plays a key role in *H1FOO-DD*-mediated improved reprogramming.

Next, we examined the expression of *FKBP1A* by qPCR at day 2, when SeV-*H1FOO-DD* was introduced alone or together with OSKL to investigate how *FKBP1A* expression changes when *H1FOO-DD* is overexpressed in the beginning of reprogramming. Infection of HDFs with SeV-H1FOO-DD alone did not upregulate *FKBP1A* expression compared to HDFs or PSCs, whereas infection with SeV-OSKL upregulated *FKBP1A* expression about 2-fold. However, *H1FOO-DD* synergized with OSKL to greatly increase *FKBP1A* expression over 8-fold (Figure 4B).

Furthermore, we examined how *FKBP1A* expression changed during reprogramming using the scRNA-seq and the scATAC-seq data previously shown in Figures 2 and 3. We compared the chromatin status of the *FKBP1A* coding region in OSKL- and OSKLH-infected HDFs at day 2 and 5 in the scATAC-seq data and found no obvious differences in chromatin accessibility between the two groups (Figure S4C). Next, we examined the proportion of *FKBP1A*-positive cells and their expression levels in both groups using scRNA-seq data. Interestingly, although the percentage of *FKBP1A*-positive cells was not significantly different between the two groups, OSKLH cells showed markedly higher expression of the gene, especially on day 5 (Figure S4D). In addition, the distribution of cells with high *FKBP1A* expression (Figure S4E) and cells with high *H1FOO* expression (Figure S4F) was almost identical. These results suggest that although the chromatin accessibility of the *FKBP1A* coding region was not significantly different between the OSKL and OSKLH groups, *FKBP1A* transcription was markedly activated in the presence of OSKLH.

FKBP1A is an immunophilin protein known to be involved in immunosuppression, and it binds to TGFBR1 and suppresses its phosphorylation, thereby inhibiting the activity of downstream signaling pathways (Chen et al., 1997; Yamaguchi et al., 2006). Moreover, inhibition of TGFBR1 phosphorylation (pTGFBR1) is known to promote mesenchymal-to-epithelial transition (MET) and improve reprogramming efficiency (Ruetz et al., 2017). We hypothesized that *FKBP1A* plays a central role in the mechanism of the enhanced reprogramming effect of H1FOO-DD by suppressing innate immunity and promoting MET. To test this hypothesis, we used HDFs with overexpression of OSKL and *FKBP1A* (OSKLF). In our examination, this combination of factors achieved the same degree of suppression of pTGFBR1 as OSKLH (Figure S4G). Next, to test whether *FKBP1A* promotes MET during the reprogramming process, we performed flow cytometry of OSKLF-overexpressing HDFs to measure the expression of MET marker EpCAM and the fibroblast marker ANPEP on day 15. Although OSKLF-infected cells did not achieve the level of EpCAM+ANPEP− cells observed in the OSKLH condition, the degree of EpCAM+ANPEP− epithelial transition was much greater than when OSKL was expressed alone (Figure 4C).

To examine whether *FKBP1A* enhances the suppression of innate immune responses during reprogramming, we sampled OSKLF-overexpressing HDFs on day 5 and day 15 to examine their innate immune responses and expression of apoptosis markers. OSKLF and OSKLH significantly suppressed the expression of these markers compared to OSKL (Figure 4D). Finally, we performed an apoptosis assay using AnnexinV to compare the inhibition of apoptosis during the reprogramming process and found that OSKLF inhibited apoptosis as well as OSKLH on both day 5 and day 15 (Figure 4E). These results indicate that the expression of *FKBP1A* is strongly driven by OSKLH, which promotes MET and enhances reprogramming efficiency by suppressing innate immune responses, inflammation, and apoptosis.

Figure 4. FKBP1A suppresses innate immune responses and promotes reprogramming

(A) Upregulated and downregulated DEGs of bulk RNA-seq analysis comparing SeV-OSKL-infected group to SeV-OSKLH-infected group at day 1, 2, and 5.

(B) qPCR analysis of FKBP1A expression in PSC and several conditions of HDF. Data are shown as the mean ± SD. $n = 3$. ****$p < 0.0001$.

(C) Quantification of EpCAM and ANPEP expression in HDFs during reprogramming at day 15 by flow cytometry. Data are shown as the mean ± SD. $n = 3$. ****$p < 0.0001$.

(D) qPCR analysis of innate immune response-related marker gene expression during reprogramming at day 5 and day 15. Data are shown as the mean ± SD. $n = 3$. *$p < 0.05$, **$p < 0.01$, ***$p < 0.001$, ****$p < 0.0001$.

(E) Quantification of Annexin V and DAPI expression in HDFs during reprogramming at day 5 and day 15 by flow cytometry. Data are shown as the mean ± SD. $n = 3$. *$p < 0.05$, **$p < 0.01$, ***$p < 0.001$, ****$p < 0.0001$.

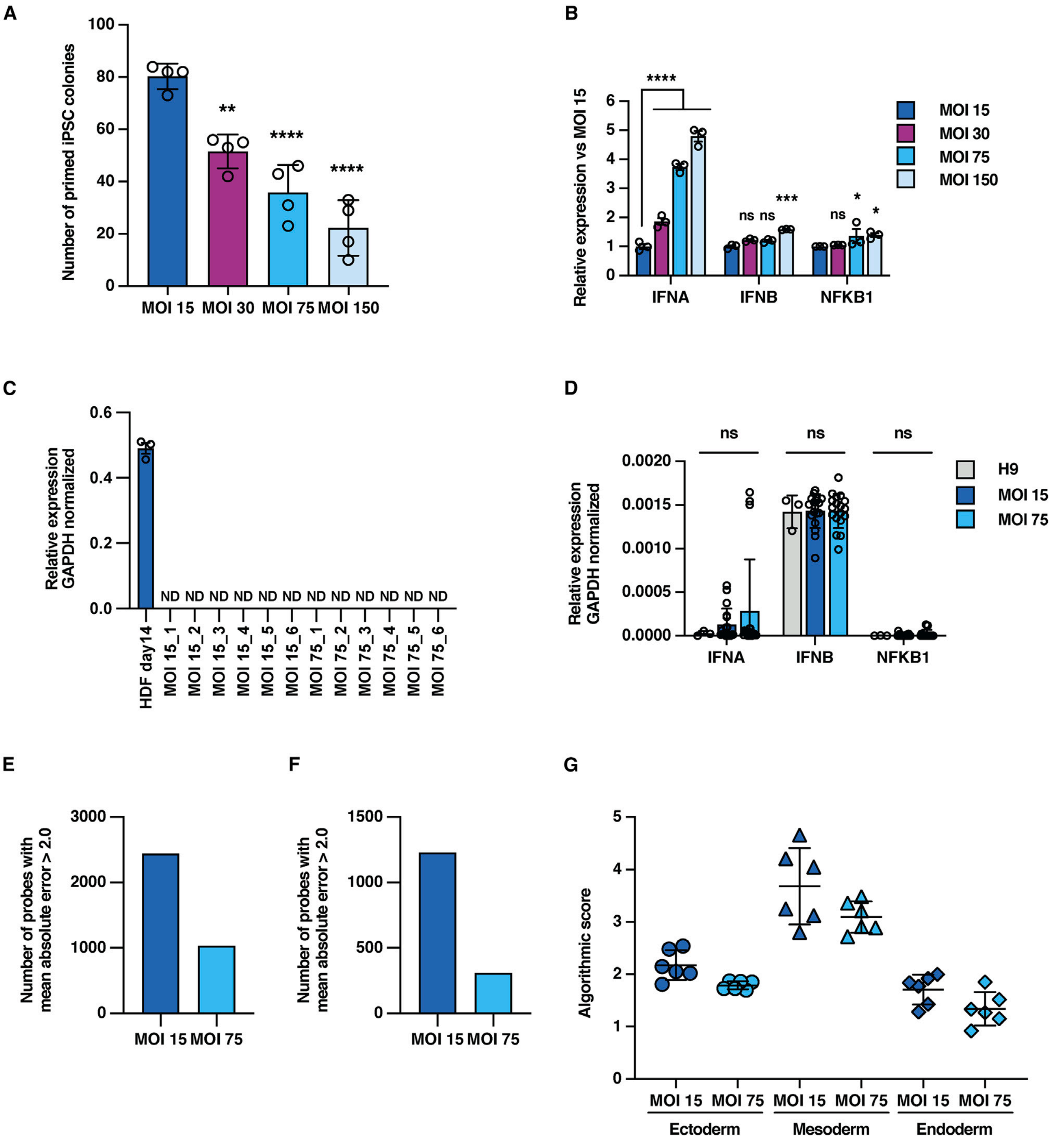

Figure 5. Excessive innate immune response deteriorates reprogramming

(A) Number of AP-positive primed human iPSC colonies generated from HDFs at day 14. Each HDF was infected with SeV-Mock in addition to OSKL MOI = 15 except for MOI 15 condition. Data are shown as the mean ± SD. $n = 4$. $^{**}p < 0.01$, $^{****}p < 0.0001$.

(B) qPCR analysis of innate immune response-related inflammation marker gene expression in each condition at day 14. Data are shown as the mean ± SD. $n = 3$. $^{*}p < 0.05$, $^{***}p < 0.001$, $^{****}p < 0.0001$.

(C) qPCR analysis of SeV genome expression in generated iPSC clones at p20 (day 160). Data are shown as the mean ± SD. $n = 3$ for each clone. ND, not detected even after 40 amplification cycles.

(legend continued on next page)

Excessive innate immune response leads to irreversible selection of iPSCs with low differentiation potency

The activation of the innate immune response we observed in the beginning of reprogramming is considered to be related to viral infection. It has been reported that the transient inflammation caused by overexpression of genes related to innate immune response in iPSCs reduces their differentiation ability and increases heterogeneity among iPSC clones in the short term (Eggenberger et al., 2019). However, the comparison of differentiation potency between OSKL-iPSCs and OSKLH-iPSCs was performed at p20, when the innate immune response is completely suppressed, and we observed that OSKLH-iPSC tended to show better potential to differentiate into some lineages. Therefore, to verify the hypothesis that innate immunosuppression by *H1FOO-DD* and *FKBP1A* contributed to the enhanced differentiation potency of generated iPSCs, we examined the effect of excessive innate immune activation on reprogramming, including in iPSCs that were maintained for many passages. SeV-OSKL is usually used at a total MOI = 15 to generate iPSCs, but to amplify the immune response we infected HDFs not only with OSKL MOI = 15, but also with SeV-Mock MOI = 15, 60, and 135 simultaneously, so that the total MOI = 30, 75, and 150, respectively. Reprogramming efficiency decreased relative to the total MOI (Figure 5A). Comparing the innate immune responses by qPCR showed that the expression of *IFNA* and *NFkB1* increased significantly with increasing MOI (Figure 5B).

When SeV-OSKL and SeV-OSKLH were compared, the difference in their iPSC colony generation efficiency and *IFNA* expression level was generally about 3-fold, similar to the difference between MOI = 15 and MOI = 75. Therefore, we compared the cell characteristics between iPSCs generated by infection at a total MOI = 15 (MOI 15-iPSCs) as a control group and at a total MOI = 75 (MOI 75-iPSCs) as representative of excessive innate immune activation. We established six clones each of MOI 15-iPSCs and MOI 75-iPSCs that were negative for the SeV genome (Figure 5C), and the transcriptome and DNA methylome were compared at p20, a remote stage corresponding to day 160 post SeV infection, when markers related to the innate immune response had converged to the equivalent of H9 ESCs (Figure 5D). Interestingly, MOI 75-iPSCs had about half the number of genes with high variation in expression with MAE>2.0 compared to MOI 15-iPSCs (Figure 5E), and the number of methylated probes meeting the same criteria was reduced to about one-quarter (Figure 5F). Next, we differentiated the iPSCs using the Trilineage Differentiation Kit at p20 and compared the expression levels of the marker genes for each lineage using the Scorecard. MOI 75-iPSCs had markedly lower differentiation ability in all lineages compared to MOI 15-iPSCs, especially in the ectoderm and mesoderm lineages, and showed less variability (Figure 5G). In summary, excessive SeV infection caused a stronger innate immune response which decreased in the efficiency of iPSC generation, but the variation in the transcriptome and DNA methylome of the generated iPSCs was relatively suppressed. Moreover, a marked decrease in differentiation capacity was observed even at the remote stage after SeV infection, when the innate immune response was considered to have ended. These results indicate that the innate immune response to excessive SeV vector in the early stages of reprogramming not only reduces the efficiency of iPSC generation, but also has the effect of irreversible selection of iPSCs with low differentiation potency.

H1FOO-DD enhances reprogramming into naive pluripotency

The scRNA-seq and scATAC-seq analyses showed that SeV-OSKLH-infected HDFs transiently had a significantly larger group of cells with naive state marker expression. Therefore, we hypothesized that H1FOO-DD may have a positive effect on reprogramming to the naive state, which requires a higher level of pluripotency. To examine this hypothesis, we generated naive iPSCs from HDFs and PBMCs by infecting with SeV-OSKL (nOSKL-iPSCs) or SeV-OSKLH (nOSKLH-iPSCs) using naive iPSC culture media under on-feeding and hypoxia conditions (Kunitomi et al., 2022). There was no significant difference in the morphology of naive iPSCs generated by infection with either SeV-OSKL or SeV-OSKLH, and the typical dome-like shape of naive PSCs was observed, similar to mouse PSCs (Figure 6A). However, SeV-OSKLH induced significantly more efficient generation of iPSC colonies and regardless of the original cell type, as was the case with primed iPSCs (Figure 6B).

Next, we generated 6 clones each of nOSKL-iPSCs and nOSKLH-iPSCs and compared their transcriptome and methylome by bulk RNA-seq and DNA methylation arrays. As comparison, we included naive PSCs from the datasets of Reset H9 ESC (nH9) (Takashima et al., 2014), which were converted to naive state by overexpression

(D) qPCR analysis of innate immune response-related inflammation marker gene expression in each condition at p20 (day 160). Data are shown as the mean ± SD. $n = 3$ for each clone.

(E) Number of genes with MAE >2.0 in clones in the MOI 15 and MOI 75 groups in RNA-seq.

(F) Number of genes with MAE >2.0 in clones in the MOI 15 and MOI 75 groups in DNA methylation array.

(G) Dot plot of algorithmic scores generated by Scorecard analysis based on 96 genes expression per sample. $n = 1$ of each point.

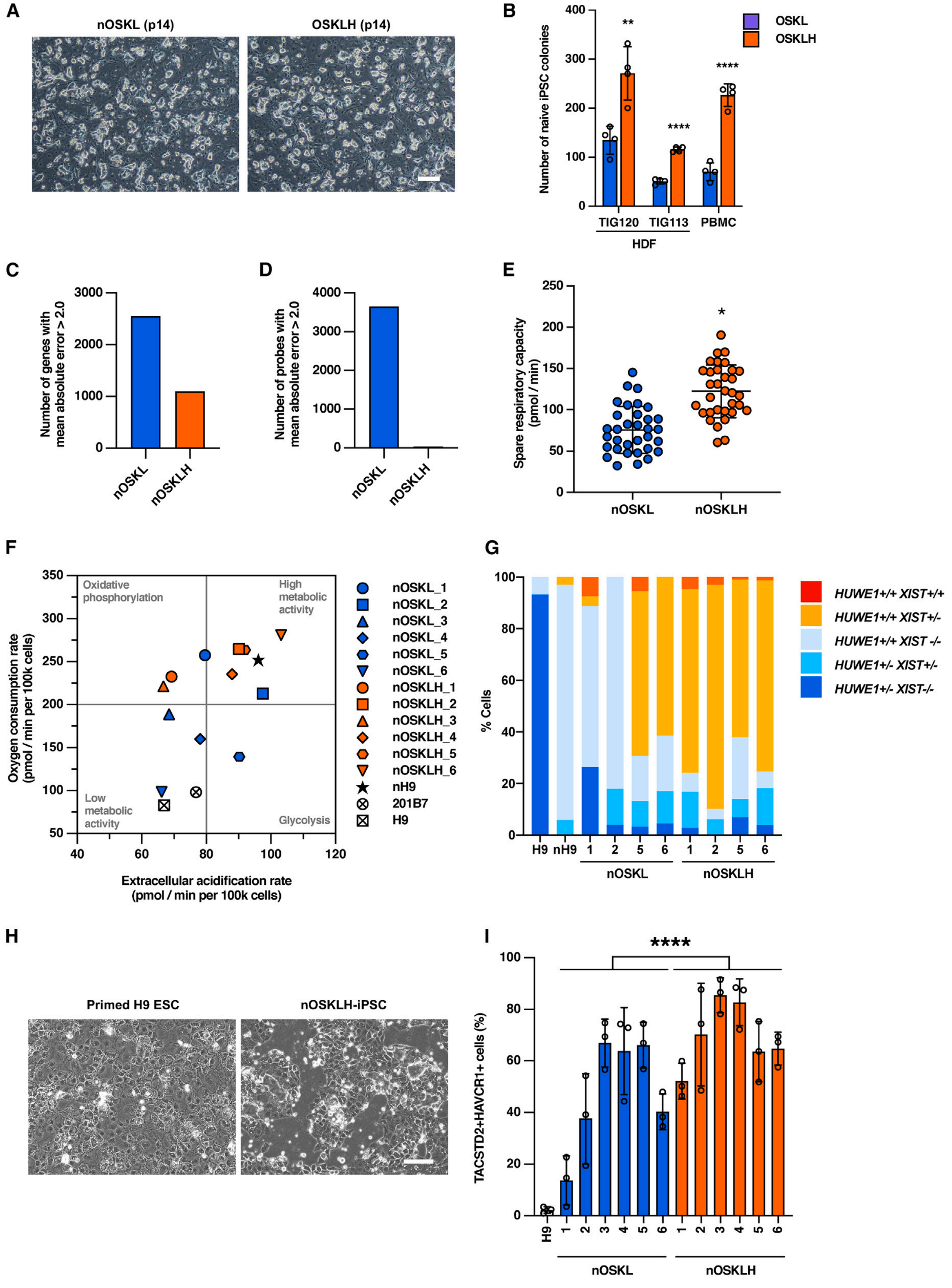

(legend on next page)

of *KLF2* and *NANOG*, 4i/5i/6iLA naive PSCs (Theunissen et al., 2014), and human blastocyst cells (Yan et al., 2013). In PCA, both nOSKL-iPSCs and nOSKLH-iPSCs were located in almost the same area as nH9 cells, clearly distinguishing them from primed PSCs (Figure S5A). Moreover, there was no significant difference in expression of pluripotency markers between nOSKL-iPSCs and nOSKLH-iPSCs (Figure S5B), and no DEGs were found between nOSKL-iPSCs and nOSKLH-iPSCs. Next, we examined the number of genes that showed MAE >2.0 among the clones. As with the primed iPSCs, nOSKLH-iPSCs had less than half as many genes with MAE >2.0 in expression compared to nOSKL-iPSCs (Figure 6C). DNA methylation analysis showed that all naive PSCs were markedly demethylated compared to primed PSCs, and in PCA, naive PSCs formed a distinct group from primed PSCs generated from the same cells (Figures S5C and S5D). When we examined the number of probes with the MAE >2.0 in DNA methylation in the same manner as in Figure 6C, remarkably, nOSKL-iPSCs had more than double the number of genes with MAE >2 compared to their primed counterparts, whereas variability in methylation in nOSKLH-iPSCs was suppressed to only 29 probes (Figure 6D). In summary, OSKLH-iPSCs showed a more uniform trend in transcriptome and DNA methylation than OSKL-iPSCs, especially in DNA methylation, even in the naive state.

Compared to primed PSCs, naive PSCs are known to have enhanced aerobic and anaerobic metabolism. We compared the expression of oxidative phosphorylation, glycolytic system, and tricarboxylic acid cycle-related genes in nOSKL and nOSKLH using bulk RNA-seq data but found no significant differences between the two groups (Figures S6A–S6C). We further examined the metabolic function of the generated naive iPSCs by monitoring the extracellular acidification rate (ECAR) and oxygen consumption rate (OCR) using a Seahorse flux analyzer (Figure S6D). nOSKLH-iPSCs showed clearly higher spare respiratory capacity than nOSKL-iPSCs, indicating higher aerobic metabolism (Figure 6E). Metabolic states defined by ECAR and OCR showed a trend toward a more energetic phenotype in nOSKLH-iPSCs than in nOSKL-iPSCs, and some nOSKLH-iPSC clones showed profiles similar to or higher than those of nH9 cells (Figure 6F). In summary, nOSKLH-iPSCs were more active in both aerobic metabolism and glycolysis than nOSKL-iPSCs and were reprogrammed to a level similar to more naive ESCs.

In principle, female-derived somatic cells and primed human PSCs have one X chromosome inactivated, but it is known that reprogramming to the naive state results in reactivation of the inactivated allele (Weinberger et al., 2016). The naive iPSCs generated in this study were all female-derived cells, so we performed RNA fluorescence *in situ* hybridization to examine the expression of the X chromosome gene *HUWE1* and the X chromosome inactivation factor *XIST*. To ensure that only normal cells with a diploid X chromosomes were included in the analysis, we only considered cells with biallelic expression of *UTX*, which escapes X chromosome inactivation. As previously reported, primed H9 ESCs were predominantly $HUWE1^{+/-}$, and Reset nH9 ESCs were almost all $HUWE1^{+/+}$, but few cells expressing *XIST* were observed (Sahakyan et al., 2017). All naive PSCs generated in this study were predominantly $HUWE1^{+/+}$ X-active cells. nOSKLH-iPSCs showed a large number of cells with an expression pattern very similar to that of preimplantation blastocysts, such as $HUWE1^{+/+}$, $XIST^{+/-}$ or $HUWE1^{+/+}$, and $XIST^{+/+}$ in all clones (Figure 6G). Therefore, nOSKLH-iPSCs more uniformly retained an X chromosome state more similar to preimplantation blastocyst than nH9 ESC or nOSKL-iPSCs.

Figure 6. H1FOO-DD enhances reprogramming into naive pluripotency

(A) Representative phase-contrast images of naive iPSCs reprogrammed with SeV-OSKL or SeV-OSKLH on iMEF feeder cells (p14). Scale bars, 200 μm.

(B) Comparison of AP-positive naive human iPSC colonies generated from HDFs or PBMCs at day 14. Data are shown as the mean ± SD. $n = 4$. $^{**}p < 0.01$, $^{****}p < 0.0001$.

(C) Number of genes with MAE >2.0 in clones of nOSKL-iPSCs and nOSKLH-iPSCs in RNA-seq.

(D) Number of genes with MAE >2.0 in clones of nOSKL-iPSCs and nOSKLH-iPSCs in DNA methylation array.

(E) Spare respiratory capacity of 6 clones each of nOSKL-iPSCs and nOSKLH-iPSCs analyzed by Seahorse. $n = 1$ of each point and $n = 6$ for each clone. $^{*}p < 0.05$.

(F) Metabolic profiles showing ECAR and OCR under FCCP-induced respiration measured at 50 min in the Seahorse analysis shown in Figure S5E. Each point shows the average of $n = 6$ for each clone.

(G) Quantification of the RNA fluorescence *in situ* hybridization patterns for *XIST* with *HUWE1* in cells with bi-allelic *UTX* expression. 100 cells were analyzed in each cell line.

(H) Representative phase-contrast images of primed H9 ESC and nOSKLH-iPSC-derived differentiated cells on day 3 after trophectoderm induction. Scale bars, 400 μm.

(I) Percentage of TACSTD2+HAVCR1+ cells differentiated from primed H9 ESCs (H9), nOSKL-iPSCs, and nOSKLH-iPSCs analyzed by flow cytometry. Data are shown as the mean ± SD. $n = 3$. $^{****}p < 0.0001$.

One of the main unresolved issues facing naive human PSCs is the apparent higher frequency of chromosomal aberrations than in primed PSCs (Theunissen and Jaenisch, 2014). We tested primed PSCs and naive PSCs by karyotyping to see if this problem is improved by H1FOO-DD (Table S4). Most of the primed iPSC clones showed no abnormalities, whereas the naive iPSC clones showed polyploidy in all clones as well as numerous other karyotypic abnormalities in some clones, similar to the Reset H9 ESCs and OSKL-iPSCs. Given the possibility that the t2iLGö+Y medium caused karyotypic aberrations, we also examined genomic aberrations of naive iPSCs generated in PXGLY (Bredenkamp et al., 2019) or AXGY (Khan et al., 2021) media (Table S5). We generated 3 clones of naive iPSCs each in each medium condition. For PXGLY medium, naive iPSCs were generated under the following two conditions: naive iPSCs generated using PXGLY medium only (clone name: PXGLY), and those generated using t2iLGö+Y medium until day 14 from the beginning of reprogramming, followed by using PXGLY medium to maintain the naive iPSCs (clone name: t2iLGö+Y to PXGLY). We were unable to generate naive iPSCs in AXGY alone, so we generated naive iPSCs using t2iLGö+Y or 5iLA for the first 14 days, and then changed the medium to AXGY medium (clone names: t2iLGö+Y→AXGY and 5iLA→AXGY). We then compared CNVs by SNP genotyping array (Figure S6E), and no abnormalities were detected in t2iLGö+Y medium or PXGLY medium. However, when AXGY medium was used, some abnormalities were detected in both nOSKL-iPSCs and nOSKLH-iPSCs, with slightly more abnormalities in the nOSKL-iPSC group (Table S5). Taken together, H1FOO-DD was found to have no apparent effect on suppressing genomic abnormality. In addition, there was no obvious indication that cells grown in t2iLGö+Y medium were more prone to genomic aberrations than those grown in PXGLY or AXGY.

Finally, to investigate the naive PSC-specific differentiation potency, we differentiated nOSKL-iPSCs and nOSKLH-iPSCs into naive-derived trophectoderm (nTE), which is difficult to induce differentiation from primed PSCs (Io et al., 2021). After a 3-day differentiation induction period, naive iPSCs differentiated into cells with morphology identical to previously reported naive PSC-derived differentiated cells, in obvious difference to primed PSCs (Figure 6H). The expression of TACSTD2 and HAVCR1, representative markers of nTE, was also examined using flow cytometry, and we found that there were almost no TACSTD2+HAVCR1+ cells in primed iPSCs, but naive iPSCs were able to differentiate to nTE, with nOSKLH-iPSCs showing a significantly better capacity than nOSKL-iPSCs (Figure 6I).

DISCUSSION

We found that addition of *H1FOO-DD*, human *H1FOO* with a destabilizing domain, enhanced both primed and naive reprogramming when overexpressed in human somatic cells for a short period with OSKL. *H1FOO-DD* promoted the chromatin accessibility and activity of the *POU* and *KLF/SP* families and suppressed the innate immune response in the early stages of reprogramming. In addition to enhancing reprogramming efficiency, *H1FOO-DD* also made the transcriptome and methylome more uniform among generated iPSC lines, and generated iPSCs with higher potential to differentiate to some lineages. Therefore, the addition of *H1FOO-DD* to iPSC reprogramming protocols is expected to advance research into human early development, disease modeling, drug discovery, and personalized cell therapy efforts.

Reprogramming is a phenomenon that involves drastic modification of the epigenome, so factors that modify the epigenome to recapitulate its early developmental state are very effective in reprogramming (Okita et al., 2007; Papp and Plath, 2013; Takahashi et al., 2007). H1FOO is involved in chromatin decondensation immediately after fertilization in mice, and is known to be rapidly downregulated after fertilization (Funaya et al., 2018; Tanaka et al., 2003). It has been reported to improve the reprogramming efficiency of mouse iPSCs and reduce the heterogeneity of differentiation potency among generated iPSC clones (Kunitomi et al., 2016). Based on these previous findings and our results of this study, we postulate that H1FOO-DD changes the wide range of chromatin in somatic cells to a chromatin more similar to that of the fertilized egg when it achieves totipotency and thereby facilitates faster and more efficient binding of reprogramming factors such as OCT4 and KLF4 to target genes. After reprogramming factors bind to the target regions and reprogramming begins, H1FOO-DD is rapidly degraded and thus does not interfere with the dynamically changing chromatin structure during the reprogramming process.

It has been reported in several previous studies that a strong and sustained innate immune response during the reprogramming process reduces reprogramming efficiency (Diebold et al., 2004; Warren et al., 2010). In support of this, our scRNA-seq and scATAC-seq analyses showed that innate immune responses were strongly persistent in cell clusters where reprogramming was considered unsuccessful. In addition, our study found that iPSCs generated during the process of excessive innate immune response showed more uniform transcriptome, methylome, and inferior differentiation potency even after the innate immune response had disappeared by long-term passaging. These results suggest that excessive innate immune

activation in the early stage of reprogramming forced uniformly low-quality reprogramming in SeV-infected cells, and this environment led to the selection of iPSCs with poor differentiation ability that just barely achieved reprogramming to the PSC level. Thus, it is crucial to minimize the innate immune response in the early stages of iPSC generation to ensure their function. *H1FOO-DD* suppressed innate immune responses, and we further showed that this occurred at least in part through upregulation of *FKBP1A*. scATAC-seq results showed that chromatin accessibility around *FKBP1A* was not altered by the presence of *H1FOO-DD*. This suggests that changes in chromatin structure, which is considered to be one of the major functions of H1FOO, may not be directly related to its effect on *FKBP1A* expression. This may also be one of the reasons why high expression of H1FOO-DD alone did not increase *FKBP1A* expression.

Notably, the naive iPSCs we generated in this study showed many karyotypic abnormalities after late passages, and H1FOO-DD did not suppress the formation of karyotypic abnormalities. Since the primed iPSCs did not show such pronounced abnormalities, differences in culture environment and pluripotency state are likely the main causes of karyotypic abnormalities. Further work is necessary to completely solve this problem.

EXPERIMENTAL PROCEDURES

Resource availability

Lead contact

Further information and requests for resources and reagents should be directed to and will be fulfilled by the lead contact, Akira Kunitomi: akira.kunitomi@gladstone.ucsf.edu.

Materials availability

All unique/stable reagents generated in this study, including SeV vectors, are available from the lead contact with a completed Materials Transfer Agreement.

Data and code availability

- Single-cell RNA-seq, single-cell ATAC-seq, bulk RNA-seq, DNA methylation array, and SNP genotyping array data are accessible in the Gene Expression Omnibus database of the National Center for Biotechnology Information website. The accession number is GSE224850.
- This paper does not report original code.
- Any additional information required to reanalyze the data reported in this paper is available from the lead contact upon request.

Experimental model and subject details

We obtained HDFs collected under informed consent from the Tokyo Metropolitan Institute of Gerontology (Kondo and Yonezawa, 1995). iMEFs were purchased from Thermo Fisher Scientific and PBMCs were purchased from Cellular Technology Limited. Primed and naive ESC clones were obtained from WiCELL (Thomson et al., 1998) and Kyoto University (Takashima et al., 2014). All cells except naive PSCs were cultured in humidified incubators at 37°C in 5% CO_2 and 20% O_2, and naive PSCs were cultured under 5% O_2 throughout. Recombinant DNA experiments in this study were carried out under the approval of Kyoto University and The J. David Gladstone Institutes.

Method details

Cell culture

HDFs and iMEFs were maintained in Dulbecco's modified Eagle's medium (DMEM, Nacalai Tesque) supplemented with 10% fetal bovine serum (FBS, Japan Bio Serum). PBMCs were cultured in StemSpan ACF (STEMCELL) with 100 ng/mL human SCF (R&D), 100 ng/mL human TPO (R&D), 100 ng/mL human Flt3/Flk2 (R&D), 50 ng/mL human IL-6 (R&D), and 20 ng/mL human IL-3 (R&D) for 5 days before the SeV vectors infection. Primed PSCs were maintained in StemFit AK02N medium (Ajinomoto) on laminin 511-E8 fragments (iMatrix-511, Nippi)-coated plates. Naive PSCs were cultured on iMEFs and maintained in t2iLGö (Takashima et al., 2014) medium composed of N2B27 medium (NDiff227, Takara Bio) with 1 μM CHIR99021 (Merck), 1 μM PD0325901 (Merck), 10 μg/mL human LIF (PeproTech), and 2.5 μM Gö6983 (Merck). The day before naive PSCs were plated, iMEF cells were seeded in cell culture dishes at a concentration of 25,000 cells/cm^2 and cultured overnight. The next day, the cells were washed twice with PBS(−) before plating. Medium was changed every other day and 10 μM Y27632 (Wako) was added just before every medium change. Primed iPSCs were passaged every 6–7 days, and naive iPSCs were every 3–4 days using Accutase (Innovative Cell Technologies).

Reprogramming of human somatic cells into primed iPSCs

We used CytoTune-iPS 2.0L or CytoTuneEX-iPS as SeV-OSKL vectors. HDFs were initially maintained in DMEM (Nacalai Tesque) supplemented with 10% FBS. PBMCs were cultured for 5 days in the StemSpan ACF-based medium described in the cell culture section. At day 0, the cells were counted and infected with three SeV vectors: (1) polycistronic *KLF4-OCT4-SOX2*, (2) *LMYC*, and (3) *KLF4* at a MOI of 5, respectively. *H1FOO-DD* was infected at an MOI of 0.3 for HDFs and at an MOI of 1 for PBMCs.

In HDFs, from day 1, the medium was changed every other day. At day 5, the cells were reseeded on laminin 511-E8 fragment-coated plates. The next day the medium was changed to StemFit AK02N medium. When iPSC colonies grew to the point that they were visible to the eye around day 20, the colonies were manually picked and cloned.

In PBMCs, at day 1, the cells were counted and reseeded on laminin 511-E8 fragment-coated plates with StemSpan ACF-based medium. Beginning on day 2 and then every other day, the same amount of StemFit AK02N medium was added as day 1. At day 8, the medium was completely changed to StemFit AK02N medium.

Reprogramming of human somatic cells into naive iPSCs

We used CytoTuneEX-iPS as SeV-OSKL vectors for generating naive iPSCs. HDFs and PBMCs were cultured and infected with SeV vectors as in primed iPSC generation. According to the previously reported protocol (Kunitomi et al., 2022), we started incubating cells in a 5% O_2 hypoxic incubator at 35°C from the time of infection.

In HDFs, we reseeded cells on iMEF feeder cells at day 5. From the next day, medium was changed to t2iLGö + Y27632 medium. Around day 14, we did the first passage of generated naive iPSCs, and the incubation temperature was changed to 38°C to remove the SeV vectors. After confirming removal of the SeV vectors, the temperature was changed to 37°C.

In PBMC reprogramming, we counted and reseeded cells on iMEF feeder cells with StemSpan ACF-based medium at day 1. Beginning on day 2 and then every other day, we gradually shifted the medium to t2iLGö + Y27632 medium as we did for primed iPSC generation.

Quantification and statistical analysis

All “n” in figure legends indicate the number of independent replicates from separate experiments. Error bars represent mean ± SD. Statistical significance was determined by two-tailed unpaired Student’s t test or one-way ANOVA using Prism software (GraphPad), and it was set at $p < 0.05$ indicated by asterisk.

SUPPLEMENTAL INFORMATION

Supplemental information can be found online at https://doi.org/10.1016/j.stemcr.2024.04.005.

ACKNOWLEDGMENTS

We are grateful to M. Iwasaki, K. Takahashi, and K. Tomoda for sharing data and discussions prior to publication, M. Saito, A. Niwa, and M. Nakagawa for providing valuable experimental equipment, T. Okubo for technical assistance in nTE differentiation, Yui Narita for technical assistance in cardiomyocyte differentiation, M. Oda and CiRA Foundation members for single-cell RNA-seq and ATAC-seq analysis, T. Tolpa for creating the graphical abstract, and K. Claiborn for critical reading of this manuscript. This work was supported by the Core Center for iPS Cell Research, Research Center Network for Realization of Regenerative Medicine, AMED under grant number JP21bm0104001; iPS Cell Research Fund; and JSPS KAKENHI under grant numbers 16K19429 and 18K15846. This work was also supported by the on-site laboratory initiative launched by Kyoto University, funding from Mr. Hiroshi Mikitani, Mr. Marc Benioff, the L. K. Whittier Foundation, the Roddenberry Foundation, Gladstone Institutes, the National Heart, Lung, and Blood Institute (NHLBI), and the National Institutes of Health (NIH) (U01-HL100406 and U01-HL098179).

AUTHOR CONTRIBUTIONS

A.K. designed and conceived this study, performed most of the experiments, and analyzed the data. R.H. and V.A. cultured cells and performed differentiation assays. M.O. performed and supported flow cytometry. N.S. instructed and supported metabolic analysis. T.M.K., M.N., and T.Y. analyzed the RNA-seq and DNA methylation array data. H.K. analyzed the scATAC-seq data. T.O. and Y.O. performed chromosome analysis. K.W. generated iPSCs for SNP genotyping array and M.N. analyzed the SNP genotyping array data. J.K., H.H., and K.K. developed and provided the SeV vectors. Y.T. and S. Yuasa provided and instructed experimental techniques. S.T. conducted cardiomyocyte differentiation. K.F., N.T., and S. Yamanaka supervised the project. A.K. wrote the manuscript.

DECLARATION OF INTERESTS

A.K. and K.F. are co-inventors on a patent describing the method for producing human iPSCs from somatic cells using H1FOO-DD. K.F. is a co-founder and CEO of Heartseed Inc., and S.T., S. Yuasa, and K.F. own equity in Heartseed Inc. S.T. is an advisor of Heartseed Inc. J.K. and H.H. are employees and K.K. is a board member of ID Pharma Co., Ltd., without compensation relating to this study. S. Yamanaka is a scientific advisor to iPS Academia Japan without salary.

Received: June 27, 2023
Revised: April 6, 2024
Accepted: April 8, 2024
Published: May 2, 2024

REFERENCES

Akifuji, C., Iwasaki, M., Kawahara, Y., Sakurai, C., Cheng, Y.S., Imai, T., and Nakagawa, M. (2021). MYCL promotes iPSC-like colony formation via MYC Box 0 and 2 domains. Sci. Rep. *11*, 24254.

Banaszynski, L.A., Chen, L.C., Maynard-Smith, L.A., Ooi, A.G.L., and Wandless, T.J. (2006). A rapid, reversible, and tunable method to regulate protein function in living cells using synthetic small molecules. Cell *126*, 995–1004.

Bredenkamp, N., Yang, J., Clarke, J., Stirparo, G.G., von Meyenn, F., Dietmann, S., Baker, D., Drummond, R., Ren, Y., Li, D., et al. (2019). Wnt Inhibition Facilitates RNA-Mediated Reprogramming of Human Somatic Cells to Naive Pluripotency. Stem Cell Rep. *13*, 1083–1098.

Chen, Y.G., Liu, F., and Massague, J. (1997). Mechanism of TGFbeta receptor inhibition by FKBP12. EMBO J. *16*, 3866–3876.

Diebold, S.S., Kaisho, T., Hemmi, H., Akira, S., and Reis e Sousa, C. (2004). Innate antiviral responses by means of TLR7-mediated recognition of single-stranded RNA. Science (New York, NY) *303*, 1529–1531.

Eggenberger, J., Blanco-Melo, D., Panis, M., Brennand, K.J., and tenOever, B.R. (2019). Type I interferon response impairs differentiation potential of pluripotent stem cells. Proc. Natl. Acad. Sci. USA *116*, 1384–1393.

Francesconi, M., Di Stefano, B., Berenguer, C., de Andrés-Aguayo, L., Plana-Carmona, M., Mendez-Lago, M., Guillaumet-Adkins, A., Rodriguez-Esteban, G., Gut, M., Gut, I.G., et al. (2019). Single cell RNA-seq identifies the origins of heterogeneity in efficient cell transdifferentiation and reprogramming. Elife *8*, e41627.

Funaya, S., Ooga, M., Suzuki, M.G., and Aoki, F. (2018). Linker histone H1FOO regulates the chromatin structure in mouse zygotes. FEBS Lett. *592*, 2414–2424.

Fusaki, N., Ban, H., Nishiyama, A., Saeki, K., and Hasegawa, M. (2009). Efficient induction of transgene-free human pluripotent stem cells using a vector based on Sendai virus, an RNA virus that does not integrate into the host genome. Proc. Jpn. Acad. Ser. B Phys. Biol. Sci. *85*, 348–362.

Io, S., Kabata, M., Iemura, Y., Semi, K., Morone, N., Minagawa, A., Wang, B., Okamoto, I., Nakamura, T., Kojima, Y., et al. (2021).

Capturing human trophoblast development with naive pluripotent stem cells in vitro. Cell Stem Cell *28*, 1023–1039.e13.

Khan, S.A., Park, K.M., Fischer, L.A., Dong, C., Lungjangwa, T., Jimenez, M., Casalena, D., Chew, B., Dietmann, S., Auld, D.S., et al. (2021). Probing the signaling requirements for naive human pluripotency by high-throughput chemical screening. Cell Rep. *35*, 109233.

Kilens, S., Meistermann, D., Moreno, D., Chariau, C., Gaignerie, A., Reignier, A., Lelièvre, Y., Casanova, M., Vallot, C., Nedellec, S., et al. (2018). Parallel derivation of isogenic human primed and naive induced pluripotent stem cells. Nat. Commun. *9*, 360.

Kondo, H., and Yonezawa, Y. (1995). Fetal-adult phenotype transition, in terms of the serum dependency and growth factor requirements, of human skin fibroblast migration. Exp. Cell Res. *220*, 501–504.

Kunitomi, A., Hirohata, R., Arreola, V., Osawa, M., Kato, T.M., Nomura, M., Kawaguchi, J., Hara, H., Kusano, K., Takashima, Y., et al. (2022). Improved Sendai viral system for reprogramming to naive pluripotency. Cell Rep. Methods *2*, 100317.

Kunitomi, A., Yuasa, S., Sugiyama, F., Saito, Y., Seki, T., Kusumoto, D., Kashimura, S., Takei, M., Tohyama, S., Hashimoto, H., et al. (2016). H1foo Has a Pivotal Role in Qualifying Induced Pluripotent Stem Cells. Stem Cell Rep. *6*, 825–833.

Li, D., Liu, J., Yang, X., Zhou, C., Guo, J., Wu, C., Qin, Y., Guo, L., He, J., Yu, S., et al. (2017). Chromatin Accessibility Dynamics during iPSC Reprogramming. Cell Stem Cell *21*, 819–833.e6.

Li, H.O., Zhu, Y.F., Asakawa, M., Kuma, H., Hirata, T., Ueda, Y., Lee, Y.S., Fukumura, M., Iida, A., Kato, A., et al. (2000). A cytoplasmic RNA vector derived from nontransmissible Sendai virus with efficient gene transfer and expression. J. Virol. *74*, 6564–6569.

Liu, G., David, B.T., Trawczynski, M., and Fessler, R.G. (2020). Advances in Pluripotent Stem Cells: History, Mechanisms, Technologies, and Applications. Stem Cell Rev. Rep. *16*, 3–32.

Liu, X., Nefzger, C.M., Rossello, F.J., Chen, J., Knaupp, A.S., Firas, J., Ford, E., Pflueger, J., Paynter, J.M., Chy, H.S., et al. (2017). Comprehensive characterization of distinct states of human naive pluripotency generated by reprogramming. Nat. Methods *14*, 1055–1062.

Liu, X., Tan, J.P., Schröder, J., Aberkane, A., Ouyang, J.F., Mohenska, M., Lim, S.M., Sun, Y.B.Y., Chen, J., Sun, G., et al. (2021). Modelling human blastocysts by reprogramming fibroblasts into iBlastoids. Nature *591*, 627–632.

Nakagawa, M., Koyanagi, M., Tanabe, K., Takahashi, K., Ichisaka, T., Aoi, T., Okita, K., Mochiduki, Y., Takizawa, N., and Yamanaka, S. (2008). Generation of induced pluripotent stem cells without Myc from mouse and human fibroblasts. Nat. Biotechnol. *26*, 101–106.

Nakagawa, M., Takizawa, N., Narita, M., Ichisaka, T., and Yamanaka, S. (2010). Promotion of direct reprogramming by transformation-deficient Myc. Proc. Natl. Acad. Sci. USA *107*, 14152–14157.

Nichols, J., and Smith, A. (2009). Naive and primed pluripotent states. Cell Stem Cell *4*, 487–492.

Okita, K., Ichisaka, T., and Yamanaka, S. (2007). Generation of germline-competent induced pluripotent stem cells. Nature *448*, 313–317.

Ooga, M., Fulka, H., Hashimoto, S., Suzuki, M.G., and Aoki, F. (2016). Analysis of chromatin structure in mouse preimplantation embryos by fluorescent recovery after photobleaching. Epigenetics *11*, 85–94.

Papp, B., and Plath, K. (2013). Epigenetics of reprogramming to induced pluripotency. Cell *152*, 1324–1343.

Ruetz, T., Pfisterer, U., Di Stefano, B., Ashmore, J., Beniazza, M., Tian, T.V., Kaemena, D.F., Tosti, L., Tan, W., Manning, J.R., et al. (2017). Constitutively Active SMAD2/3 Are Broad-Scope Potentiators of Transcription-Factor-Mediated Cellular Reprogramming. Cell Stem Cell *21*, 791–805.e9.

Saeki, H., Ohsumi, K., Aihara, H., Ito, T., Hirose, S., Ura, K., and Kaneda, Y. (2005). Linker histone variants control chromatin dynamics during early embryogenesis. Proc. Natl. Acad. Sci. USA *102*, 5697–5702.

Sahakyan, A., Kim, R., Chronis, C., Sabri, S., Bonora, G., Theunissen, T.W., Kuoy, E., Langerman, J., Clark, A.T., Jaenisch, R., and Plath, K. (2017). Human Naive Pluripotent Stem Cells Model X Chromosome Dampening and X Inactivation. Cell Stem Cell *20*, 87–101.

Takahashi, K., Okita, K., Nakagawa, M., and Yamanaka, S. (2007). Induction of pluripotent stem cells from fibroblast cultures. Nat. Protoc. *2*, 3081–3089.

Takahashi, K., Tanabe, K., Ohnuki, M., Narita, M., Sasaki, A., Yamamoto, M., Nakamura, M., Sutou, K., Osafune, K., and Yamanaka, S. (2014). Induction of pluripotency in human somatic cells via a transient state resembling primitive streak-like mesendoderm. Nat. Commun. *5*, 3678.

Takashima, Y., Guo, G., Loos, R., Nichols, J., Ficz, G., Krueger, F., Oxley, D., Santos, F., Clarke, J., Mansfield, W., et al. (2014). Resetting transcription factor control circuitry toward ground-state pluripotency in human. Cell *158*, 1254–1269.

Tanabe, K., Nakamura, M., Narita, M., Takahashi, K., and Yamanaka, S. (2013). Maturation, not initiation, is the major roadblock during reprogramming toward pluripotency from human fibroblasts. Proc. Natl. Acad. Sci. USA *110*, 12172–12179.

Tanaka, M., Hennebold, J.D., Macfarlane, J., and Adashi, E.Y. (2001). A mammalian oocyte-specific linker histone gene H1oo: homology with the genes for the oocyte-specific cleavage stage histone (cs-H1) of sea urchin and the B4/H1M histone of the frog. Development (Cambridge, England) *128*, 655–664.

Tanaka, M., Kihara, M., Meczekalski, B., King, G.J., and Adashi, E.Y. (2003). H1oo: a pre-embryonic H1 linker histone in search of a function. Mol. Cell. Endocrinol. *202*, 5–9.

Theunissen, T.W., and Jaenisch, R. (2014). Molecular control of induced pluripotency. Cell Stem Cell *14*, 720–734.

Theunissen, T.W., Powell, B.E., Wang, H., Mitalipova, M., Faddah, D.A., Reddy, J., Fan, Z.P., Maetzel, D., Ganz, K., Shi, L., et al. (2014). Systematic identification of culture conditions for induction and maintenance of naive human pluripotency. Cell Stem Cell *15*, 524–526.

Thomson, J.A., Itskovitz-Eldor, J., Shapiro, S.S., Waknitz, M.A., Swiergiel, J.J., Marshall, V.S., and Jones, J.M. (1998). Embryonic stem cell lines derived from human blastocysts. Science *282*, 1145–1147.

Tsankov, A.M., Akopian, V., Pop, R., Chetty, S., Gifford, C.A., Daheron, L., Tsankova, N.M., and Meissner, A. (2015). A qPCR ScoreCard quantifies the differentiation potential of human pluripotent stem cells. Nat. Biotechnol. *33*, 1182–1192.

Warren, L., Manos, P.D., Ahfeldt, T., Loh, Y.H., Li, H., Lau, F., Ebina, W., Mandal, P.K., Smith, Z.D., Meissner, A., et al. (2010). Highly efficient reprogramming to pluripotency and directed differentiation of human cells with synthetic modified mRNA. Cell Stem Cell *7*, 618–630.

Weinberger, L., Ayyash, M., Novershtern, N., and Hanna, J.H. (2016). Dynamic stem cell states: naive to primed pluripotency in rodents and humans. Nat. Rev. Mol. Cell Biol. *17*, 155–169.

Yamaguchi, T., Kurisaki, A., Yamakawa, N., Minakuchi, K., and Sugino, H. (2006). FKBP12 functions as an adaptor of the Smad7-Smurf1 complex on activin type I receptor. J. Mol. Endocrinol. *36*, 569–579.

Yamanaka, S. (2020). Pluripotent Stem Cell-Based Cell Therapy-Promise and Challenges. Cell Stem Cell *27*, 523–531.

Yan, L., Yang, M., Guo, H., Yang, L., Wu, J., Li, R., Liu, P., Lian, Y., Zheng, X., Yan, J., et al. (2013). Single-cell RNA-Seq profiling of human preimplantation embryos and embryonic stem cells. Nat. Struct. Mol. Biol. *20*, 1131–1139.

Yu, L., Wei, Y., Duan, J., Schmitz, D.A., Sakurai, M., Wang, L., Wang, K., Zhao, S., Hon, G.C., and Wu, J. (2021). Blastocyst-like structures generated from human pluripotent stem cells. Nature *591*, 620–626.

Zhang, Z., Hong, Y., Xiang, D., Zhu, P., Wu, E., Li, W., Mosenson, J., and Wu, W.S. (2015). MicroRNA-302/367 cluster governs hESC self-renewal by dually regulating cell cycle and apoptosis pathways. Stem Cell Rep. *4*, 645–657.

Stem Cell Reports, Volume *19*

Supplemental Information

H1FOO-DD promotes efficiency and uniformity in reprogramming to naive pluripotency

Akira Kunitomi, Ryoko Hirohata, Mitsujiro Osawa, Kaho Washizu, Vanessa Arreola, Norikazu Saiki, Tomoaki M. Kato, Masaki Nomura, Haruko Kunitomi, Tokiko Ohkame, Yusuke Ohkame, Jitsutaro Kawaguchi, Hiroto Hara, Kohji Kusano, Takuya Yamamoto, Yasuhiro Takashima, Shugo Tohyama, Shinsuke Yuasa, Keiichi Fukuda, Naoko Takasu, and Shinya Yamanaka

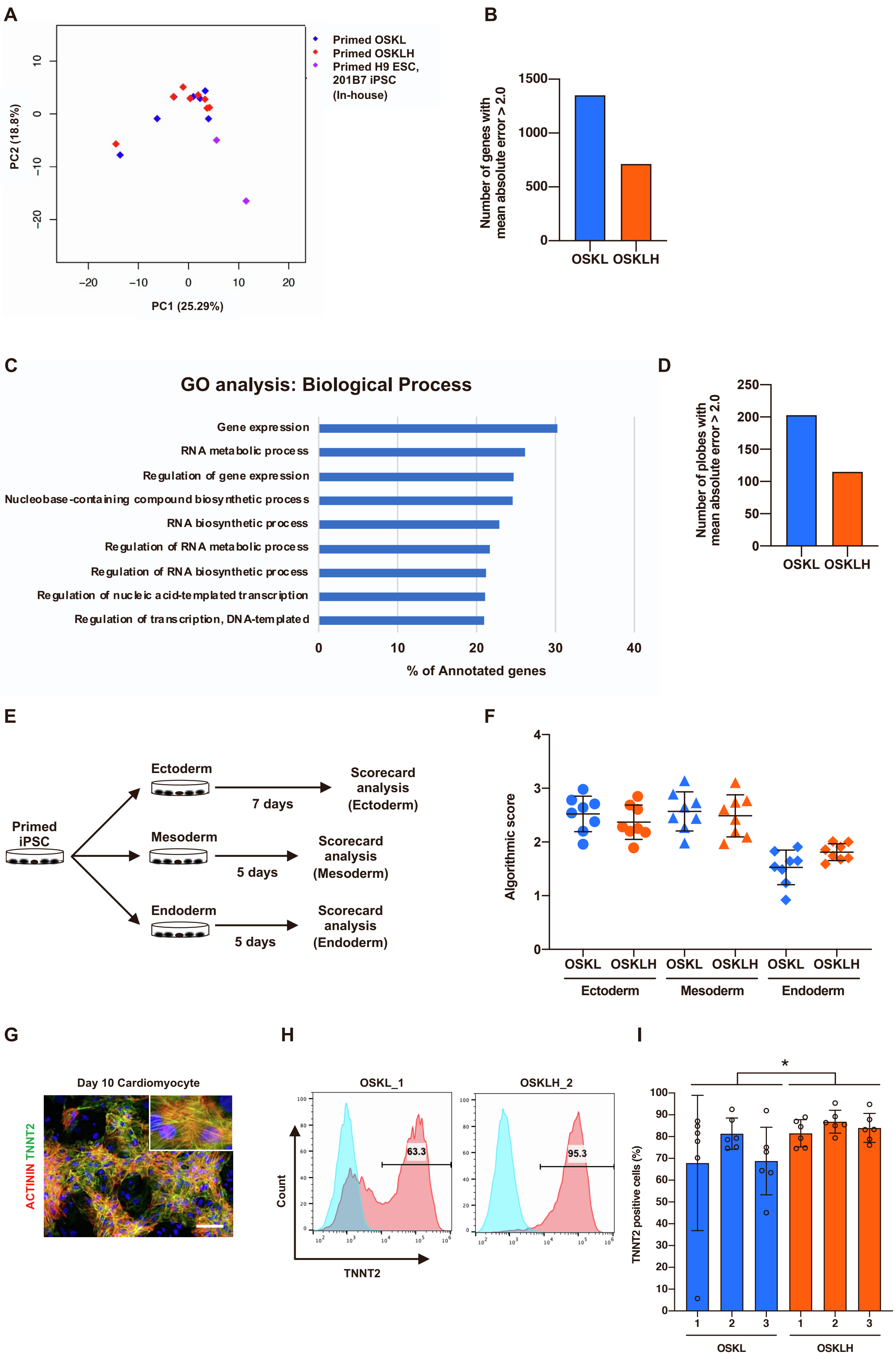

A
PC2 (18.8%)
PC1 (25.29%)
Primed OSKL
Primed OSKLH
Primed H9 ESC, 201B7 iPSC (In-house)
B
Number of genes with mean absolute error > 2.0
OSKL OSKLH
C
GO analysis: Biological Process
Gene expression
RNA metabolic process
Regulation of gene expression
Nucleobase-containing compound biosynthetic process
RNA biosynthetic process
Regulation of RNA metabolic process
Regulation of RNA biosynthetic process
Regulation of nucleic acid-templated transcription
Regulation of transcription, DNA-templated
% of Annotated genes
D
Number of plobes with mean absolute error > 2.0
OSKL OSKLH
E
Primed iPSC
Ectoderm
7 days
Scorecard analysis (Ectoderm)
Mesoderm
5 days
Scorecard analysis (Mesoderm)
Endoderm
5 days
Scorecard analysis (Endoderm)
F
Algorithmic score
OSKL OSKLH OSKL OSKLH OSKL OSKLH
Ectoderm
Mesoderm
Endoderm
G
Day 10 Cardiomyocyte
ACTININ TNNT2
H
OSKL_1
OSKLH_2
63.3
95.3
Count
TNNT2
I
TNNT2 positive cells (%)
OSKL
OSKLH

Figure S1 related to Figure 1. Characterization of H1FOO-DD vector

(A). PCA of RNA-seq data from primed human PSCs in this study compared to human PSCs.

(B). Number of genes with MAE >2.0 among the clones in each group in RNA-seq.

(C). GO terms for the 1350 genes with MAE >2.0 genes among the OSKL-iPSC clones.

(D). Number of probes with MAE >2.0 among the clones in each group in DNA methylation array.

(E). Schematic representation of the trilineage differentiation and analysis protocol.

(F). Dot plot of algorithmic scores generated by Scorecard analysis based on 96 genes expression per sample. *n*=1 of each point.

(G). Representative immunofluorescent staining for ACTININ and TNNT2 of cardiomyocytes at day 10 post differentiation from OSKLH-iPSCs. Scale bar, 100 µm.

(H). Quantification of TNNT2 expression in primed OSKL-iPSC and OSKLH-iPSC by flow cytometry.

(I). Percentage of TNNT2 expressed cells by flow cytometry. *n*=6 of each clone.

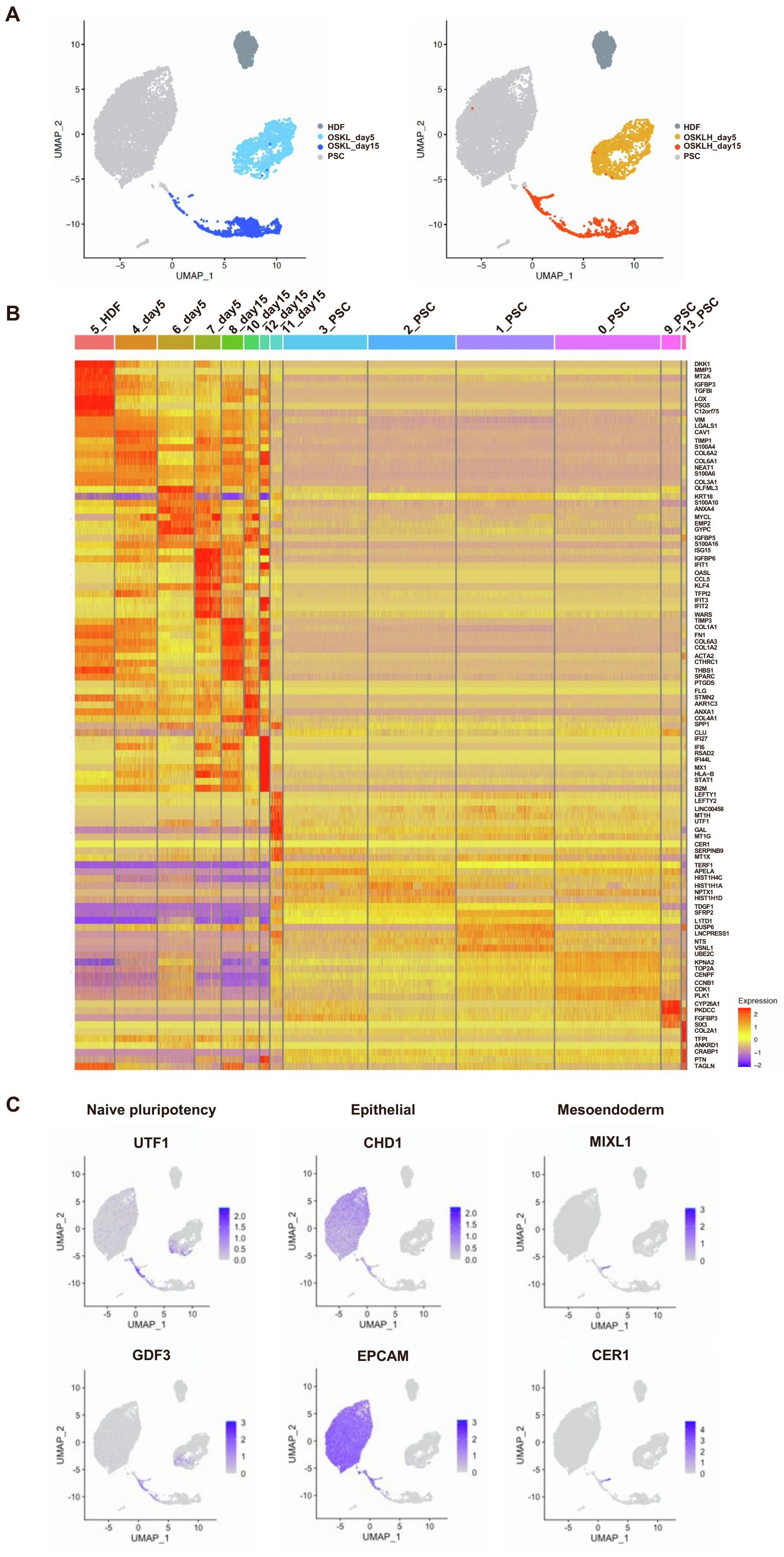

A
HDF
OSKL_day5
OSKL_day15
PSC
OSKLH_day5
OSKLH_day15
UMAP_1
UMAP_2
B
5_HDF
4_day5
6_day5
7_day5
8_day15
10_day15
12_day15
11_day15
3_PSC
2_PSC
1_PSC
0_PSC
9_PSC
13_PSC
DKK1
MMP3
MT2A
IGFBP3
TGFBI
LOX
PSG5
C12orf75
VIM
LGALS1
CAV1
TIMP1
S100A4
COL6A2
COL6A1
NEAT1
S100A6
COL3A1
OLFML3
KRT18
S100A10
ANXA4
MYCL
EMP2
GYPC
IGFBP5
S100A16
ISG15
IGFBP6
IFIT1
OASL
CCL5
KLF4
TFPI2
IFIT3
IFIT2
WARS
TIMP3
COL1A1
FN1
COL6A3
COL1A2
ACTA2
CTHRC1
THBS1
SPARC
PTGDS
FLG
STMN2
AKR1C3
ANXA1
COL4A1
SPP1
CLU
IFI27
IFI6
RSAD2
IFI44L
MX1
HLA−B
STAT1
B2M
LEFTY1
LEFTY2
LINC00458
MT1H
UTF1
GAL
MT1G
CER1
SERPINB9
MT1X
TERF1
APELA
HIST1H4C
HIST1H1A
NPTX1
HIST1H1D
TDGF1
SFRP2
L1TD1
DUSP6
LNCPRESS1
NTS
VSNL1
UBE2C
KPNA2
TOP2A
CENPF
CCNB1
CDK1
PLK1
CYP26A1
PKDCC
FGFBP3
SIX3
COL2A1
TFPI
ANKRD1
CRABP1
PTN
TAGLN
Expression
C
Naive pluripotency
Epithelial
Mesoendoderm
UTF1
CHD1
MIXL1
GDF3
EPCAM
CER1

Figure S2 related to Figure 2. Specific gene expression in each cell group and clusters in single cell RNA-seq analysis

(A). HDF, PSC and OSKL or HDF, PSC and OSKLH plotted UMAPs for comparing the difference in distribution between OSKL and OSKLH.

(B). Heatmap of top 10 representative marker genes expression in each cluster.

(C). Feature plot of naive pluripotency, epithelial and mesoendoderm related gene expression in the UMAP.

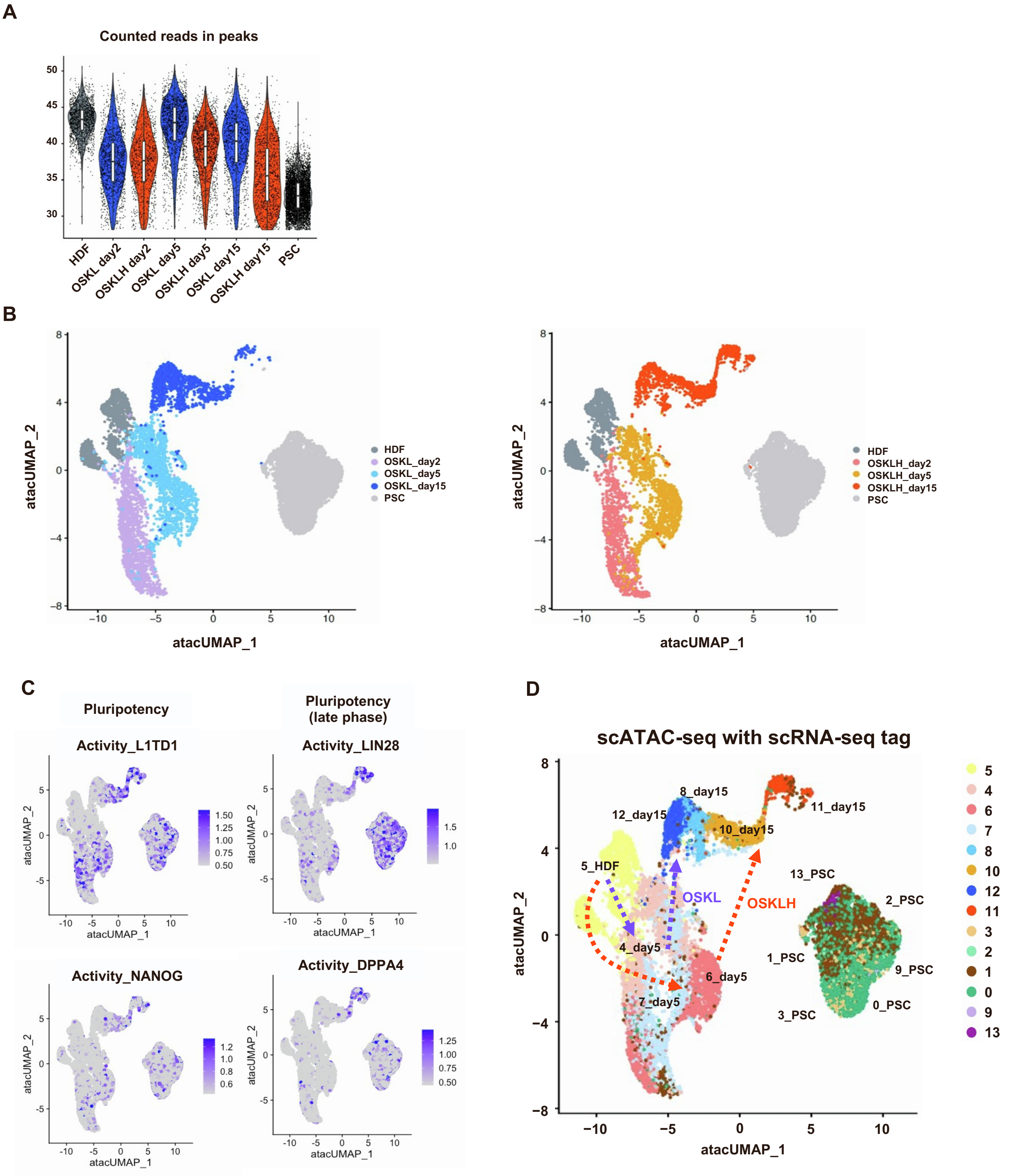

E

scRNA-seq derived clusters (columns); scATAC-seq ID (rows)

	5_HDF	4_day5	6_day5	7_day5	8_day15	10_day15	12_day15	11_day15	3_PSC	2_PSC	1_PSC	0_PSC	9_PSC	13_PSC	Total (%)
HDF_day0	97.9	1.3	0	0.5	0	0	0	0	0	0	0.3	0	0	0	100
OSKL_day2	8.4	26.9	16.1	34.3	0	0	0	0.2	0.4	0.4	8.9	4.4	0	0	100
OSKLH_day2	3.9	25.2	24.4	32.8	0	0	0	0	0.9	0.4	8.7	3.7	0	0	100
OSKL_day5	3.9	32.7	24.2	31.5	0.4	0	1.0	0	0.7	0	4.4	1.2	0	0	100
OSKLH_day5	0.3	28.4	46.5	19.7	0	0.1	0.5	0	0.4	0	3.5	0.8	0	0	100
OSKL_day15	0.9	3.5	0.3	2.7	26.5	24.9	34.7	4.0	0.2	0	1.9	0.3	0	0	100
OSKLH_day15	0.2	0.8	0.5	1.4	13.2	28.7	15.5	29.6	0.2	0	8.9	1.2	0	0	100
H9_ESC	0	0	0	0	0	0	0	0	12.1	6.7	31.3	45.7	1.5	2.7	100

Figure S3 related to Figure 3. Specific gene expression in each cell group and clusters in single cell RNA-seq analysis

(A). Percentage of reads counted within the detected peak region (peak±500 bp) in HDF, SeV-OSKL or SeV-OSKLH infected HDF and PSC.

(B). HDF, PSC and OSKL or HDF, PSC and OSKLH plotted UMAPs for comparing the difference in distribution between OSKL and OSKLH.

(C). Gene activity plot of pluripotency markers *L1TD1*, *NANOG*, *LIN28* and *DPPA4*. *LIN28* and *DPPA4* are known to be expressed from the late reprogramming stage.

(D). Plot of clusters produced by single cell RNA-seq on the single cell ATAC-seq UMAP by matching the gene activity data of single cell ATAC-seq and the gene expression data of single cell RNA-seq. Dashed arrows indicate the reprogramming process inferred to be followed by the majority of SeV-OSKL or SeV-OSKLH-infected HDFs.

(E). Percentage of cells in each cell group in single cell ATAC-seq that matched the gene expression patterns of the clusters created by single cell RNA-seq analysis.

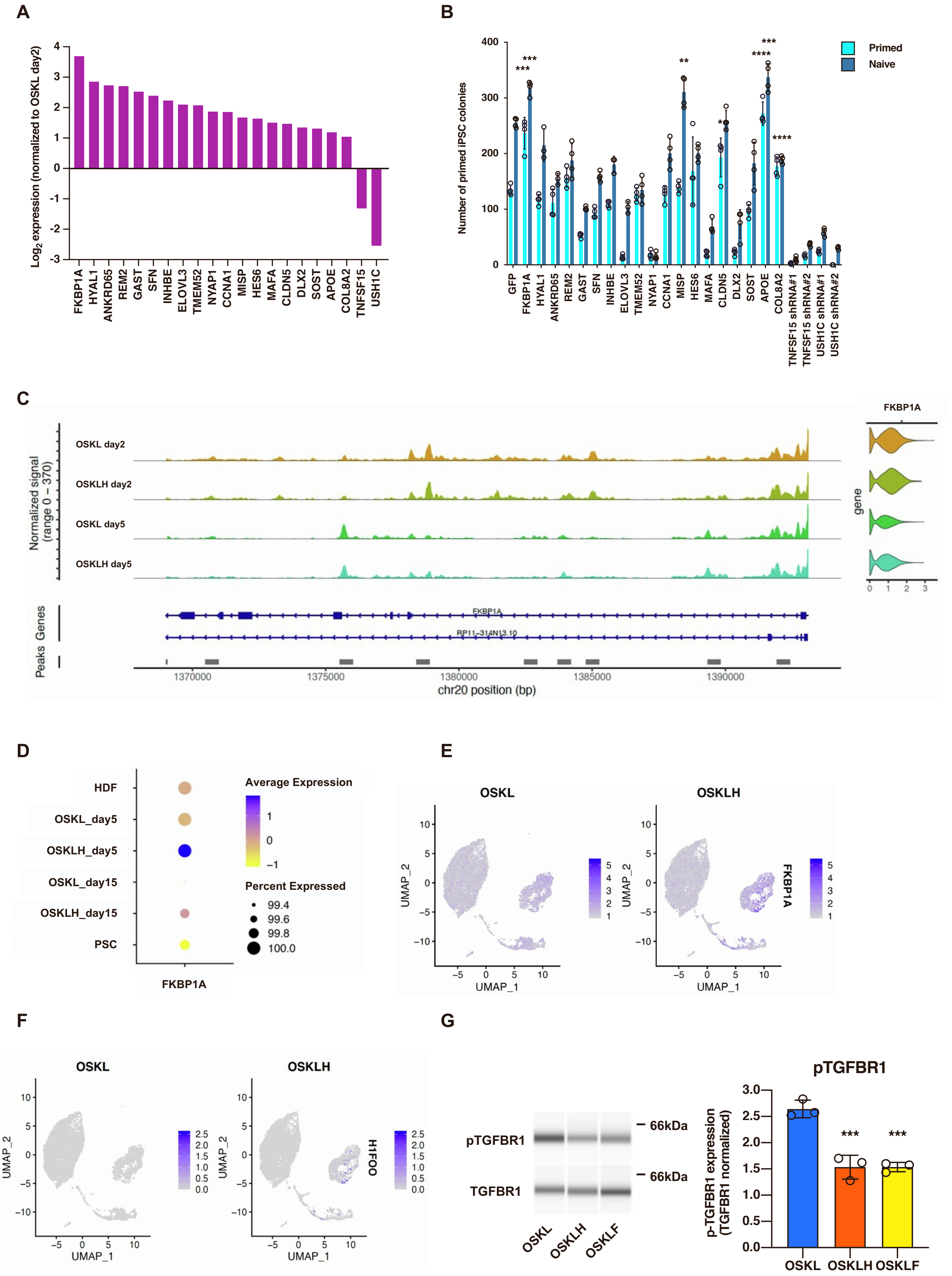

A
Log2 expression (normalized to OSKL day2)
FKBP1A HYAL1 ANKRD65 REM2 GAST SFN INHBE ELOVL3 TMEM52 NYAP1 CCNA1 MISP HES6 MAFA CLDN5 DLX2 SOST APOE COL8A2 TNFSF15 USH1C
B
Number of primed iPSC colonies
Primed
Naive
GFP FKBP1A HYAL1 ANKRD65 REM2 GAST SFN INHBE ELOVL3 NYAP1 CCNA1 MISP HES6 MAFA CLDN5 DLX2 SOST APOE COL8A2 TNFSF15 shRNA#1 TNFSF15 shRNA#2 USH1C shRNA#1 USH1C shRNA#2
C
Normalized signal (range 0 – 370)
OSKL day2
OSKLH day2
OSKL day5
OSKLH day5
Genes
Peaks
FKBP1A
RP11-314N13.10
chr20 position (bp)
FKBP1A
gene
D
HDF
OSKL_day5
OSKLH_day5
OSKL_day15
OSKLH_day15
PSC
FKBP1A
Average Expression
Percent Expressed
99.4
99.6
99.8
100.0
E
OSKL
OSKLH
UMAP_1
UMAP_2
FKBP1A
F
OSKL
OSKLH
UMAP_1
UMAP_2
H1FOO
G
pTGFBR1
TGFBR1
66kDa
OSKL
OSKLH
OSKLF
p-TGFBR1 expression (TGFBR1 normalized)
OSKL OSKLH OSKLF

Figure S4 related to Figure 4. Exploration of factors that play an important role in modified reprogramming by H1FOO-DD

(A). Representative DEGs detected in the bulk RNA-seq analysis.

(B). Number of primed and naive human iPSC colonies generated from HDFs at day 14. Data are shown as the mean ± s.d. n=3. *P<0.05.

(C). Coverage plot of *FKBP1A* coding region in OSKL and OSKLH at day 2 and day 5 obtained by single cell ATAC-seq.

(D). Dot plot of *FKBP1A* expression in OSKL and OSKLH in the UMAP obtained by single cell RNA-seq.

(E). Feature plot of *FKBP1A* expression in OSKL and OSKLH in the UMAP obtained by single cell RNA-seq.

(F). Feature plot of *H1FOO* expression in OSKL and OSKLH in the UMAP obtained by single cell RNA-seq.

(G). Protein expression analysis of phosphorylated TGFBR1 (pTGFBR1) and TGFBR1by Western blotting. We quantified the expression level of pTGFBR1 with TGFBR1 protein expression. Data are shown as the mean ± s.d. n=3. ***P<0.001.

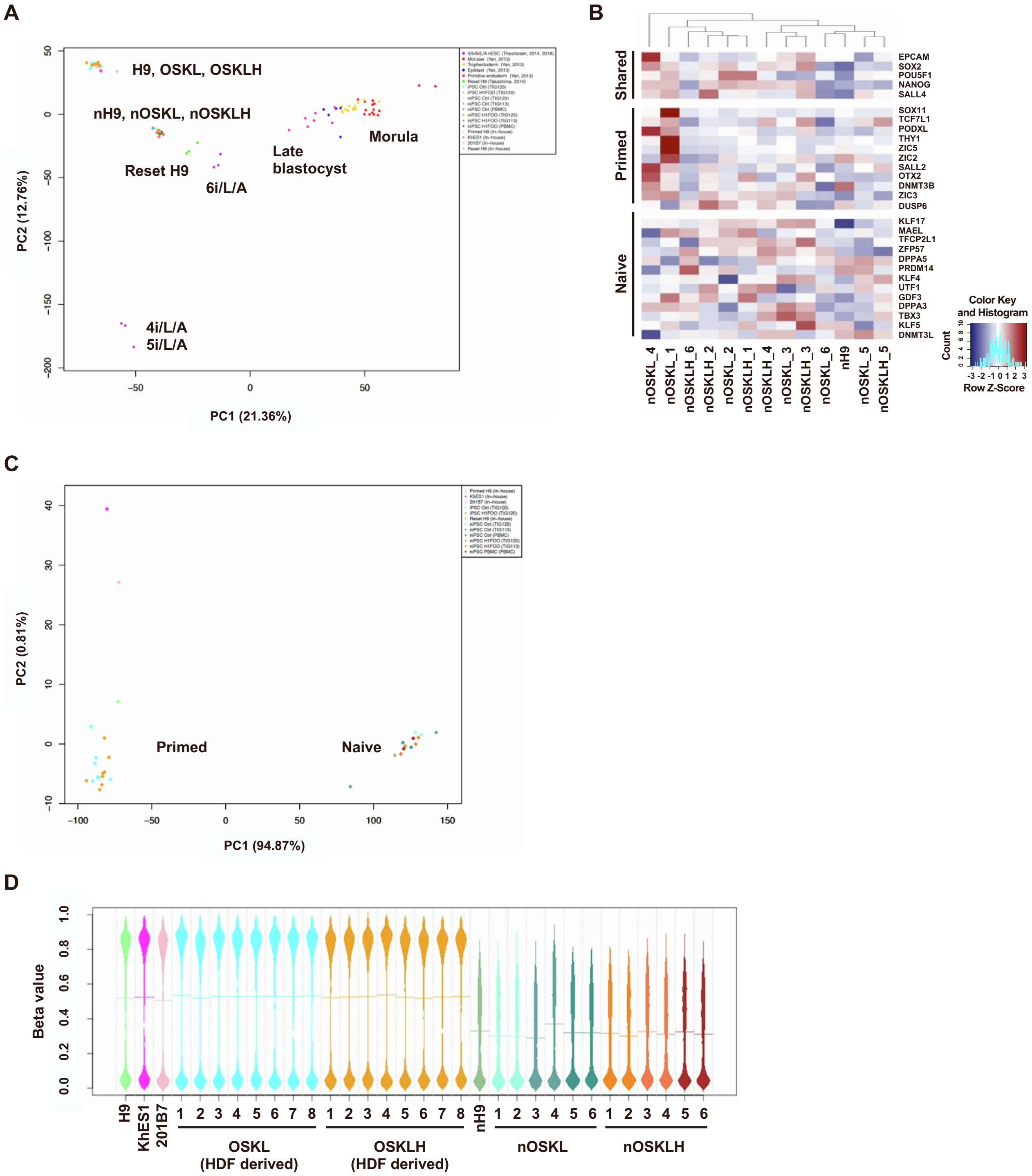

Figure S5 related to Figure 6. Exploration of factors that play an important role in modified reprogramming by H1FOO-DD

(A). PCA of RNA-seq data from primed and naive human PSCs in this study compared to reset naive human iPSCs and pre-implantation embryo samples from (Takashima et al., 2014; Theunissen et al., 2014; Yan et al., 2013).

(B) Heatmap of the RNA-seq data depicting expression levels of shared, primed, and naive

pluripotency-associated marker genes in naive PSCs.

(C). PCA of DNA methylation data from primed and naive human PSCs in this study.

(D). Beanplot of the global DNA methylation levels in primed and naive human PSCs analyzed using DNA methylation arrays. Horizontal lines in the beanplot represent mean methylation beta values.

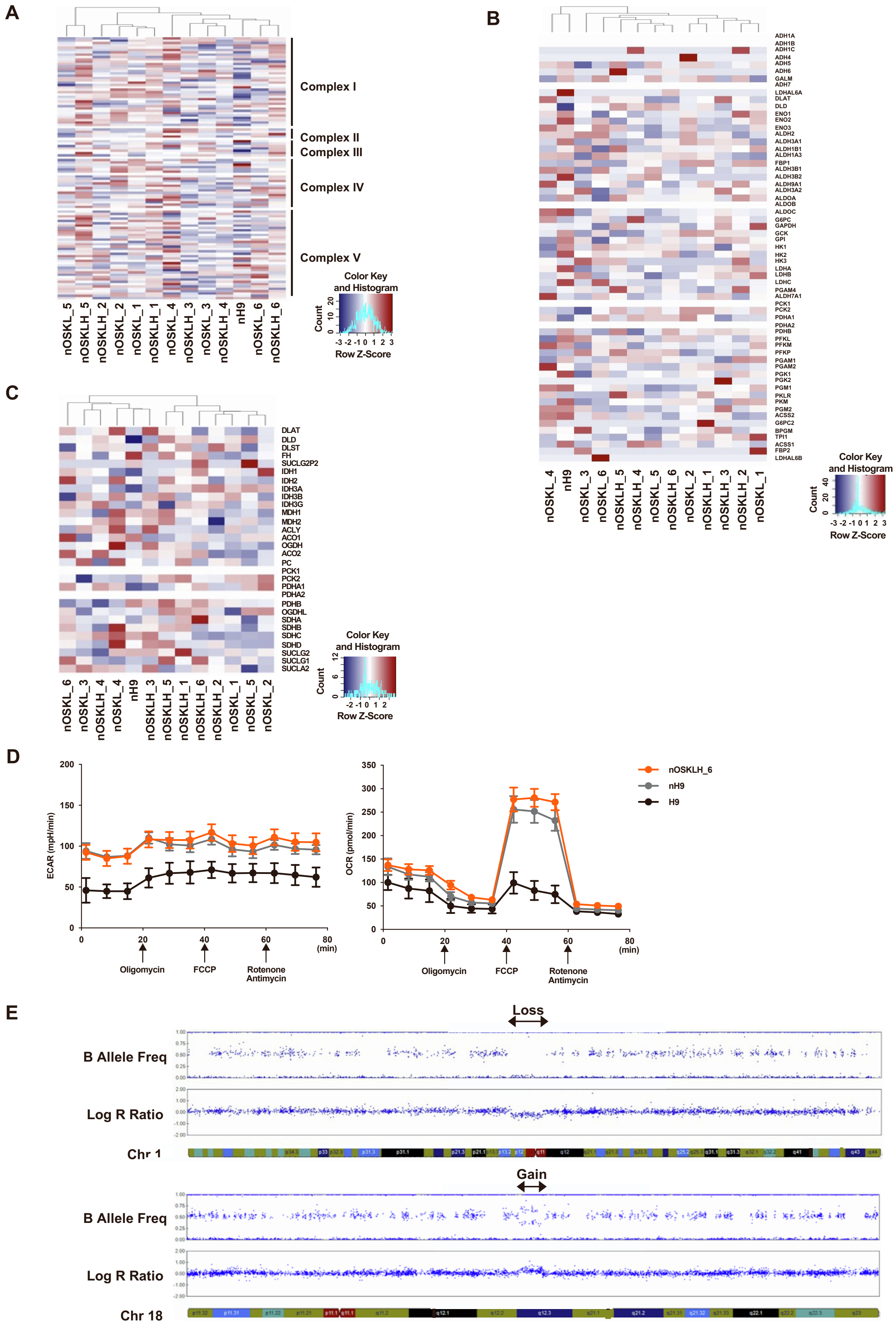
A
Complex I
Complex II
Complex III
Complex IV
Complex V
Color Key and Histogram
Count
Row Z-Score
nOSKL_5
nOSKLH_5
nOSKLH_2
nOSKL_2
nOSKL_1
nOSKLH_1
nOSKL_4
nOSKLH_3
nOSKL_3
nOSKLH_4
nH9
nOSKL_6
nOSKLH_6
B
ADH1A
ADH1B
ADH1C
ADH4
ADH5
ADH6
GALM
ADH7
LDHAL6A
DLAT
DLD
ENO1
ENO2
ENO3
ALDH2
ALDH3A1
ALDH1B1
ALDH1A3
FBP1
ALDH3B1
ALDH3B2
ALDH9A1
ALDH3A2
ALDOA
ALDOB
ALDOC
G6PC
GAPDH
GCK
GPI
HK1
HK2
HK3
LDHA
LDHB
LDHC
PGAM4
ALDH7A1
PCK1
PCK2
PDHA1
PDHA2
PDHB
PFKL
PFKM
PFKP
PGAM1
PGAM2
PGK1
PGK2
PGM1
PKLR
PKM
PGM2
ACSS2
G6PC2
BPGM
TPI1
ACSS1
FBP2
LDHAL6B
nOSKL_4
nH9
nOSKL_3
nOSKL_6
nOSKLH_5
nOSKLH_4
nOSKL_5
nOSKLH_6
nOSKL_2
nOSKLH_1
nOSKLH_3
nOSKLH_2
nOSKL_1
Color Key and Histogram
Count
Row Z-Score
C
DLAT
DLD
DLST
FH
SUCLG2P2
IDH1
IDH2
IDH3A
IDH3B
IDH3G
MDH1
MDH2
ACLY
ACO1
OGDH
ACO2
PC
PCK1
PCK2
PDHA1
PDHA2
PDHB
OGDHL
SDHA
SDHB
SDHC
SDHD
SUCLG2
SUCLG1
SUCLA2
nOSKL_6
nOSKL_3
nOSKLH_4
nOSKL_4
nH9
nOSKLH_3
nOSKLH_5
nOSKLH_1
nOSKLH_6
nOSKLH_2
nOSKL_1
nOSKL_5
nOSKL_2
Color Key and Histogram
Count
Row Z-Score
D
ECAR (mpH/min)
OCR (pmol/min)
(min)
Oligomycin
FCCP
Rotenone Antimycin
nOSKLH_6
nH9
H9
E
Loss
Gain
B Allele Freq
Log R Ratio
Chr 1
Chr 18

Figure S6 related to Figure 6. Examining the metabolic function and genomic aberrations of generated naive iPSCs

(A) Heatmap of genes encoding proteins of the electron transport chain located in the inner membrane of mitochondria. These genes reflect the activity of oxidative phosphorylation.

(B) Heatmap of genes encoding proteins of glycolytic system in naive PSCs.

(C) Heatmap of genes encoding proteins of TCA cycle in naive PSCs.

(D). Extracellular acidification rate (ECAR) and oxygen consumption rate (OCR) profiles measured by Seahorse of indicated cell lines. Oligomycin, FCCP and rotenone/antimycin were injected at indicated time points to evaluate mitochondrial capacity. n=6 for each point.

(E) Representative CNVs found in generated naive iPSCs: loss in Chr 1, and gain in Chr 18.

Table S1. GO terms and key genes of DEGs when comparing cluster #6 vs. #7 on day 5 and cluster #11 vs. #12 on day 15

	GO terms & Key Genes					
	down-regulated			up-regulated		
	GO terms	FDR	Key genes	GO terms	FDR	Key genes
#6 vs. #7	∽type I interferon signaling pathway	1.155E-17	*IFITM3, HLA-C, ISG15, IFIT1, OASL, IFI27*	～cell division	6.64E-06	*CENPV, UBE2C, TUBB, SMC1A, SMCA*
	～defense response to virus	1.787E-09	*RSAD2, ZC3HAV1, DDX60, IFIT2, HERC5*	～cell proliferation	3.26E-04	*CDV3, MCM7, CD70, PRKDC, TNFSF9*
	～extracellular matrix organization	1.748E-06	*FGF2, FN1, COL6A2, COL8A1, FBN1*	～nucleosome assembly	1.801.E-03	*NPM1, NASP, NAP1L1, ANP32B, HIST1H4C*
	～Immune response	2.824E-06	*IFI6, HLA-C, CCL5, CCL2, B2M*	∽cell-cell adhesion	8.802.E-03	*KRT18, RANBP1, CAST, CCNB1, FSCN1*
	～negative regulation of proliferation	1.303E-03	*MEG3, CXCL8, WARS, RARRES3, SOD2*	～mitotic nuclear division	1.945.E-02	*CENPF, CDC20, ASPM, CCNB2, BIRC5*
#11 vs. #12	～type I interferon signaling pathway	9.936E-26	*IFIT1, IFIT3, STAT1, HLA-B, OAS1, ISG20*	～translational initiation	1.950E-81	*RPSA, RPS3, RPS3a, RPL6, RPS25*
	～collagen catabolic process	1.020E-14	*COL1A1, COL1A2, CTSL, MMP1, MMP2*	～cell proliferation	2.504E-06	*MCM7, CKS1B, FRAT2, PRDX1, NANOG*
	～positive regulation of I-kappaB kinase/ NF-kappaB signaling	2.960E-06	*LGALS1, BST2, ECM1, SLC20A1, TRIM38*	∽somatic stem cell population maintenance	4.056E-04	*KLF4, POU5F1, SOX2, DPPA4, SALL4*
	∽positive regulation of fibroblast proliferation	1.276E-05	*JUN, ANXA2, WNT5A, FN1, S100A6*	～stem cell population maintenance	5.755E-03	*LIN28A, DPPA2, TET1, NANOG NODAL*
	～apoptotic process	1.775E-03	*SH3KBP1, PTEN, COMP, MYDGF, TNFSF10*	～negative regulation of apoptotic process	2.705E-02	*HDSPD1, EPCAM, KRT18, TDGF1, NPM1*

Table S2. Top 10 motifs in clusters #1, #7, #8, #14, and #16 based on the extracted peaks

1_day5
OSKLH < OSKL

motif	observed	background	percent.observed	percent.background	fold.enrichment	pvalue	motif.name
MA1123.2	352	23103	33.05164319	9.998009313	3.305822405	5.43E-94	**TWIST1**
MA1638.1	308	22443	28.92018779	9.712388998	2.977659544	6.28E-70	**HAND2**
MA0698.1	318	25652	29.85915493	11.10110959	2.689745082	2.70E-62	**ZBTB18**
MA0091.1	229	16105	21.50234742	6.969568454	3.0851763	7.72E-53	**TAL1::TCF3**
MA1468.1	159	10064	14.92957746	4.355277052	3.427928301	2.55E-41	**ATOH7**
MA1642.1	269	25244	25.25821596	10.92454431	2.312061287	1.42E-39	**NEUROG2(var.2)**
MA0835.2	335	36828	31.45539906	15.9376136	1.97365803	1.74E-36	**BATF3**
MA1634.1	341	38007	32.01877934	16.44783534	1.94668652	4.57E-36	**BATF**
MA0462.2	341	38036	32.01877934	16.46038533	1.945202297	5.33E-36	**BATF::JUN**
MA0490.2	292	30139	27.41784038	13.04289498	2.102128432	8.04E-36	**JUNB**

8_day5
OSKLH > OSKL

motif	observed	background	percent.observed	percent.background	fold.enrichment	pvalue	motif.name
MA1123.2	850	23103	24.63768116	9.998009313	2.464258673	1.51E-137	**TWIST1**
MA1638.1	795	22443	23.04347826	9.712388998	2.37258601	9.91E-119	**HAND2**
MA0698.1	802	25652	23.24637681	11.10110959	2.094058852	1.72E-92	**ZBTB18**
MA0091.1	579	16105	16.7826087	6.969568454	2.40798391	5.96E-86	**TAL1::TCF3**
MA1640.1	526	14850	15.24637681	6.426457096	2.372438901	2.35E-75	**MEIS2(var.2)**
MA0761.2	926	35294	26.84057971	15.27376274	1.757299767	2.96E-69	**ETV1**
MA1642.1	705	25244	20.43478261	10.92454431	1.870538673	2.05E-60	**NEUROG2(var.2)**
MA0062.3	902	36146	26.14492754	15.64247261	1.671406318	2.94E-57	**GABPA**
MA1639.1	423	12466	12.26086957	5.394761897	2.272735998	5.22E-55	**MEIS1(var.2)**
MA1113.2	466	14433	13.50724638	6.245996988	2.162544491	7.72E-55	**PBX2**

7_day15
OSKLH < OSKL

motif	observed	background	percent.observed	percent.background	fold.enrichment	pvalue	motif.name
MA0511.2	1005	12495	13.5207857	5.40731188	2.50046344	4.48E-159	**RUNX2**
MA0684.2	1114	17773	14.9872192	7.69140889	1.94856617	3.47E-103	**RUNX3**
MA1601.1	1442	28149	19.3999731	12.1817065	1.59254971	3.60E-73	**ZNF75D**
MA0809.2	1420	28103	19.1039957	12.1617996	1.57081981	3.32E-68	**TEAD4**
MA0808.1	1315	25847	17.6913763	11.1854974	1.58163519	2.95E-64	**TEAD3**
MA1121.1	1249	25009	16.8034441	10.8228462	1.55259013	1.97E-56	**TEAD2**
MA0090.3	1319	26825	17.7451904	11.6087348	1.52860675	2.97E-56	**TEAD1**
MA1141.1	1629	35543	21.915781	15.3815195	1.42481248	6.76E-52	**FOS::JUND**
MA0489.1	1652	36625	22.2252119	15.8497637	1.40224247	1.32E-48	**JUN(var.2)**
MA0462.2	1702	38036	22.8978878	16.4603853	1.39109063	3.06E-48	**BATF::JUN**

14_day15
OSKLH > OSKL

motif	observed	background	percent.observed	percent.background	fold.enrichment	pvalue	motif.name
MA0599.1	2324	65239	49.5416755	28.2327027	1.75476206	1.31E-212	**KLF5**
MA0746.2	1486	36653	31.6776807	15.8618809	1.99709485	6.06E-163	**SP3**
MA0039.4	2308	70417	49.2005969	30.4735239	1.61453585	2.71E-161	**KLF4**
MA0741.1	1668	43918	35.5574504	19.0058682	1.87086694	8.13E-160	**KLF16**
MA0079.4	1349	33768	28.7571946	14.613374	1.96786825	1.55E-139	**SP1**
MA1653.1	2004	60460	42.7201023	26.1645519	1.63274733	8.57E-136	**ZNF148**
MA0685.1	1143	27974	24.3658069	12.1059738	2.01270937	4.26E-121	**SP4**
MA1564.1	1252	32748	26.6894052	14.1719607	1.88325425	8.03E-114	**SP9**
MA1515.1	1516	43246	32.3172032	18.7150548	1.72680249	4.54E-112	**KLF2**
MA0747.1	1301	35711	27.7339586	15.4542229	1.79458773	3.92E-104	**SP8**

16_day15
OSKLH > OSKL

motif	observed	background	percent.observed	percent.background	fold.enrichment	pvalue	motif.name
MA0036.3	550	12124	25.9067358	5.24675864	4.93766486	1.77E-219	**GATA2**
MA0482.2	568	13506	26.7545926	5.84483027	4.57747981	2.01E-211	**GATA4**
MA0037.3	453	9106	21.3377296	3.94069484	5.41471251	3.48E-193	**GATA3**
MA0766.2	426	10235	20.0659444	4.42927868	4.53029621	1.26E-152	**GATA5**
MA1104.2	482	13632	22.7037211	5.89935779	3.84850724	2.07E-146	**GATA6**
MA0140.2	307	11642	14.4606689	5.03816926	2.87022292	3.93E-61	**GATA1::TAL1**
MA0648.1	209	7934	9.84455959	3.4335024	2.86720627	3.95E-41	**GSC**
MA0891.1	195	7617	9.1851154	3.29631809	2.78647726	1.02E-36	**GSC2**
MA0035.4	215	9094	10.1271785	3.93550174	2.57328778	1.48E-35	**GATA1**
MA0682.2	264	12519	12.4352332	5.41769807	2.2952983	1.62E-35	**PITX1**

Table S3. Top 10 motifs significantly higher for OSKLH than OSKL at day 2, 5 and 15

	motif	observed	background	percent.observed	percent.background	fold.enrichment	pvalue	motif.name
Day 2 OSKLH > OSKL	MA0507.1	120	27677	38.70967742	11.97744465	3.231881136	2.78E-33	**POU2F2**
	MA0627.2	110	26653	35.48387097	11.5343004	3.076378257	3.00E-28	**POU2F3**
	MA0788.1	100	23878	32.25806452	10.3333968	3.121729004	7.76E-26	**POU3F3**
	MA1115.1	103	27430	33.22580645	11.87055341	2.799010737	4.81E-23	**POU5F1**
	MA0786.1	96	25541	30.96774194	11.05307345	2.801731309	2.49E-21	**POU3F1**
	MA0787.1	93	24571	30	10.63329814	2.821325953	8.39E-21	**POU3F2**
	MA0784.1	92	24581	29.67741935	10.63762572	2.78985369	3.12E-20	**POU1F1**
	MA0792.1	81	22166	26.12903226	9.592515017	2.723897978	4.73E-17	**POU5F1B**
	MA0785.1	81	22671	26.12903226	9.811057834	2.663222733	1.71E-16	**POU2F1**
	MA0789.1	75	20487	24.19354839	8.865914245	2.72882725	8.85E-16	**POU3F4**

	motif	observed	background	percent.observed	percent.background	fold.enrichment	pvalue	motif.name
Day 5 OSKLH > OSKL	MA0039.4	307	70417	55.01792115	30.47352386	1.80543351	2.16E-33	**KLF4**
	MA0599.1	292	65239	52.3297491	28.23270266	1.853515398	4.40E-33	**KLF5**
	MA1515.1	220	43246	39.4265233	18.71505479	2.106674212	4.21E-30	**KLF2**
	MA0079.4	178	33768	31.89964158	14.61337395	2.182907361	4.64E-25	**SP1**
	MA1511.1	184	36701	32.97491039	15.88265333	2.076158795	1.65E-23	**KLF10**
	MA1107.2	223	53149	39.96415771	23.00065779	1.737522382	2.79E-19	**KLF9**
	MA0761.2	169	35294	30.28673835	15.27376274	1.982925809	2.80E-19	**ETV1**
	MA0473.3	169	36325	30.28673835	15.7199363	1.926645107	4.83E-18	**ELF1**
	MA1517.1	138	27711	24.7311828	11.99215842	2.062279526	8.25E-17	**KLF6**
	MA0062.3	158	36146	28.31541219	15.64247261	1.810162172	2.33E-14	**GABPA**

	motif	observed	background	percent.observed	percent.background	fold.enrichment	pvalue	motif.name
Day 15 OSKLH > OSKL	MA0599.1	1127	65239	51.04166667	28.23270266	1.807891624	9.26E-114	**KLF5**
	MA0039.4	1138	70417	51.53985507	30.47352386	1.69129948	3.27E-95	**KLF4**
	MA1515.1	780	43246	35.32608696	18.71505479	1.887575931	1.63E-76	**KLF2**
	MA1653.1	977	60460	44.24818841	26.16455192	1.691150245	9.12E-76	**ZNF148**
	MA0079.4	659	33768	29.84601449	14.61337395	2.042376701	2.74E-75	**SP1**
	MA0741.1	781	43918	35.37137681	19.0058682	1.861076613	7.70E-74	**KLF16**
	MA0746.2	677	36653	30.66123188	15.86188094	1.933013619	3.92E-68	**SP3**
	MA0685.1	560	27974	25.36231884	12.10597379	2.095025091	7.82E-66	**SP4**
	MA1564.1	620	32748	28.07971014	14.17196074	1.981356755	3.83E-65	**SP9**
	MA0516.2	638	34645	28.89492754	14.99290277	1.927240374	6.13E-63	**SP2**

Table S4. Karyotype analysis of primed and naive PSCs used in this experiment

	Cell line	Gender	Passage number	Karyotypic abnormalities (20 or 21 cells examined)	Representative abnormalities	Polyploid(%)
Primed	OSKLH_1	F	30	none		0%
	OSKLH_2	F	11	none		0%
	OSKLH_3	F	30	none		0%
	OSKLH_4	F	29	none		0%
	OSKLH_5	F	30	none		0%
	OSKLH_6	F	28	none		0%
	OSKLH_7	F	28	none		0%
	OSKLH_8	F	11	20/21	46,XX,t(6;7)(q23;q21)[20]	0%
	OSKLH_9	F	35	none		0%
	OSKLH_10	F	28	none		0%
Naive	Reset H9 ESC	F	85	19/20	46,XX,+21,der(21;21)(q10;q10)[5],<br>14 other abnormalities	32.3%
	nOSKL_1	F	16	18/20	48,XX,+11,+21[5],<br>13 other abnormalities	44.9%
	nOSKL_2	F	16	11/20		24.1%
	nOSKLH_1	F	16	14/20	46,XX,add(15)(p11.2)[2],<br>12 other abnormalities	48.5%
	nOSKLH_2	F	16	8/20		24.4%

Table S5. SNP genotyping array results of naive iPSCs generated in this study

No.	1		2	3	4	5	6	7	8		9
Type of CNV	gain	gain	loss	loss	gain	gain	loss	loss	gain	gain	gain
Location	chr1: 202,818,754-202,849,770	chr1: 203,144,833-203,158,972	chr1: 229,575,709-230,062,961	chr2: 11,451,984-11,796,774	chr4: 148,550,590-148,659,137	chr6: 162,803,457-162,847,889	chr7: 146,879,634-147,223,640	chr10: 24,647,899-24,736,083	chr15: 60,220,932-60,259,176	chr15: 60,585,694-60,638,256	chr18: 37,340,664-38,067,013
Size (bp)	31,017	14,140	487,253	344,791	108,548	44,433	344,007	88,185	38,245	52,563	726,350
t2iLGö+Y OSKL_1 (p12)	-	-	-	-	-	-	-	-	-	-	-
t2iLGö+Y OSKL_2 (p12)	-	-	-	-	-	-	-	-	-	-	-
t2iLGö+Y OSKL_3 (p12)	-	-	-	-	-	-	-	-	-	-	-
t2iLGö+Y OSKLH_1 (p12)	-	-	-	-	-	-	-	-	-	-	-
t2iLGö+Y OSKLH_2 (p12)	-	-	-	-	-	-	-	-	-	-	-
t2iLGö+Y OSKLH_3 (p12)	-	-	-	-	-	-	-	-	-	-	-
PXGLY OSKL_1 (p13)	-	-	-	-	-	-	-	-	-	-	-
PXGLY OSKL_2 (p13)	-	-	-	-	-	-	-	-	-	-	-
PXGLY OSKL_3 (p13)	-	-	-	-	-	-	-	-	-	-	-
PXGLY OSKLH_1 (p13)	-	-	-	-	-	-	-	-	-	-	-
PXGLY OSKLH_2 (p13)	-	-	-	-	-	-	-	-	-	-	-
PXGLY OSKLH_3 (p13)	-	-	-	-	-	-	-	-	-	-	-
t2iLGö+Y → PXGLY OSKL_1 (p13)	-	-	-	-	-	-	-	-	-	-	-
t2iLGö+Y → PXGLY OSKL_2 (p13)	-	-	-	-	-	-	-	-	-	-	-
t2iLGö+Y → PXGLY OSKL_3 (p13)	-	-	-	-	-	-	-	-	-	-	-
t2iLGö+Y → PXGLY OSKLH_1 (p13)	-	-	-	-	-	-	-	-	-	-	-
t2iLGö+Y → PXGLY OSKLH_2 (p13)	-	-	-	-	-	-	-	-	-	-	-
t2iLGö+Y → PXGLY OSKLH_3 (p13)	-	-	-	-	-	-	-	-	-	-	-
t2iLGö+Y → AXGY OSKL_1 (p13)	-	-	-	-	-	-	-	-	-	-	-
t2iLGö+Y → AXGY OSKL_2 (p17)	-	-	-	-	-	-	-	-	-	-	○
t2iLGö+Y → AXGY OSKL_3 (p17)	○	○	-	-	-	-	-	-	-	-	-
t2iLGö+Y → AXGY OSKLH_1 (p17)	-	-	-	-	-	-	-	-	-	-	-
t2iLGö+Y → AXGY OSKLH_2 (p17)	-	-	-	-	-	-	-	-	-	-	-
t2iLGö+Y → AXGY OSKLH_3 (p17)	-	-	-	-	-	-	-	-	-	-	-
5iLA → AXGY OSKL_1 (p17)	-	-	-	-	-	-	-	-	-	-	-
5iLA → AXGY OSKL_2 (p17)	-	-	-	-	-	-	-	-	-	-	-
5iLA → AXGY OSKL_3 (p17)	-	-	-	-	-	○	-	-	○	○	-
5iLA → AXGY OSKLH_1 (p17)	-	-	-	-	-	-	-	-	-	-	-
5iLA → AXGY OSKLH_2 (p17)	-	-	○	○	○	-	-	○	-	-	-
5iLA → AXGY OSKLH_3 (p17)	-	-	-	-	-	-	○	-	-	-	-

SUPPLEMENTARY EXPERIMENTAL PROCEDURES

Construction of SeV-*H1FOO*, SeV-*H1FOO-DD*, and SeV-*DD-H1FOO* vectors

The insert sequence containing the open reading frame of *H1FOO*, *H1FOO-DD*, and *DD-H1FOO* genes were amplified by PCR from cDNAs and inserted in the NotI site of the plasmids containing the F-defecive SeV vector backbone. The gene specific primers were designed to introduce NotI sites on both ends of the amplified fragment and SeV-specific transcriptional regulatory signal sequences were also added after the coding sequence. We obtained DD sequence information from a previous report (Banaszynski et al., 2006). In *H1FOO-DD, DD* was inserted downstream of the C-terminus of *H1FOO* and in *DD-H1FOO*, upstream of the N-terminus of *H1FOO.* The TS15 mutations are reported elsewhere (Schlaeger, 2018).The plasmids pSeV18+H1FOO/TS15ΔF, pSeV18+H1FOO-DD/TS15ΔF, and pSeV18+DD-H1FOO/TS15ΔF were constructed according to the previously reported methods (Inoue et al., 2003) . SeV-H1FOO, SeV-H1FOO-DD, and SeV-DD-H1FOO vectors were recovered from these plasmids and propagated as previously described (Komuta et al., 2016). When stabilizing H1FOO-DD to prevent degradation, 1 μM Shield1 (Takara) was added to the cell culture medium.

iPSC colony formation assay

For both naive and primed iPSCs, iPSC colony counts were performed on day 14 after the SeV vector infection. We visually counted AP-positive colonies using an optical microscope (Olympus) after cells were fixed in 4% paraformaldehyde (Nacalai Tesque) for 15 minutes at room temperature and then stained using the Alkaline Phosphatase Assay Kit (Sigma) according to the manufacturer's protocol.

Immunocytochemistry

The cells plated on glass bottomed dishes (AGC) were washed once with phosphate-buffered saline (PBS) and fixed with 4% paraformaldehyde (Nacalai Tesque) for 15min at room temperature. The cells were permeabilized with 0.5% Triton X-100 (Nacalai Tesque) in PBS for 10 min at room temperature. After blocking with ImmunoBlock (KAC) for 15 min, the cells were incubated at room temperature for 60 min with the primary antibodies which were diluted in ImmunoBlock. After being washed twice with PBS, the samples were exposed to fluorescence-conjugated secondary antibodies along with 4',6-diamidino-2-phenylindole (DAPI, Invitrogen) for 60 min at room temperature. Images were obtained using a BZ-X710 imaging system (KEYENCE). The list of antibodies used in this study is provided in the key resources table.

Trilineage differentiation

We used STEMdiff Trilineage Differentiation Kit (STEMCELL) to differentiate iPSCs into trilineage. 4x10^5 cells for ectoderm, 2x10^5 cells for mesoderm, and 4x10^5 cells for endoderm were seeded into 6 wells coated with Matrigel (Corning). For ectoderm differentiation, we started

culturing in ectoderm medium instead of mTeSR1 (STEMCELL) from the first day. To promote differentiation, we added 10 uM SB431542 (Wako) to the ectoderm medium. In mesoderm and endoderm differentiation, we cultured iPSCs in mTeSR1 on the first day. We collected and analyzed ectoderm differentiated cells on day 7 and mesoderm and endoderm differentiated cells on day 5.

Cardiomyocyte differentiation

Four days prior to the start of differentiation, 4x10^4 iPSCs were seeded, and maintained by changing iPSC media every other day. On Day 0, we replaced the medium with RPMI (Wako) containing 2 % B-27 supplement minus insulin (B-27MI, Gibco), 6 uM CHIR (Wako) and 1 ng/ml BMP4 (R&D). 24 hours later on Day 1, we washed cells with PBS and replaced with RPMI containing only B-27MI. On Day 3, we replaced the medium with RPMI containing B-27MI and 5 uM IWR-1 (Sigma). On Day 7, the medium was replaced with MEM alpha (Gibco) containing 5 % FBS, and cells were collected and analyzed on Day 10.

Naive PSC-derived trophectoderm (TE) differentiation

For TE differentiation, naive PSCs were dissociated, and iMEF feeder cells were removed as described above. 500,000 cells were plated in laminin 511-E8 (0.15 μg/cm^2 iMatrix 511; Nippi)-coated 6 wells with initial TE differentiation medium: NDiff227, 2 μM A83-01 (Wako), 2 μM PD0325921 (Sigma) and 10 ng/mL BMP4 (R&D). The next day, the medium was changed to NDiff227, 2 μM A83-01 (Wako), 2 μM PD0325921 (Sigma) and 1 μg/mL JAK inhibitor I (Sigma). At day 2, the medium was changed again. At day 3, we harvested the cells using Accutase (Innovative Cell Technologies) for 30 min and analyzed TACSTD2 and HAVCR1 expression by flow cytometry.

Flow cytometry

Dissociated cells were stained on ice for 20 min with fluorescent conjugated antibodies. Analyses were performed using the BD LSR Fortessa (BD Biosciences) flow cytometer equipped with FACS Diva software (BD biosciences) and Gallios flow cytometer (Beckman Coulter). The data were analyzed using FlowJo software (LLC).

RNA and DNA extraction and real-time quantitative PCR (qPCR)

When extracting nucleic acids from naive PSCs, iMEFs were removed by incubating naive PSCs for 2 hours at 37°C on a gelatin-coated dish before sampling for nucleic acid extraction. Total RNA and DNA were extracted from cell lysates using the AllPrep DNA/RNA Mini Kit (QIAGEN), and the RNA was incubated with RNase-Free DNase Set (QIAGEN) to remove genomic DNA. For qPCR, the reverse transcription reaction was performed with 1 μg of DNase-treated RNA using PrimeScript RT Master Mix (Takara) containing oligo dT primer and random 6 mers. qPCR analysis was performed on StepOne Plus (Applied Biosystems) or QuantStudio3 (Applied Biosystems) using

TaqMan Fast Advanced Master Mix (Applied Biosystems) or Fast SYBR Green Master Mix (Applied Biosystems), and Scorecard analysis was performed on QuantStudio 12K (Applied Biosystems) or StepOne Plus (Applied Biosystems) using TaqMan Gene Expression Master Mix (Applied Biosystems) according to the manufacturer's protocol.

Western blot analysis

The cells were lysed using M-PER Mammalian Protein Extraction Reagent (Thermo) containing protease inhibitor (Sigma) and phosphatase inhibitor cocktail 2 and 3 (Sigma). Protein concentration was determined using Pierce BCA Protein Assay Kit (Thermo).

For detection, we used WES or JESS system (ProteinSimple) which performs protein separation and detection using an automated capillary electrophoresis system. Signals were detected with an HRP-conjugated secondary anti-rabbit antibody and were visualized using Compass software (ProteinSimple).

Cell metabolism analysis

Oxygen consumption rate and extracellular acidification rate were measured using a Seahorse XF96 Analyzer (Agilent) and Seahorse XF Cell Mito Stress Test Kit (Agilent). PSCs were dissociated and cells were incubated on gelatin for 2 hours at 37 °C to remove feeder cells. Seahorse plate was coated with Matrigel (Corning) for 1hr at 37 °C prior cell seeding. Cells were seeded at 100,000 cells per well and incubated overnight. The next day, culture media were exchanged for XF Base Medium (Agilent) supplemented with 2 mM pyruvate, 20 mM glucose and 2 mM Glutamax (Gibco) with an adjusted pH of 7.4 and cells incubated at 37 °C in atmospheric CO_2 incubator for 1 hr. During the mito stress kit experiment, Oligomycin (6 μM), FCCP (2 μM), Rotenone and Antimycin-A (1 μM) were injected at indicated time points. Metabolic profiling was performed by mapping the OCR and ECAR at eighth time point from the beginning of the analysis as indicated in Figure S5D. The spare respiratory capacity was calculated as the difference between basal and FCCP-induced OCR (Nicholas et al., 2017).

RNA-FISH

Dissociated naive iPSCs were incubated on gelatin for 2 hours at 37 °C to remove iMEF feeder cells. Then the cells were seeded on Matrigel-coated slides in PSC medium. The next day, the cells were fixed in 4 % paraformaldehyde for 15 min at room temperature. The slides were treated with 0.2 M HCl for 20 min, permeabilized with 0.2% Triton X-100 for 10 min, digested with pepsin solution (0.005% in 0.1 M HCl) at 37 ºC for 2-6 min, and dehydrated. Bacterial artificial chromosomes (BACs) RP11-155O24 and RP11-256P2 were used to generate *HUWE1* and *UTX* RNA FISH probes, respectively. BAC DNAs were labelled by nick-translation with Cy5-dUTP (RP11-155O24) and Cy3-dUTP (RP11-256P2). The labelled probes and the *XIST* RNA FISH probe (Chromosome Science Labo) were mixed with sonicated salmon sperm DNA and Cot-1 DNA in hybridization

solution. The probes were denatured at 85ºC for 10 min, applied to the pretreated slides, covered with cover slips, and hybridized at 37ºC overnight. The slides were then washed with 50% formamide / 2xSSC at 37ºC for 20 min, 1xSSC for 15 min at room temperature, counterstained using DAPI, and mounted. The FISH images were captured with the CW4000 FISH application program (Leica Microsystems Imaging Solution) using a cooled CCD camera mounted on a Leica DMRA2 microscope. We examined the expression of *UTX*, *HUWE1*, and *XIST* in 100 cells per clone.

Chromosome analysis

The samples in exponentially growing phase were incubated with final concentration of 0.02 μg/mL Metaphase Arresting Solution (Genial Genetic Solutions) for 90 min at 37 ℃. The cells were collected in tubes and subjected to hypotonic treatment with 0.075 M KCl solution for 30 min at 37 ℃. After fixing the cells with Carnoir's fixative (methanol: acetic acid ratio 3 : 1), the cell suspension was dropped a few drops on the glass slides and air dried.

For conventional Giemsa staining (non-banding technique), after staining the slides with Giemsa solution/phosphate buffer, the slides were observed by BX-51 or BX-53 microscopy (OLYMPUS). A total of 30 metaphases were counted for the chromosomal counts and these metaphase chromosomes were classified into distinguishable groups (A~G) based on morphological features.

For GTG method (G-banding), after trypsin treatment and Giemsa staining, the slides were observed by AxioImagerZ2 microscopy (CarlZeiss microscopy) equipped with CoolCube1m CCD camera (MetaSystems) and Metafer Slide Scanning System (MetaSytems). At least 20 metaphases were analyzed based on G-band by using Ikaros Karyotyping System (MetaSystems).

Any samples suspected to be chromosomal abnormality and required detail analysis were further analyzed by mBAND (multicolor chromosome banding) method using an appropriate mBAND Probe kit (MetaSystems). For mBAND analysis, we used Isis FISH Imaging System.

SNP genotyping array

Copy number variation (CNV) was evaluated with SNP genotyping array. Genomic DNA was hybridized onto the Infinium OmniExpress24 v1.4 DNA Analysis Kit (Illumina), and intensities were scanned by iScan (Illumina) following the manufacturer's protocol. After exporting a final report using GenomeStudio (2.0.4) (Illumina), CNV analysis was performed with PennCNV (1.0.3) (Wang et al., 2007), Mosaic Alteration Detection-MAD (1.0.1) (González et al., 2011) and GWAS tools (1.16.1) (Gogarten et al., 2012). Only CNVs in test samples against control samples were reported. Log R Ratios and B-Allele Frequencies were visualized with GenomeStudio.

RNA sequencing and data analysis

RNA sequencing libraries were made from 100 ng of total RNA as starting materials with the TruSeq Stranded mRNA LT Sample Prep Kit Set A (Illumina) or TruSeq Stranded Total RNA Library Prep Gold (Illumina) following the manufacturer's protocol. For Hiseq2500, clusters were generated with

the HiSeq PE Cluster Kit v4-cBot (Illumina) using illumina cBot. Sequencing was performed with the HiSeq SBS Kit v4 using HiSeq2500 (2 x 126 PE mode). NovaSeq 6000 (2 x 101 PE mode) with the NovaSeq 6000 S1 Reagent Kit v1.5 (Illumina) and NextSeq 500 (76 SE mode) with the NextSeq 500/550 High Output Kit v2.5 (Illumina) was also used for sequencing. FASTQ files were generated from bcl files using bcl2fastq v2.17.1.14 (Illumina) and processed using ENCODE long-rna-seq-pipeline v2.3.4. Briefly, the sequenced reads were mapped to the human reference genome (GRCh38) using TopHat 2.1.1 (Kim et al., 2013) or STAR 2.5.1b (Dobin et al., 2013) with GENCODE v24 gene annotations, the normalized gene expression data was calculated using RSEM 1.2.23 (Li and Dewey, 2011), and the gene count data was obtained using featureCounts bundled in Subread 1.5.1 (Liao et al., 2014). For the characterization of our PSC lines, the data sets of GSE59435 (Theunissen et al., 2014) and GSE75868 (Theunissen et al., 2016) obtained from GEO and supplemental data in Yan *et al*. (Yan et al., 2013) and Takashima *et al*. (Takashima et al., 2014) were used. The expression values of 4,720 genes included in all data were normalized by quantile among samples, and z-scores for each gene were used for the PCA. Log2-scaled, quantile normalized FPKM values were used for the expression heatmap of PSC markers.

DNA methylation analysis

The bisulfite conversion of 500 ng genomic DNA was performed using the EZ DNA Methylation Kit (Zymo Research), and the global DNA methylation status was profiled using Infinium Human Methylation 450K or EPIC BeadChip Kit (illumina) according to the manufacturer's protocols. After exporting the DNA methylation values using GenomeStudio V2011.1, data processing was conducted using the "minfi" package in R 3.6.3 (Aryee et al., 2014). In total, 424,444 probes common between 450K and EPIC and not located at known SNP sites were used for the PCA of PSC samples.

Single cell RNA-seq analysis

Single cell RNA-seq libraries were prepared according to the manufacturer's protocol using the 10x Genomics Chromium Next GEM Single Cell 3' Kit v3 and Single Index Kit, where the number of target cells was 2500. The libraries were sequenced with Hiseq2500, where clusters were generated with the HiSeq PE Cluster Kit v4-cBot (Illumina) using illumina cBot and sequencing was performed with the HiSeq SBS Kit v4 (28-8-0-91 cycles). Raw sequencing data was converted to the standard FASTQ files by executing cellranger mkfastq (3.1.1, 10x Genomics). Gene counts of each sample were generated by executing cellranger count (3.1.1, 10x Genomics) with refdata-cellranger-GRCh38-3.0.0 as the reference dataset. Single cell RNA-seq data processing including quality control, count normalization, and clustering was performed using Seurat (Butler et al., 2018) version 3.2.2. Cells with gene numbers less than 4000 or larger than 11000 were removed. Cells with higher proportion of mitochondrial mRNA (proportion of mitochondrial mRNA > 25) were also removed. After the quality control, each count matrix was log-normalized by pool, and then merged. Principal

component analysis (PCA) of the combined object was performed by RunPCA function using 2000 variable genes selected by FindVariableFeatures function with vst method. For the visualization, uniform manifold approximation and projection (UMAP) dimensional reduction was used with 30 dimensions of PCA to use as input features. To detect the clusters, k-nearest neighbor (kNN) graph was constructed using Seurat function, FindNeighbors and FindClusters with default parameters other than resolution = 0.5. Data visualization was performed using DimPlot, FeaturePlot, DotPlot and VlnPlot functions in Seurat.

Single cell ATAC-seq analysis

Single cell ATAC-seq libraries were prepared according to the manufacturer's protocol using the 10x Genomics Chromium Single Cell ATAC Reagent Kit v1, where the number of target cells was 2500. The libraries were sequenced with Hiseq2500, where clusters were generated with the HiSeq PE Cluster Kit v4-cBot (Illumina) using illumina cBot and sequencing was performed with the HiSeq SBS Kit v4 (50-8-16-50 cycles). Raw sequencing data was converted to the standard FASTQ files by executing cellranger-atac mkfastq (1.1.0, 10x Genomics). Fastq files from single cell ATAC-seq were mapped by CellRanger using the hg38 reference (refdata-cellranger-atac-GRCh38-1.2.0). Mapping was performed using cellranger-atac count function by pool and then the data aggregated by cellranger-atac aggr function. The peak size of aggregated 231,076 peak regions in peaks.bed output were re-sized into peak-centered 500 bp non-redundant regions and then all pools were re-mapped by cellranger-atac reanalyze function using the common 500bp regions.
Count data and metadata from CellRanger output was processed following the Signac "Guided analyses" (https://satijalab.org/signac/articles/overview.html) using Seurat (v3.2.2), Signac (Stuart et al., 2021) (v1.1.0), TFBSTools (Tan and Lenhard, 2016) (v1.22.0) and chromVAR (Schep et al., 2017) (c1.6.0). For quality control, the mapping rate of reads in transcript start site (TSS) regions (TSS.enrichment) was checked and all data were retained because all cells had TSS.enrichment more than 2%. The median of ATAC counts per cell were 28,455 ± 7,526.4 (standard deviation). The percent reads in peak regions (pct_reads_in_peaks) were calculated as % of (peak region fragments) /(passed filters).
All sample pools were merged and normalized by term frequency inverse document frequency (TFI-DF) normalization using RunTFIDF function with default parameters (Stuart et al., 2019). For the visualization, the singular value decomposition and UMAP dimensional reduction was performed using latent semantic indexing (LSI) components 2 to 50 (Cusanovich et al., 2018). To detect the clusters, kNN graph was constructed using Seurat function, FindNeighbors using LSI components 2 to 30 and FindClusters with smart local moving algorithm (algorithm = 3) (Butler et al., 2018). Data visualization was performed using DimPlot, FeaturePlot, DotPlot and VlnPlot functions in Seurat.

Cluster prediction using scRNA-seq data

To integrate the scATAC-seq data with scRNA-seq clusters, we computed gene activity counts for

each gene in each cell using GeneActivity function in Signac with default parameters. This gene activity counts were anchored with the cell type information of scRNA-seq data using FindTransferAnchors with a canonical correlation analysis reduction method and TransferData function using the LSI components 2 to 30 for weighted reduction.

Motif enrichment analysis by chromVAR

Motif enrichment analysis of scATAC-seq data was performed using AddMotifs function of TFBSTools package using a matrix set from JASPAR2020 datasets (Fornes et al., 2020). Differential accessibility to the motifs between groups were calculated by constructing a logistic regression model using FindMarkers function. Motif activities of individual cells were calculated using RunChromVAR function in Signac. Motif activities were vidualized using FeaturePlot and VlnPlot in Seurat.

SUPPLEMENTARY REFERENCES

Aryee, M.J., Jaffe, A.E., Corrada-Bravo, H., Ladd-Acosta, C., Feinberg, A.P., Hansen, K.D., and Irizarry, R.A. (2014). Minfi: a flexible and comprehensive Bioconductor package for the analysis of Infinium DNA methylation microarrays. Bioinformatics *30*, 1363-1369.

Banaszynski, L.A., Chen, L.C., Maynard-Smith, L.A., Ooi, A.G., and Wandless, T.J. (2006). A rapid, reversible, and tunable method to regulate protein function in living cells using synthetic small molecules. Cell *126*, 995-1004.

Butler, A., Hoffman, P., Smibert, P., Papalexi, E., and Satija, R. (2018). Integrating single-cell transcriptomic data across different conditions, technologies, and species. Nature biotechnology *36*, 411-420.

Cusanovich, D.A., Hill, A.J., Aghamirzaie, D., Daza, R.M., Pliner, H.A., Berletch, J.B., Filippova, G.N., Huang, X., Christiansen, L., DeWitt, W.S.*, et al.* (2018). A Single-Cell Atlas of In Vivo Mammalian Chromatin Accessibility. Cell *174*, 1309-1324.e1318.

Dobin, A., Davis, C.A., Schlesinger, F., Drenkow, J., Zaleski, C., Jha, S., Batut, P., Chaisson, M., and Gingeras, T.R. (2013). STAR: ultrafast universal RNA-seq aligner. Bioinformatics *29*, 15-21.

Fornes, O., Castro-Mondragon, J.A., Khan, A., van der Lee, R., Zhang, X., Richmond, P.A., Modi, B.P., Correard, S., Gheorghe, M., Baranašić, D.*, et al.* (2020). JASPAR 2020: update of the open-access database of transcription factor binding profiles. Nucleic Acids Res *48*, D87-d92.

Inoue, M., Tokusumi, Y., Ban, H., Kanaya, T., Tokusumi, T., Nagai, Y., Iida, A., and Hasegawa, M. (2003). Nontransmissible virus-like particle formation by F-deficient sendai virus is temperature sensitive and reduced by mutations in M and HN proteins. J Virol *77*, 3238-3246.

Kim, D., Pertea, G., Trapnell, C., Pimentel, H., Kelley, R., and Salzberg, S.L. (2013). TopHat2: accurate alignment of transcriptomes in the presence of insertions, deletions and gene fusions. Genome biology *14*, R36.

Komuta, Y., Ishii, T., Kaneda, M., Ueda, Y., Miyamoto, K., Toyoda, M., Umezawa, A., and Seko, Y. (2016).

In vitro transdifferentiation of human peripheral blood mononuclear cells to photoreceptor-like cells. Biol Open *5*, 709-719.

Li, B., and Dewey, C.N. (2011). RSEM: accurate transcript quantification from RNA-Seq data with or without a reference genome. BMC Bioinformatics *12*, 323.

Liao, Y., Smyth, G.K., and Shi, W. (2014). featureCounts: an efficient general purpose program for assigning sequence reads to genomic features. Bioinformatics *30*, 923-930.

Nicholas, D., Proctor, E.A., Raval, F.M., Ip, B.C., Habib, C., Ritou, E., Grammatopoulos, T.N., Steenkamp, D., Dooms, H., Apovian, C.M., *et al.* (2017). Advances in the quantification of mitochondrial function in primary human immune cells through extracellular flux analysis. PloS one *12*, e0170975.

Schep, A.N., Wu, B., Buenrostro, J.D., and Greenleaf, W.J. (2017). chromVAR: inferring transcription-factor-associated accessibility from single-cell epigenomic data. Nature methods *14*, 975-978.

Schlaeger, T.M. (2018). Nonintegrating Human Somatic Cell Reprogramming Methods. Adv Biochem Eng Biotechnol *163*, 1-21.

Stuart, T., Butler, A., Hoffman, P., Hafemeister, C., Papalexi, E., Mauck, W.M., 3rd, Hao, Y., Stoeckius, M., Smibert, P., and Satija, R. (2019). Comprehensive Integration of Single-Cell Data. Cell *177*, 1888-1902.e1821.

Stuart, T., Srivastava, A., Madad, S., Lareau, C.A., and Satija, R. (2021). Single-cell chromatin state analysis with Signac. Nature methods *18*, 1333-1341.

Takashima, Y., Guo, G., Loos, R., Nichols, J., Ficz, G., Krueger, F., Oxley, D., Santos, F., Clarke, J., Mansfield, W., *et al.* (2014). Resetting transcription factor control circuitry toward ground-state pluripotency in human. Cell *158*, 1254-1269.

Tan, G., and Lenhard, B. (2016). TFBSTools: an R/bioconductor package for transcription factor binding site analysis. Bioinformatics *32*, 1555-1556.

Theunissen, T.W., Friedli, M., He, Y., Planet, E., O'Neil, R.C., Markoulaki, S., Pontis, J., Wang, H., Iouranova, A., Imbeault, M., *et al.* (2016). Molecular Criteria for Defining the Naive Human Pluripotent State. Cell stem cell.

Theunissen, T.W., Powell, B.E., Wang, H., Mitalipova, M., Faddah, D.A., Reddy, J., Fan, Z.P., Maetzel, D., Ganz, K., Shi, L., *et al.* (2014). Systematic identification of culture conditions for induction and maintenance of naive human pluripotency. Cell stem cell *15*, 471-487.

Yan, L., Yang, M., Guo, H., Yang, L., Wu, J., Li, R., Liu, P., Lian, Y., Zheng, X., Yan, J., *et al.* (2013). Single-cell RNA-Seq profiling of human preimplantation embryos and embryonic stem cells. Nature structural & molecular biology *20*, 1131-1139
